# Deep brain stimulation of the thalamus restores signatures of consciousness in a nonhuman primate model

**DOI:** 10.1126/sciadv.abl5547

**Published:** 2022-03-18

**Authors:** Jordy Tasserie, Lynn Uhrig, Jacobo D. Sitt, Dragana Manasova, Morgan Dupont, Stanislas Dehaene, Béchir Jarraya

**Affiliations:** 1 Cognitive Neuroimaging Unit, CEA, INSERM, Université Paris-Saclay, NeuroSpin Center, 91191 Gif/Yvette, France.; 2 Department of Anesthesiology and Critical Care, Necker Hospital, AP-HP, Université de Paris, Paris, France.; 3 Sorbonne Université, Institut du Cerveau–Paris Brain Institute–ICM, Inserm, CNRS, APHP, Hôpital de la Pitié Salpêtrière, Paris, France.; 4 Université de Paris, Paris, France.; 5 Collège de France, Université Paris-Sciences-Lettres (PSL), Paris, France.; 6 University of Versailles Saint-Quentin-en-Yvelines, Université Paris-Saclay, Versailles, France.; 7 Foch Hospital, Suresnes, France.

## Abstract

Loss of consciousness is associated with the disruption of long-range thalamocortical and corticocortical brain communication. We tested the hypothesis that deep brain stimulation (DBS) of central thalamus might restore both arousal and awareness following consciousness loss. We applied anesthesia to suppress consciousness in nonhuman primates. During anesthesia, central thalamic stimulation induced arousal in an on-off manner and increased functional magnetic resonance imaging activity in prefrontal, parietal, and cingulate cortices. Moreover, DBS restored a broad dynamic repertoire of spontaneous resting-state activity, previously described as a signature of consciousness. None of these effects were obtained during the stimulation of a control site in the ventrolateral thalamus. Last, DBS restored a broad hierarchical response to auditory violations that was disrupted under anesthesia. Thus, DBS restored the two dimensions of consciousness, arousal and conscious access, following consciousness loss, paving the way to its therapeutical translation in patients with disorders of consciousness.

## Teaser

Thalamic stimulation restores behavioral and brain-imaging signatures of consciousness in anesthetized macaques.

## INTRODUCTION

Consciousness can be studied at two different levels: arousal, emerging from brainstem ascending reticular systems and basal forebrain, and awareness, mainly characterized by the conscious access to a specific piece of information. Several theories have been proposed for the cerebral mechanisms of consciousness (*1*). The thalamocortical loops and sensorimotor couplings (TCL) theory stipulates that consciousness is “determined by synchronous activity in the thalamocortical system” (*2*). According to the global neuronal workspace (GNW) theory, awareness occurs when a specific piece of information is made available to a broad cortical network interconnected through long-distance corticocortical axons (*3*, *4*) under the coordination of nuclei of the brainstem ascending reticular formation and the projecting thalamocortical neurons (*5*). Explicit neural network–based simulation of the GNW incorporates both thalamic and cortical neurons (*4*, *6*, *7*). Deep brain nuclei such as the thalamus and cerebellar nuclei have been identified as key structures for the persistent representation of information (or reverberation) within the cortex (*8*, *9*). Thus, conscious access is achieved by corticocortical loops and corticosubcortical loops (*10*). The dendritic information theory of consciousness, a recent proposal compatible with GNW, rests on evidence for a new cellular integration mechanism of consciousness in which cortical pyramidal neurons act as gates that control the global propagation of input information arising in part from thalamocortical connectivity (*11*–*13*). The key role of thalamocortical interplay for conscious access has also been stressed by other theories of consciousness (*2*, *12*, *14*) and of anesthesia-induced consciousness loss (*15*–*18*). The information integration theory (IIT) of consciousness, for instance, predicts a “breakdown in connectivity within the corticothalamic network” during sleep or general anesthesia (*19*).

The central thalamus (CT) comprises multiple intra- and paralaminar thalamic nuclei, which are interposed between the brainstem/basal forebrain arousal systems and the cortex (*20*). Central thalamic neurons play a key role in arousal regulation through anatomical connections with large-scale cortical networks (*20*). In their pioneering work, Moruzzi and Magoun (*21*) stimulated the brainstem reticular formation of anesthetized cats and induced a cortical desynchronization evidenced by electroencephalography (EEG), which they hypothesized to be mediated by intralaminar thalamic nuclei. In support of this hypothesis, histological studies could identify cortical projections of intralaminar nuclei neurons that have a monosynaptic excitatory input from the reticular formation (*22*) and demonstrate additional input from other arousal systems to the intralaminar nuclei, specifically from locus coeruleus (*23*) and basal forebrain (*24*). Thus, intralaminar nuclei play a critical role in regulating arousal (*20*), and some anesthetics suppress consciousness through a disruption of intralaminar nuclei functional connectivity (*25*). Using rodent models, Alkire and colleagues (*26*–*28*) originally demonstrated that the loss of consciousness induced by anesthesia could be reversed by means of direct manipulation of the central medial thalamus. Recently, electrical stimulation of CT neurons in nonhuman primates was found to enhance endogenous arousal and ameliorate cognitive responses to a visuomotor task (*29*) and eventually reverse the effects of anesthesia (*30*). Redinbaugh and colleagues (*31*) electrically stimulated the central lateral thalamus of anesthetized macaques and could modulate vigilance by controlling layer-specific cortical interactions. They demonstrated that thalamic stimulation restored frontoparietal coherence in both feedforward and feedback pathways. This finding is in line with recent work that investigated the cellular mechanism by which anesthesia suppresses the feedback pathway and leads to consciousness loss (*11*, *32*, *33*). Thus, electrical modulation of CT holds potential for controlling not only arousal but also conscious access during consciousness loss.

In chronic disorders of consciousness, severe large brain injuries can lead to a vegetative state/unresponsive wakefulness syndrome or a minimally conscious state. Currently, there are only few therapeutic options with limited impact for these patients (*34*–*37*). Brain-imaging studies suggest that the restoration of corticocortical and thalamocortical connectivity may be key for the recovery from chronic disorders of consciousness (*38*). Deep brain stimulation (DBS) allows the electrical stimulation of the central nervous system in a target-specific manner (*39*). It has been reported that thalamic DBS can modulate arousal in anesthetized rodents (*40*, *41*) or monkeys (*31*) and improve behavioral aspects in patients with disorders of consciousness by restoring global cortical activity (*36*, *42*–*44*). However, a clear demonstration that DBS can restore both arousal and awareness aspects of consciousness is currently lacking. Here, we propose such a demonstration using thalamic DBS in a nonhuman primate model of consciousness loss, as a crucial step toward the development of specific therapeutical interventions for patients with chronic disorders of consciousness.

We previously developed a nonhuman primate model of loss of consciousness based on EEG-monitored anesthesia (*18*, *45*, *46*). In this model, using simultaneous functional magnetic resonance imaging (fMRI), we could demonstrate several brain-imaging signatures of consciousness as opposed to general anesthesia. First, during the resting state, the conscious brain is the seat of quickly changing dynamics with a rich and variable repertoire of global brain state, whereas the fMRI activity of the anesthetized brain exhibits a more static functional connectivity matrix that resembles the fixed anatomical connectivity matrix (*45*, *46*). This signature, first identified in nonhuman primates, was later confirmed in patients with disorders of consciousness (*47*). A second signature of consciousness, and more specifically conscious access, was obtained in both humans and macaques using a passive auditory task, called the “local-global” paradigm (*48*), which dissociates two hierarchical levels of auditory regularities (*48*, *49*). In nonhuman primates, violations of a global regularity in a sequence of tones induced a “global” effect that activated a widespread cortical network in awake monkeys (*50*), while this activity was massively disorganized or absent under anesthesia (*18*). Here, we investigated the potential of thalamic DBS to restore those two signatures of consciousness in deeply anesthetized nonhuman primates. Under propofol anesthesia, the implanted DBS leads stimulated either the CT (CT-DBS) or a control structure, the ventrolateral (VL) thalamic nucleus (VL-DBS). We monitored the effects of DBS on both behavior and brain activity using fMRI and EEG. We investigated both the cortical dynamics of spontaneous resting-state activity (*45*–*47*) and the brain evoked activity by the local-global paradigm (*18*, *48*, *50*). Our results provide evidence of the specificity and the efficacy of central thalamic DBS to restore both arousal and awareness.

## RESULTS

### Combining electrical thalamic DBS and EEG-fMRI recording in anesthetized macaques

**Fig. 1.**
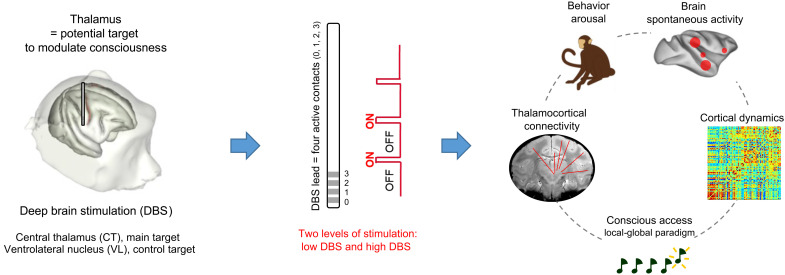
Experimental design. Schematic representation of the study design, followed by the experimental steps and modalities of investigation.

**Fig. 2.**
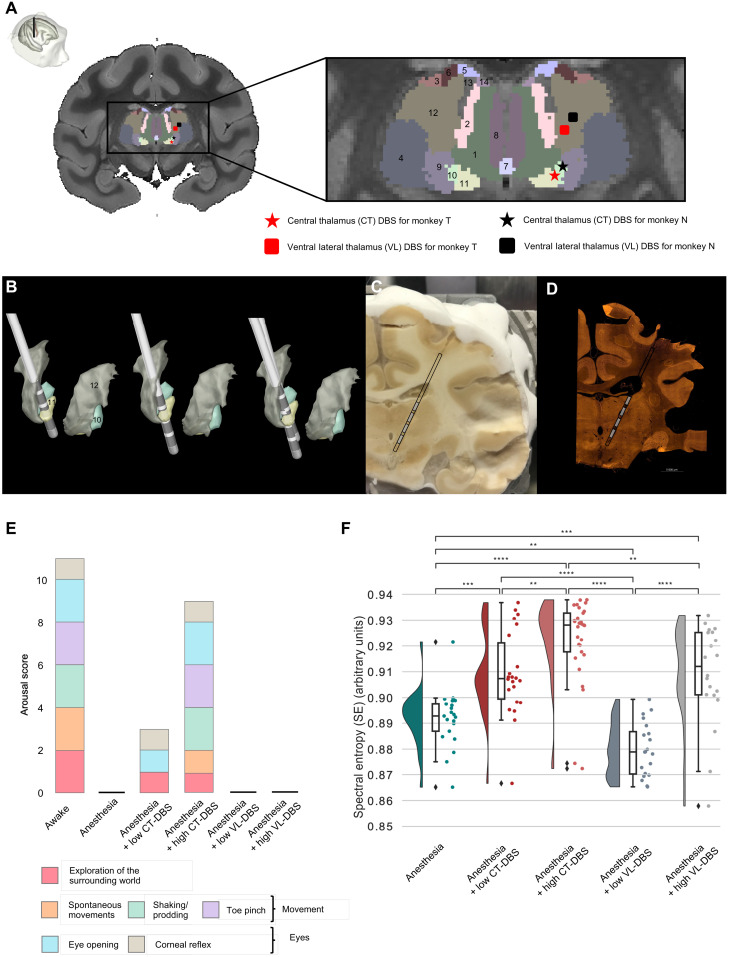
DBS of the CT restores arousal and cortical entropy in anesthetized macaques. (**A**) Anatomical localization of the DBS lead and active contacts. Coronal section of an anatomical MRI. Zoomed-in view of the thalamic nuclei as segmented with the CIVM atlas. DBS was delivered to either the CT (star label) or the ventral lateral thalamus (VL) (square label). 1, mediodorsal nucleus, central part; 2, mediodorsal nucleus, lateral part; 3, ventral lateral nucleus, lateral part; 4, ventral posterolateral nucleus; 5, lateral dorsal nucleus, superficial part; 6, ventral anterior nucleus, lateral part; 7, intermediodorsal nucleus; 8, mediodorsal nucleus, medial part; 9, ventral posteromedial nucleus; 10, CM nucleus, lateral part; 11, CM nucleus, medial part; 12, ventral lateral nucleus, medial part; 13, centrolateral nucleus; 14, mediodorsal nucleus, dorsal part. (**B**) Automated electrode reconstruction and three-dimensional (3D) rendering in monkey T (left), in monkey N (center), and in both monkeys (right). (**C** and **D**) Coronal histological section, corresponding to the level interaural 8.40 mm. Both cryostat image (C) and NeuN immunohistochemistry image (D) confirm the positioning of the active contacts within thalamic nuclei. (**E**) Effects of thalamic DBS on arousal in anesthetized monkeys, as a function of the electrode location and the level of stimulation (low-voltage versus high-voltage DBS). Only the stimulation of the CT could modulate arousal in the two anesthetized monkeys. (**F**) Modulation of spectral entropy (SE) by DBS. The figure consists of a distribution-smoothened version of a histogram, a box plot, and a representation of the data points. Each dot represents the average value of SE across epochs during one recording session. All pairs show significant group differences except for anesthesia + low CT-DBS and anesthesia + high VL-DBS. **0.001 < *P* ≤ 0.01, ***0.0001 < *P* ≤ 0.001, and ****P < 0.0001. *P* values are false discovery rate (FDR)–corrected.

We built an experimental setup that allowed for simultaneous fMRI acquisition, EEG recording, and DBS during finely tuned propofol anesthesia in macaques (Figs. 1 and 2, A to D, fig. S1, and table S1). We aimed at stimulating the CT as a main DBS target and the VL as a control target (Fig. 1). Following surgery to implant MRI-compatible depth electrodes, we reconstructed the implanted DBS lead and localized the anatomical sites of the four different stimulating contacts (referenced as contacts 0, 1, 2, and 3) using reconstruction methods based on both in vivo brain imaging (Fig. 2, A and B, and fig. S1) and postmortem brain histology (Fig. 2, C and D). All methods converged to the same localization of the DBS lead contacts. For monkey N, contact 0 was in the subthalamic nucleus, contact 1 in the zona incerta, contact 2 in the centromedian (CM) nucleus, and contact 3 in the VL. For monkey T, contact 0 was in the ventral posterior nucleus of the thalamus, contact 1 in the CM, contact 2 in the centrolateral nucleus of the thalamus, and contact 3 in the VL. During the DBS sessions, we stimulated the CT (contact 2 in monkey N and contact 1 in monkey T) or the VL (contact 3 in both monkeys) (Fig. 2A).

To identify the anatomical sites that were stimulated during DBS, we simulated the volume of activated tissue around the DBS lead. These simulations revealed that the affected thalamic nuclei were rather similar in the two monkeys for the two experimental DBS conditions. Compared to the low-amplitude stimulation condition, during high-amplitude CT-DBS, we affected not only the CM but also the mediodorsal and intermediodorsal thalamic nuclei. With VL-DBS, we did not affect all these nuclei simultaneously (table S1).

### Behavioral effects of thalamic DBS in anesthetized macaques

Our core hypothesis was that electrical stimulation of the CT would reverse the anesthesia-induced loss of consciousness (Fig. 2E and table S2). Therefore, we evaluated the effects of low and high DBS intensities on arousal in anesthetized macaques, outside the scanner, while the level of anesthesia was kept constant. In both monkeys (N and T), the clinical arousal score dropped from 11 to 0 when switching from wakefulness to anesthesia. Low CT-DBS increased the score to 3/11. Both animals woke up, opened their eyes spontaneously, and had spontaneous limb movements and breathing activity under high CT-DBS, thus regaining a high clinical score (9/11). In the meanwhile, under low or high VL-DBS, the clinical score remained unchanged (0/11) (Fig. 2E).

DBS significantly affected the general physiology parameters of the anesthetized monkeys such as the mean heart rate (*P* = 3.38 × 10^−26^) and mean blood pressure (*P* = 5.78 × 10^−17^) (table S2). For example, for monkey T, high CT-DBS significantly increased mean heart rate (*P* = 1.23 × 10^−23^, compared to anesthesia; *P* = 6.76 × 10^−19^, compared to low CT-DBS condition) and mean blood pressure (*P* = 8.66 × 10^−14^, compared to anesthesia condition; *P* = 6.67 × 10^−11^, compared to low CT-DBS condition).

### Thalamic DBS–induced brain activity in anesthetized macaques

**Fig. 3.**
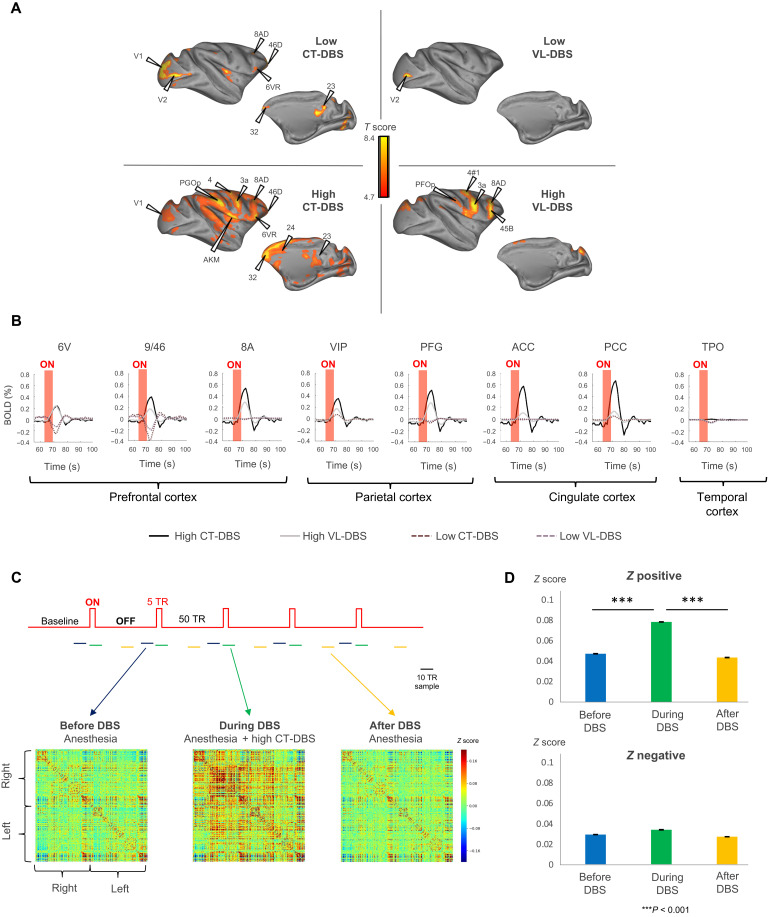
Thalamic DBS induces remote brain activations. (**A**) Effects of thalamic DBS induced on distant cortical areas. Cortical fMRI activation maps during low CT-DBS (top left), high CT-DBS (bottom left), low VL-DBS (top right), and high VL-DBS (bottom right). Individual results (monkey T), *P* < 0.05, family wise error (FWE)–corrected. (**B**) Effects of thalamic DBS on the blood oxygen level–dependent (BOLD) signal change (%) within the cortex. Red shading shows the stimulation period. High CT-DBS consistently activated a prefrontal parietal network. Only high CT-DBS activated both anterior and posterior cingulate cortices. (**C**) Effects of high CT-DBS on stationary FCs. Whole-brain average stationary intervoxel correlations before (blue), during high CT-DBS (green), and after high CT-DBS (yellow). (**D**) Average positive and negative *z* values before, during, and after high CT-DBS. In all plots, error bars represent 1 SEM. ACC, anterior cingulate cortex; area 9/46 [dorsolateral prefrontal cortex (PFCdl)]; area 8A [part of frontal eye field (FEF)]; area 6V [dorsolateral premotor cortex (PMCdl)]; area M1, primary motor cortex; PFG, parietal area PFG; VIP, intraparietal cortex (Pcip); PCC, posterior cingulate cortex; TPO, temporo-parieto-occipital–associated area in superior temporal sulcus.

We compared high CT-DBS (the only DBS condition that awakened monkeys from anesthesia) to the other conditions and found that normalized delta power was significantly lower in the high–CT-DBS condition, while normalized theta and alpha power was significantly higher (Figs. 2F and 3, figs. S2 to S5, and table S3). These changes in the spectral distribution of power were also reflected in a significantly higher median spectral frequency (MSF) and spectral entropy (SE) under the high CT-DBS condition (Fig. 2F and fig. S5).

Next, we mapped whole-brain fMRI responses to each DBS experimental condition (Fig. 3). Low CT-DBS activated localized sectors of prefrontal, parietal, anterior cingulate, temporal, and occipital cortex as well as striatum, thalamic nuclei (mediodorsal central part), midbrain, and cerebellum. The control low VL-DBS activation was restricted to the occipital cortex. High CT-DBS activated large sectors of prefrontal, parietal, anterior and posterior cingulate, temporal, occipital, and insular cortex, as well as the striatum, thalamic nuclei (central part of the mediodorsal thalamus, lateral geniculate nucleus, and reticular nucleus), hypothalamus, globus pallidus, paraseptal subpallium (Meynert basal nucleus and substantia innominata), amygdala, ventral pallium, midbrain, and cerebellum. The control high VL-DBS led to more restricted activations of prefrontal, parietal, temporal, occipital, and insular cortex, as well as striatum and cerebellum (Fig. 3, A and B, and table S3). We computed the functional correlations (FCs) during baseline (before or after stimulation) and the actual high CT-DBS period. The whole-brain distribution was significantly different between the high CT-DBS and the anesthesia baseline periods (*P* = 5.09 × 10^−3^) (Fig. 3, C and D). Among the different DBS targets and levels, only high CT-DBS activated a broad cortical and subcortical network, including cingulate cortex.

### Thalamic DBS effects on resting-state networks in anesthetized macaques

We further examined how DBS affected spontaneous fluctuations of brain activity, a fundamental mechanism that underlies states of consciousness, by computing static and dynamic resting-state FC across cortical regions.

#### Static FCs

**Fig. 4.**
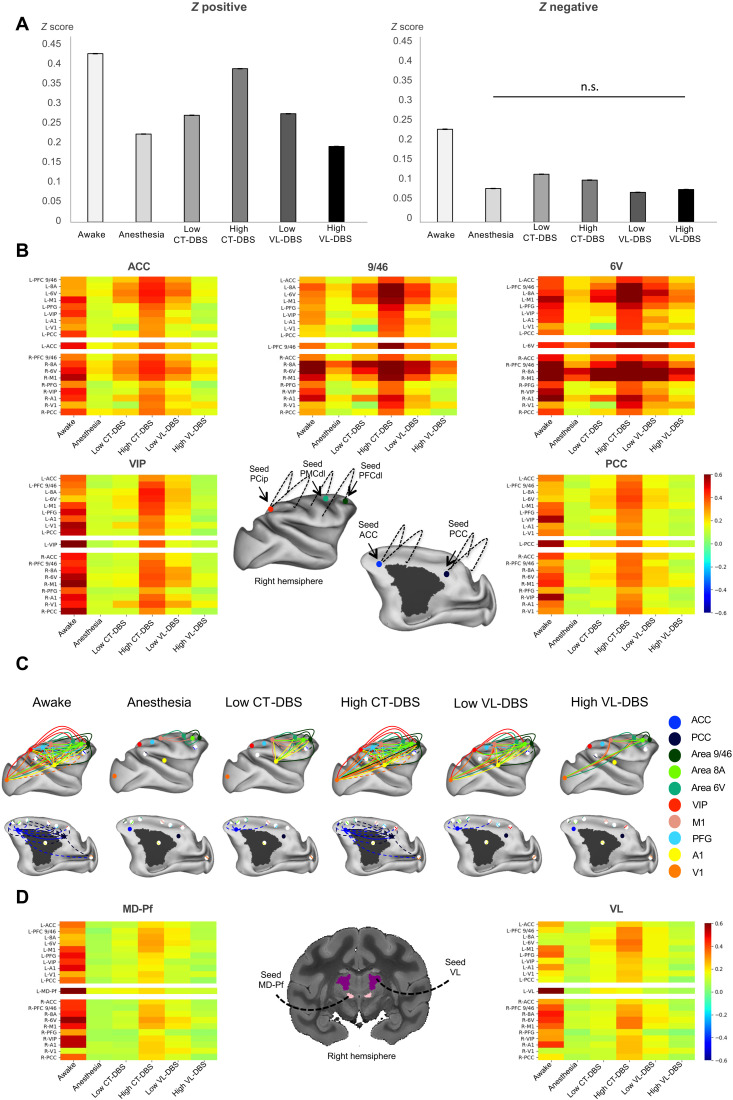
Central thalamic stimulation restores thalamocortical and corticocortical static connectivity in anesthetized macaques. (**A**) Average positive and negative *z* values within each experimental condition. In all plots, error bars represent 1 SEM. All pairwise comparisons between experimental conditions are significantly different (*P* < 0.001) both for positive and negative *z* values, except for the negative *z* value comparison between anesthesia and high VL-DBS (n.s., not significant). (**B**) Effects of DBS on corticocortical FCs. Average connectivity matrices displaying intervoxel correlations between selected cortical regions of interest (ROIs) of the “macaque” GNW (ACC; area 9/46; area 8A, area 6V, VIP, and PCC) under different experimental conditions. *x* axis, arousal state; *y* axis, studied regions. For each region, the matrix represents the FCs between the defined seed (in the right hemisphere) and the remaining selected regions. (**C**) Effects of DBS on corticocortical FCs. Schematic representation of the cortical static correlations of the macaque GNW nodes (ACC, PCC, PFCdl, FEF, PMCdl, and Pcip) and M1, S1, V1, and A1; correlation strengths higher than 0.3 of the right hemisphere for the different experimental conditions. (**D**) Effects of DBS on thalamocortical FCs. Average connectivity matrices displaying intervoxel correlations between selected thalamic nuclei and cortical ROIs representing the macaque GNW (ACC; area 9/46; area 8A, area 6V, VIP, and PCC) under different experimental conditions. *x* axis, arousal state; *y* axis, studied regions. For each region, the matrix represents the FC between the defined seed (in the right hemisphere) and the remaining studied areas. Area 9/46 (PFCdl); area 8A (part of FEF) and area 6V (PMCdl); area M1, primary motor cortex; area A1, primary auditory cortex; VIP: Pcip.

Under anesthesia, long-range bilateral intervoxel correlations largely vanished, and only intrafrontal correlations persisted (especially between areas 9/46, 8A, and 6V) (Fig. 4, fig. S6, and Supplementary Extended Results). During low CT-DBS or VL-DBS, static FC increased compared to the anesthesia condition, although *Z* score values did not reach the ones observed in the awake state. To study the specific effect of DBS on the GNW, we examined the FC within the key nodes of the “macaque GNW” as previously characterized (*46*, *50*). All GNW nodes bilaterally showed a significant correlation increase during high CT-DBS compared to anesthesia, thus recovering a pattern close to the awake state. Frontal cortex (areas 9/46, 8A, 6V, and M1) was strongly coupled to the rest of the cortical regions. The control high VL-DBS led to very low FCs across all cortical areas, although in frontal regions (areas 9/46, 8A, 6V, and M1), static FC was high and stronger within the right hemisphere compared to the left (Fig. 4, A to C, and fig. S6).

Dealing with thalamocortical FC, in the awake state, both right CM–parafascicular complex (CM-Pf) and VL showed bilateral fMRI FC with frontal, parietal, cingulate, and temporal cortices. Anesthesia abolished such FC within both hemispheres, as the correlations dropped to nonsignificant values. During low CT- or VL-DBS, correlations slightly increased compared to anesthesia, notably between the thalamus and frontal cortex. Under the crucial CT-DBS condition, bilateral connectivity between thalamic nuclei and GNW nodes was significantly enhanced compared to the anesthesia condition. Although this increase was more important with the frontal cortex (areas 9/46, 8A, 6V, and M1), it did not recover the values observed in the awake state. The control high VL-DBS presented very low FC between thalamic nuclei and cortical areas and displayed an anesthesia-like pattern (Fig. 4D).

#### Dynamic FCs

**Fig. 5.**
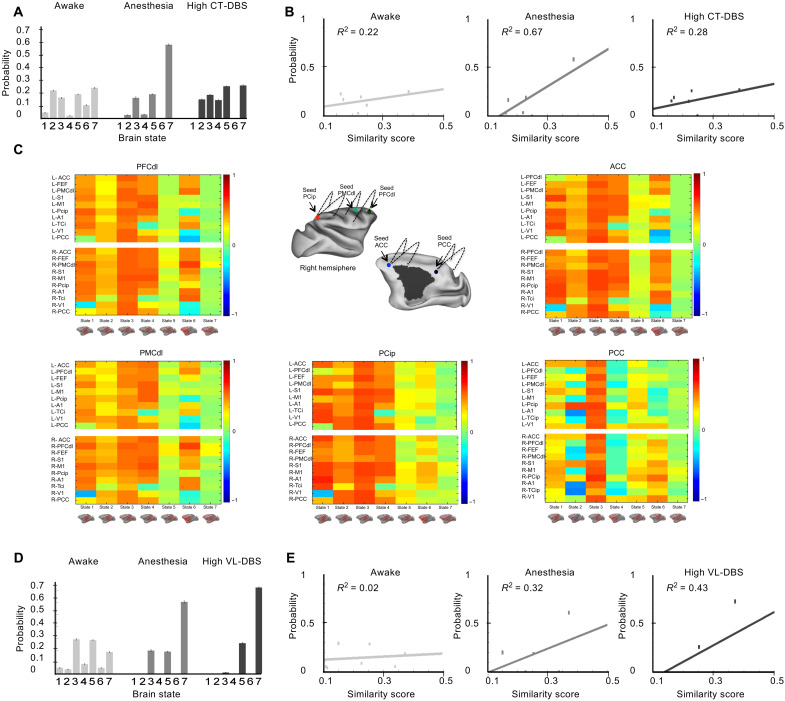
Central thalamic stimulation restores dynamic connectivity in anesthetized macaques. Effects of high CT-DBS (**A** to **C**) or high VL-DBS (**D** and **E**) on dynamic connectivity of the cortex. Unsupervised clustering of the covariance FC matrix revealed seven brain states, which are sorted according to their similarity to the structural connectivity matrix. (A) Probability distributions of brain states for the awake state, anesthesia, anesthesia + high CT-DBS clustering. Each bar represents the within-condition probability of occurrence of a state. (B) Probability of occurrence of each brain state as a function of the similarity between FC and structural connectivity for the awake state, anesthesia, and anesthesia + high CT-DBS. (C) Changes of FC across the seven brain states between the GNW and its remaining areas such as PFCdl, PMCdl, Pcip, ACC, and PCC. On the *x* axis, brain states 1 to 7. On the *y* axis, studied ROIs of the macaque GNW [ACC, PFCpol (polar prefrontal cortex), FEF, PMCdl, S1, M1, Pcip, A1, TCi, V1, and PCC of the left (L) and right (R) hemisphere] connected to the seed. (D) Probability distributions of brain states for the awake state, anesthesia, anesthesia + high VL-DBS. Each bar represents the within-condition probability of occurrence of a state. (E) Probability of occurrence of each brain state as a function of the similarity between FC and structural connectivity for the awake state, anesthesia, and anesthesia + high VL-DBS. Primary somatosensory cortex (S1); primary motor cortex (M1); primary auditory cortex (A1); inferior temporal (TCi); visual area 1 (V1).

We next assessed the dynamics of resting state by applying the unsupervised *k*-means algorithm to cluster the FC matrices of successive resting-state epochs into seven brain states, ranked according to their similarity with the structural connectivity matrix (Fig. 5, figs. S7 and S8, table S4, and Supplementary Results) (*51*). Since a rich set of brain states, independent to the structure, has been demonstrated as a signature of consciousness (*45*–*47*), we sought the same cortical functional repertoire and the underlying similarity with the anatomical connectivity during DBS. We applied *k*-means to the whole acquired dataset (including all experimental conditions) to cluster brain states. We also applied *k*-means to two data subsets, “subset CT” and “subset VL,” to specifically characterize the effects of high CT-DBS and high VL-DBS, respectively. Subset CT included data from awake, anesthesia, and anesthesia + high–CT-DBS conditions (Fig. 5, A to C). Subset VL included data from awake, anesthesia, and anesthesia + high–VL-DBS conditions (Fig. 5, D and E).

Whatever the clustered dataset (whole dataset, subset CT or subset VL) and as previously reported (*45*, *46*), we found that, in the awake state, all seven brain states were represented with a similar probability of occurrence, while under anesthesia, state 7 (with the highest function-structure similarity) was dominant and state 1 (with the lowest function-structure similarity) vanished (Fig. 5 and figs. S7 and S8). Anatomically, the functional brain states 1, 2, and 3 that were most characteristic of the awake state presented strong correlations within the macaque GNW prefrontal (dorsolateral prefrontal cortex and dorsolateral premotor cortex), parietal (intraparietal cortex), and cingulate nodes (anterior and posterior cingulate cortices), whereas state 7 displayed low or null *Z* score values across the same entire cortical network (Fig. 5C).

By clustering subset CT, we found that high CT-DBS resulted in a shift of brain state distribution with a decrease in brain state 7 probability and a richer repertoire of brain states mimicking the awake condition distribution (Fig. 5A). High CT-DBS reversed the effects of anesthesia by decreasing the function-structure similarity (Fig. 5B). Awake and high–CT-DBS slopes were significantly lower than the anesthesia slopes, indicating a greater diversity of the repertoire of brain states (awake versus anesthesia: *t* test, *P* = 6 × 10^−5^ and Bayes factor (BF)10 = 338; high CT-DBS versus anesthesia: *t* test, *P* = 0.001 and BF10 = 23). No differences were observed between awake and high CT-DBS slopes (*t* test, *P* = 0.42 and BF01 = 3.28), indicating that CT stimulation led to the full recovery of cortical dynamics. By clustering subset VL, we found that high level of stimulation of the control thalamic subregion VL (high VL-DBS) had no effect on the cortical dynamics (Fig. 5, D and E). The slopes under anesthesia were smaller than the high VL-DBS slopes (*t* test, *P* = 0.01 and BF10 = 3.81). Last, by clustering the entire dataset of resting-state fMRI, including all the experimental conditions, we replicated the same findings related to the comparison of slopes across experimental conditions, highlighting the specific effect of CT-DBS to shift cortical dynamics toward a full recovery similar to the conscious state (figs. S7 and S8).

### Effects of thalamic DBS on cortical responses to local and global novelty

**Fig. 6.**
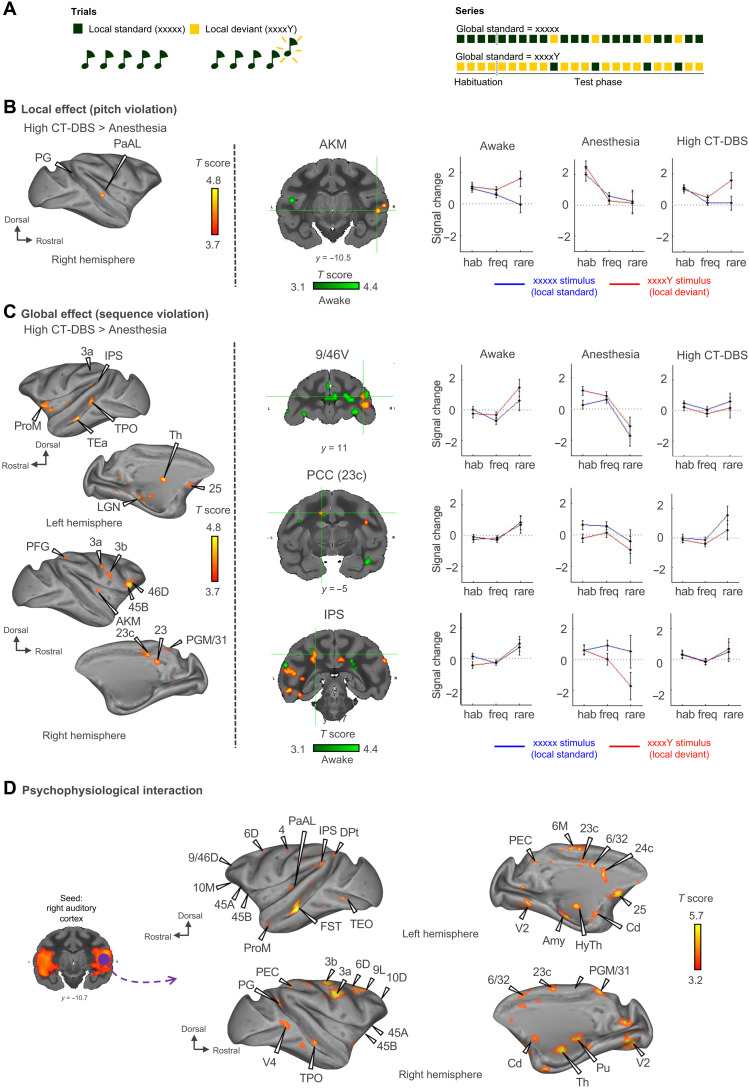
Electrical stimulation of central thalamic nuclei restores cortical hierarchical responses to local and global novelty detections in anesthetized macaques. (**A**) Experimental paradigm. The task consisted of passive listening to the local-global paradigm, which probes auditory sequence processing at two hierarchical levels of deviancy. The first level of novelty detection corresponds to a deviant sound within a sequence of identical sounds, while the second level corresponds to a deviant sequence. (**B**) First-order novelty detection “local effect” (pitch violation). Activation maps show a restoration of first-order deviancy detection when high CT-DBS is applied in anesthetized monkeys. Group results, *P* < 0.05, FDR-corrected. (**C**) Second-order novelty detection “global effect” (sequence violation). Activation maps show a partial enhancement of second-order deviancy detection during high CT-DBS in anesthetized monkeys. Group results, *P* < 0.05, FDR-corrected. fMRI signal change in area 9/46V (dorsolateral prefrontal cortex), area 23c (PCC), and IPS (intraparietal sulcus). (**D**) Task-evoked connectivity during the global novelty effect. A seed is applied to the right auditory cortex (purple) to look for PPI during the global novelty effect. High CT-DBS resulted in an increase in PPI between auditory cortex and prefrontal parietal and cingular cortices. Group results, *P* < 0.05, FDR-corrected. Area 6D (dorsal), 6M (medial), 6/32, 9L (lateral), 10D (dorsal), 10M (medial), 25, 45B, 9/46D (dorsal), PRoM (promotor), 46D (dorsal), prefrontal cortex; area 23, 23c, PGM/31, PCC; IPS, 3a, 3b (somatosensory), area PFG, PEC, PG, parietal cortex; area TEa, TPO, AKM (auditory koniocortex medial part), PaAL (para auditory lateral), FST (fundus of superior temporal sulcus) Dpt (dorsoparietal), TEO (temporo-occipital), TPO, V4 (visual area 4), temporal cortex; area V2 (visual area 2), occipital cortex; Cd, caudate nucleus; Pu, putamen; LGN, lateral geniculate nucleus; Th, thalamus; HyTh, hypothalamus.

We next investigated the effects of CT-DBS on conscious access of auditory stimuli (Fig. 6, figs. S9 to S12, and tables S5 and S6). We measured event-related fMRI responses to the local-global paradigm in which previous research demonstrated that a response to local violations (first-order novelty detection) may occur in the absence of consciousness, whereas a second-order response to global violations is a marker of conscious access (Fig. 6A and fig. S9) (*48*, *49*).

**Fig. 7.**
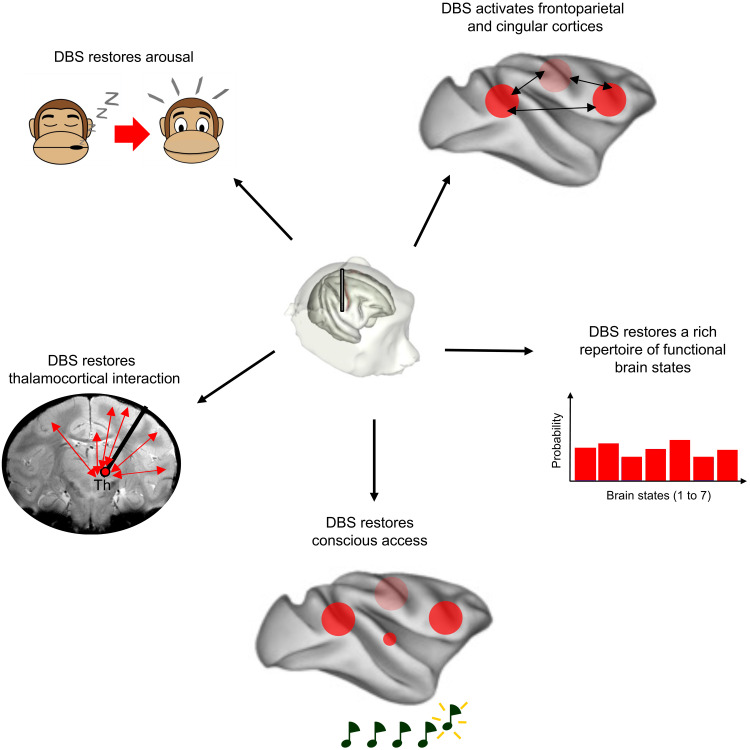
Schematic representation showing the mechanisms by which thalamic DBS restores consciousness in anesthetized macaques. Th, intralaminar nuclei of the thalamus.

Under high CT-DBS, compared to anesthesia, the effect of local violations (a contrast between local deviants and local standards) was enhanced, leading to strong activations in the left medial part of the auditory cortex, the right temporo-parieto-occipital cortex (TPO), the right temporoparietal cortex, the right parietal area PG, and the right midbrain (Fig. 6B, fig. S10, and table S5). As for the global effect (a contrast between global deviants and global standards), the contrast of high CT-DBS versus anesthesia revealed a large cortical network including the prefrontal (areas 44 and 45B), parietal (parietal area PFG and deep intraparietal area), left ventral area of visual area V4, and temporal cortex. We also found activations in the thalamus (left lateral part of the mediodorsal thalamic nucleus and left reticular thalamic nucleus) and cerebellum (Fig. 6C, figs. S11 and S12, and table S6). For both the local and global effects, the activated network elicited by the high CT-DBS is homologous to that observed previously in awake macaques (no activated regions were significantly different for the awake versus high CT-DBS comparison) (table S6) (*50*) and in healthy volunteers (*48*).

A psychophysiological interaction (PPI) analysis during high CT-DBS showed that during the global events, there was a significant increase in FC between auditory cortex and a broad cortical network that includes the prefrontal (areas 6, 8A/B, 10, and 45A/B), frontal (area 4 and premotor), parietal (areas 2 and 3a/b and dorsal intraparietal sulcus), cingulate (anterior 23c, 24c, and posterior 31), temporal (auditory cortex), occipital (areas V1 and V2), and insular cortex, as well as striatum, thalamus (ventral posterolateral nucleus and anterior pulvinar reticular nucleus), hippocampus, subpallium, nucleus accumbens, and cerebellum (Fig. 6D). Again, this cortical network elicited by the high CT-DBS is homologous to that observed previously in awake macaques (*50*).

## DISCUSSION

We demonstrated that electrical stimulation of central thalamic nuclei (CT-DBS) could recruit large-scale thalamocortical networks and restore the signatures of arousal and awareness in the nonhuman primate model of loss of consciousness (Fig. 7). While the concentration of anesthetic remained unchanged, three convergent findings indicated that CT-DBS could restore consciousness: behavioral responses, changes in resting-state fMRI dynamics, and a restoration of hierarchical cortical error detection. Those results are consistent with the hypothesis that a low function-structure similarity (*45*–*47*) and a global availability of auditory error signal (*48*) are fundamental signatures of consciousness. We previously demonstrated that all anesthetics profoundly affect cortical dynamics, with the fMRI FC maps becoming very similar to the anatomical connectivity matrix (*45*, *46*) and reproduced this finding under anesthesia while DBS was off. In a translational approach, this signature of the loss of consciousness was also found to reflect the clinical status of patients with disorders of consciousness (*47*). DBS massively altered cortical activity by shifting spontaneous fluctuations of cortical activity from a rigid brain configuration, similar to brain anatomy, to a flexible brain configuration with a rich functional repertoire of brain states, similar to the one observed in the wake state. Under high CT-DBS and same anesthesia level, the dynamic brain configurations became indistinguishable from the diverse repertoire of brain states observed in the awake state, including several states with high FCs between prefrontal, parietal, and cingular cortices.

The observed relationship between the spontaneous dynamics of global brain states and the states of consciousness converges with a recent demonstration that consciousness is characterized by a specific temporal relationship of global network transitions, notably comprising anticorrelated activations of two global coactivation patterns, respectively called the “default-mode network” and the “dorsal attention network” (*52*). In a future dedicated work, it may be possible to study the effects of causal manipulation, i.e., DBS, on such spontaneously emerging anticorrelations. Electrical stimulation of elective thalamic nuclei was previously found to modulate the arousal states of anesthetized macaques via the reconfiguration of global cortical activity and local intracolumnar integration (*31*). However, no evidence was provided that conscious access to external stimuli was modulated by thalamic stimulation. Here, we found that high CT-DBS restored the fMRI signatures of conscious access during the auditory local-global test, which probes conscious access without requiring an active report from monkeys (*18*, *48*, *50*).

In control experiments where we stimulated the VL part rather than the central part of the thalamus, we observed that DBS effects on behavior and cortical activity can be highly specific to the thalamic site of stimulation. We provide evidence from fMRI maps that VL-DBS could activate a frontoparietal network but failed to modulate the activity of the cingulate cortex, where CT-DBS activated a fronto-parieto-cingular large-scale network (Figs. 3 to 5, fig. S4, and table S3). This supports a specific role for both the anterior cingulate (*53*) and posterior cingulate (*54*) cortices in conscious processing. The striatum was also directly activated by CT-DBS and almost not by VL-DBS (table S4). This is in line with the involvement of corticostriatal activity as an important mechanism of CT-DBS to enhance arousal (*55*). Moreover, when examining the cortical dynamics at rest and its correlations with the underlying white matter, only high CT-DBS could restore a rich repertoire of brain states with a low structure-function similarity as observed in the awake macaque brain. This may explain why the effective DBS condition, i.e., high CT-DBS, restored not only arousal but also conscious access to external auditory stimuli. Although no event-related fMRI data were acquired with the local-global paradigm under VL-DBS, which is a potential limitation of our study, VL-DBS had no effect on consciousness level, as observed with behavioral score, nor on cortical dynamics as measured with resting-state fMRI. In the local-global test, second-order sequence violations (global effect) also activate a fronto-parieto-cingular network, both in healthy volunteers (*48*) and in awake macaques (*50*).

A potential biological explanation for the broad effects of DBS relates to the great heterogeneity of the neural circuits that surround the DBS lead and which were presumably recruited by DBS in a nonspecific manner. In Parkinson’s disease, DBS is thought to yield a therapeutic benefit by spreading beyond the targeted basal ganglia neurons and affecting many of the afferent axons of the stimulated region (*56*) and cortical dynamics (*57*). In our study, the DBS leads targeted the central thalamic nuclei. Stimulation, however, had the effect of not only modulating the CM neurons but also a broad thalamocortical circuit. Thus, thalamic DBS achieves a double cortical input through corticothalamic axon stimulation (retrograde mechanism) and thalamocortical axon stimulation (anterograde mechanisms). This bidirectional modulation of thalamocortical pathways might be key to modulate both arousal and awareness. Using optogenetics, Liu and colleagues (*40*) modulated CT neurons in anesthetized rats and demonstrated a restoration of arousal during optical monodirectional thalamocortical modulation. Gent and colleagues (*58*) demonstrated that optogenetic stimulation of centromedial thalamus could induce a transition from non–rapid eye movement sleep to wakefulness in mice through broad cortical activity modulation. The same group also demonstrated the key role of direct monosynaptic transmission between hypothalamus and thalamus to cause transition across states of consciousness (*59*). These biological mechanisms may participate in explaining our findings. DBS could also modulate axons from the ascending reticular activating system that project massively into the intralaminar nuclei of the thalamus (*60*). Unlike the fine-grained optogenetic experiments that can be performed in rodents (*11*, *13*), the present experiments are limited by an inherent uncertainty surrounding the exact site of stimulation with DBS. This limit could be addressed in future work, as more precise electrophysiological and optogenetic tools are increasingly becoming available in nonhuman primates.

Our results also shed light on the fundamental mechanisms of consciousness and of anesthesia-induced loss of consciousness. Anesthesia-induced loss of consciousness is thought to be related to the suppression of cortical feedback while preserving some degree of feedforward cortical activity (*32*, *33*). A recent study demonstrated this decoupling at the cellular level (*11*). If this thalamic mechanism is fundamental to the loss of consciousness in anesthesia, then the observed wakefulness restoration by thalamic stimulation in anesthetized macaques would be associated with a restoration of feedback corticocortical activity. This has been demonstrated recently using layer-specific recordings in the macaque cortex (*31*). In our experiment, we stimulated two thalamic subregions, the CT and the VL nucleus. While CT-DBS awakened anesthetized macaques and restored conscious access, VL-DBS did not induce any arousal effect. Rather, VL-DBS increased the structure-function brain similarity, potentially increasing the depth of anesthesia. This selective effect is in line with the findings of Suzuki and Larkum (*11*). In their experiments, only the inhibition of higher-order thalamic nuclei could abolish the capability to process sensory stimuli, which persisted when low-order thalamic nuclei (such as ventral posteromedial thalamus) were inhibited. At the brain scale, our findings are consistent with the hypothesis that anesthetics suppress consciousness by disrupting long-distance corticocortical and corticothalamic networks (*17*, *61*).

From a theoretical point of view, our findings fit with several theories of consciousness. The restoration of a global availability of auditory error signals by CT-DBS is in line with the TCL hypothesis, which stresses the need for an interplay between two thalamocortical systems (specific sensory thalamic nuclei and intralaminar nonspecific nuclei) and the corresponding cortical areas to achieve conscious experience (*2*). The observed responses also fit with the GNW hypothesis, which stipulates that what we experience as awareness is a consequence of the global availability of a specific piece of information to a broad cortical network, made possible by long-distance corticocortical connectivity and corresponding thalamocortical loops (*3*). Last, CT-DBS induced a shift in spontaneous cortical dynamics, which is line with both simulations of the GNW (*6*) and, more broadly, with the dynamic core hypothesis in the IIT theory (*14*). The central role of thalamocortical connectivity for conscious states is also a key feature of other theories of consciousness (*9*, *11*–*13*).

The present experiments were also designed as a potential step in the development of a treatment for patients with chronic disorders of consciousness. Our findings suggest that DBS of CT might help restore not only arousal but also awareness in patients with disorders of consciousness, as previously investigated in some patients (*37*, *43*). While previous studies demonstrated that direct modulation of other cortical (*62*) and subcortical (*63*–*65*) brain areas could reverse the anesthesia-induced loss of consciousness in rodent models, there was no evidence that it could also restore conscious access. Nevertheless, a clear limitation of our experiment is that it relies on a primate model of loss of consciousness due to anesthetic agents (*18*, *45*, *46*). In this model, the anatomical structures of the midbrain reticular formation, thalamus, and cortex are entirely intact and allow DBS to modulate whole-brain activity. Contrariwise, patients with disorders of consciousness generally exhibit severe structural brain injuries (*20*) that may make it challenging to decide whether these patients have sufficient structural integrity within the thalamocortical loops to make thalamic DBS effective.

While this is an obvious limitation of our model, we note that any attempt to model more closely patients with disorders of consciousness by performing large-scale cortical and subcortical lesions in animals would be ethically very challenging and perhaps unjustifiable. Instead, we suggest that a clinical trial could be designed for a subset of patients in long-lasting minimally conscious state, unresponsive to pharmacological agents, and in whom anatomical and diffusion MRI shows a preservation of thalamocortical pathways and fMRI or metabolic evidence of a partially preserved thalamo-prefronto-parieto-cingular network. Such patients would undergo a meticulous evaluation with clinical testing, structural MRI, and functional neuroimaging (*66*, *67*) before receiving implantations targeting the CT. Future translational directions also include the perspective of noninvasive thalamic modulation in patients with disorders of consciousness, for instance, using focused ultrasound stimulation (*68*), which will require additional technological developments and clinical investigations.

## MATERIALS AND METHODS

Study design, experimental steps, and modalities of investigation are summarized in Fig. 1.

### Animals

All procedures were conducted in accordance with the European convention for animal care (86-406) and the National Institutes of Health’s Guide for the Care and Use of Laboratory Animals. Animal studies were approved by the Institutional Ethical Committee (CETEA protocols #12-086 and #16-040). Five male rhesus macaques (*Macaca mulatta*), 9 to 17 years and 7.5 to 9.1 kg, were included, three for the awake (non-DBS) experiments (monkeys B, J, and Y) and two for the DBS experiments (monkeys N and T). Previously published awake data from three rhesus macaques (*50*) were used for comparison for the local/global paradigm (Fig. 6 and tables S5 and S6).

### Surgical procedures and electrode location

#### Headpost implantation

For the awake resting-state experiments, monkeys B, J, and Y were implanted with an MR-compatible headpost under general anesthesia (*45*, *50*).

#### DBS electrode implantation

Monkeys N and T were implanted with a clinical DBS electrode (Medtronic, Minneapolis, MN, USA, lead model 3389). The DBS lead had four active contacts for electrical stimulation (1.5-mm contact length, 0.5-mm spacing, and 1.27-mm diameter). We performed stereotactic surgery, targeting the right CM thalamus using a neuronavigation system (BrainSight, Rogue, Canada), guided by the rhesus macaque atlases (*69*, *70*) and a preoperative and intraoperative anatomical MRI [MPRAGE (magnetization prepared - rapid gradient echo), T1-weighted, repetition time (TR) = 2200 ms, inversion time (TI) = 900 ms, 0.80-mm isotropic voxel size, and sagittal orientation]. The electrode was stabilized with the Stimloc lead anchoring device (Medtronic, Minneapolis, MN, USA). The extracranial part of the DBS lead was hosted using a homemade three-dimensional (3D) printed MRI-compatible chamber. We waited at least 20 days after implantation before starting the DBS-fMRI experiments (for more details, see the Supplementary Materials).

#### Anatomical localization of the DBS lead

We used two methods to ensure for the anatomical localization of the DBS lead and the DBS contacts. First, we used a reconstruction method based on in vivo brain imaging (Fig. 2, A and B, and fig. S1). Second, we conducted a histology study in one of the implanted monkeys (Fig. 2, Cand D). We simulated the volume of activated tissue for all the DBS conditions using the modeling module available in the Lead-DBS toolbox and reported the thalamic nuclei activated (minimum 40% whole size) for each DBS condition (table S1).

##### DBS lead localization using MRI

We used the Lead-DBS macaque toolbox (www.lead-dbs.org/lead-dbs-optimized-to-support-macaque-imaging-data/) (MATLAB, MathWorks, MA) (*71*) based on pre- and postoperative anatomical MRI to localize the implanted DBS electrode. Preoperative images were acquired on the surgery day. Postoperative images were acquired 2 weeks later. To control for the stability of the electrode position during the experimental period, additional postoperative images were acquired at least 4 months after DBS implantation.

In MRI processing, pre- and postoperative structural MRIs were preprocessed using Pypreclin (*72*) and aligned to the Montreal Neurological Institute (MNI) macaque brain template (*73*). The 3D T1 images underwent three consecutive processing steps consisting of (i) reorientation to match the atlas space orientation (right anterosuperior) from the original sphinx position (*74*), (ii) B1 field correction to correct for low-frequency intensity nonuniformities (*75*), and (iii) masking plus normalization to exclude the extracranial tissues by projecting the mask of the template back to the native space and to align the anatomical data into the MNI macaque brain template using both affine and nonlinear transforms. The images used as inputs for the Lead-DBS macaque toolbox thus corresponded to preprocessed pre- and postoperative volumes of 0.8-mm isotropic resolution sharing the exact same orientation, matrix size, and number of voxels, free of MRI artifact and aligned with the MNI macaque space.

In electrode trajectory reconstruction, the DBS electrode model (Medtronic 3389) was specified in the Lead-DBS macaque module to automatically retrieve the lead measurements of the manufacturer and reconstruct the anatomical trajectory, creating a vector through the entire brain volume. We manually defined the entry point and the target for the prereconstruction.

In contact localization, the 3D coordinates of each of the four contacts were retrieved from the Lead-DBS macaque toolbox and accurately localized in the MNI macaque brain space (*x*, *y*, *z*). We confirmed the labeling of the targeted nuclei with the Paxinos (L, B, and S) (*69*), Saleem (*70*), and CIVM (*76*) reference atlases to provide a better parcellation of thalamic and basal ganglia nuclei (fig. S1). We also performed an additional check of contact localization using the electrode-induced artifact on the structural MRI. To this end, we used MRI measurements of the contact artifact in vivo as previously described (*77*). This method uses the measurements of maximal height and width of the artifacts induced by proximal and distal contacts of the DBS electrode to compute their coordinates and deduce the others ones given the geometry of the lead (contact size and interdistance).

##### DBS lead localization using histology

We used a reconstruction method based on postmortem brain histology (Fig. 2, C and D). Following all experiments, one of the two implanted monkeys was euthanized using intravenous pentobarbital (60 mg/kg) and perfused transcardially with cold saline and 4% paraformaldehyde. The brain was immediately removed and immersed in a cold 4% paraformaldehyde solution and later on sectioned in a coronal plane on a sliding freezing cryostat (40-μm-thick sections). For immunohistochemical labeling, sections were first incubated in phosphate-buffered saline containing 1% Triton X-100, 1% bovine serum albumin, 5% normal serum, and the appropriate dilution of the primary antibody anti-NeuN (1:1000, Chemicon, CA). We acquired images of sections using an optical microscope (Leica Microsystems IR GmbH, Germany). Confocal images were captured using the Zeiss Zen 2 blue edition software. More details were described previously (*78*, *79*).

### Behavioral assessment

We used a clinical arousal scale adapted from (*18*) to characterize the monkey behavior in the awake state, under anesthesia and DBS (low CT-DBS, high CT-DBS, low VL-DBS, and high VL-DBS). The clinical arousal scale is based on the exploration of the surrounding world (0, absence; 1, small search of external clue; 2, total investigation of the environment-like head orientation to a sound), spontaneous movements (0, absence; 1, small torso and/or limb movement; 2, large torso and/or limb movement), shaking/prodding (0, nothing; 1, small body movement; 2, large body movement), toe pinch (0, nothing; 1, body movement or eye blinking or cardiac rate change; 2, body movement and eye blinking or eye opening and cardiac rate change), eye opening (0, nothing; 1, small blinks or eye movements; 2, full eye opening), and corneal reflex (0, absent; 1, present). Behavioral assessment was performed outside the scanner in the awake state, under anesthesia, and under each DBS condition when the animal was not paralyzed (Fig. 2B).

### fMRI data acquisition

Monkeys were scanned on a 3-T horizontal scanner (Siemens Prisma Fit, Erlanger, Germany) with a customized eight-channel phased-array surface coil (KU Leuven, Belgium). The parameters of the MRI sequences were as follows: (i) block design and resting-state experiments: echo planar imaging (EPI), TR = 1250 ms, echo time (TE) = 14.20 ms, 1.25-mm isotropic voxel size, and 325 and 500 brain volumes per run, respectively; (ii) auditory task-evoked experiments: EPI, TR = 2400 ms, TE = 19.40 ms, 1.00-mm isotropic voxel size, and 111 brain volumes per run. Monocrystalline iron oxide nanoparticles (10 mg/kg, i.v.; MION, Feraheme, AMAG Pharmaceuticals, MA) were injected into the monkey’s saphenous vein (*74*) before the auditory task-evoked scanning session; (iii) anatomical scan: MPRAGE, T1-weighted, TR = 2200 ms, TI = 900 ms, 0.80-mm isotropic voxel size, and sagittal orientation.

#### Awake protocol

We acquired fMRI in awake macaques as previously described (*45*, *50*). Animals were trained to sit in a sphinx position in a primate chair with their head fixed, without any task, and the eye position was monitored at 120 Hz (Iscan Inc., MA, USA).

#### Anesthesia protocol for the DBS experiments

Anesthesia was induced with an intramuscular injection of ketamine (10 mg/kg; Virbac, France) and dexmedetomidine (20 μg/kg; Ovion Pharma, USA) and maintained with a target-controlled infusion (TCI) (Alaris PK Syringe pump, CareFusion, CA, USA) of propofol (Panpharma Fresenius Kabi, France) using the “Paedfusor” pharmacokinetic model (monkey T: TCI, 4.6 to 4.8 μg/ml; monkey N: TCI, 4.0 to 4.2 μg/ml) (*80*). Monkeys were intubated and mechanically ventilated (Aestiva/5 MRI, General Electrics Healthcare, USA). The physiology parameters (heart rate, noninvasive blood pressure, oxygen saturation, respiratory rate, end-tidal carbon dioxide, and cutaneous temperature) were monitored (Maglife, Schiller, France) (table S2). A muscle-blocking agent (cisatracurium, 0.15 mg/kg, bolus i.v., followed by continuous intravenous infusion at a rate of 0.18 mg/kg per hour; GlaxoSmithKline, France) was used during all anesthesia fMRI sessions to avoid artifacts. The level of sedation was defined by a clinical score and continuous EEG using an MR-compatible EEG system combining a custom-built 13-channel EEG cap (EasyCap), an amplifier (BrainAmp, Brain Products), and the Vision Recorder software (Brain Products) (*18*, *45*, *46*).

### Electroencephalography

We acquired scalp EEG using an MR-compatible system and custom-built caps (EasyCap, 13 channels), an MR amplifier (BrainAmp, Brain Products, Germany), and the Vision Recorder software (Brain Products), as previously described (*18*, *45*, *46*). Parameters were as follows: sampling rate, 5000 per channel, common reference electrode, impedance of <20 megohms; band-pass filtered, 0.01 Hz < *f* < 500 Hz during collection. We applied an EEG gel to obtain low impedances (One Step EEG gel, Germany).

The scanner artifacts were corrected using the BrainVision Analyzer 2.1.2.327 software by first averaging the EEG epochs affected by the MR scanner and then subtracting this average template. For artifact detection, we used the gradient method and the automatic detection of scanner episodes. For detection, all available channels with a signal were used (Fp1, Fp2, F3, F4, T3, T4, P3, P4, O1, Oz, and O2). The artifact type was set to continuous, meaning that the detection was done on artifacts that follow one another without interruption, with a TR value of 1250 ms (offset set to 10 ms). The gradient trigger was set to 200 μV/ms. Baseline correction was computed over the whole artifact (0 to 200 ms). Sliding average calculation was enabled with 21 intervals used for the calculation of the correction template. The correction was performed on the same aforementioned channels (fig. S2).

Following the MR artifact cleaning, the signal was filtered between 1 Hz (IIR Butterworth high-pass zero-phase two-pass forward and reverse noncausal filter, order 12—effective, after forward-backward) and 25 Hz (IIR Butterworth lowpass zero-phase noncausal filter, order 16—effective, after forward-backward) and downsampled to 250-Hz sampling rate. The data were cut 15 s after the start of the stimulation until 15 s before the end of the scanning. The remaining time series was cut into epochs of 0.8 s, with a random jitter ranging from 0.55 to 0.85 s. For the cleaning of artifacted epochs or channels, the Python package Autoreject (*81*, *82*) was used with the number of channel interpolations equal to 1, 2, 4, or 8. Average EEG reference projection was applied. All of the preprocessing following MR artifact removal was done in Python using the MNE-Python (*83*) and Autoreject packages.

To analyze the differences between the five conditions in this study (anesthesia and 3 or 5 V of DBS in both CT and VL), we calculated EEG markers that were previously reported to distinguish between patients with and without disorders of consciousness (*84*, *85*). As previously explained (*84*), we used markers belonging to the group of spectral measures. These markers are the normalized spectral power of delta (1 to 4 Hz), theta (4 to 8 Hz), and alpha (8 to 13 Hz) oscillatory bands; the SE, which is a measure of signal predictability; and the median power frequency (MSF), which is the frequency that divides the power spectrum into two equal areas. The markers were calculated using the package NICE-tools (*85*). The details regarding their calculations were described previously (*84*).

The SciPy implementation of the Mann-Whitney *U* two-sided test was used to investigate the differences between two groups with independent data samples (*86*). To control for the type 1 error rate, the false discovery rate (FDR)–corrected *P* values were analyzed. The Benjamini-Hochberg FDR correction for independent or positively correlated tests was used. The minimal significance level, alpha, was set to 0.05 for all statistical tests. The raincloud plots were obtained using the Python package PtitPrince (*87*).

### Statistical analysis of the physiological parameters

The statistical analysis of the physiological parameters was performed with an analysis of variance (ANOVA) and multiple comparison using homemade MATLAB scripts (MathWorks, USA). The mean heart rate and blood pressure were tested for each subject with a statistical threshold of *P* < 0.001, Bonferroni-corrected.

### Electrical stimulation protocol for the DBS experiments

The DBS electrode was plugged to an external stimulator (DS8000, World Precision Instrument, USA), and all the parameters were tuned to a fixed value of frequency (*f* = 130.208 Hz, *T* = 7.68 ms), waveform (monopolar signal), and length of width pulse (monkey N, *w* = 320 μs; monkey T, *w* = 140 μs). The absolute voltage amplitude was set to 3 V (“low” DBS) or 5 V (“high” DBS).

#### DBS block design fMRI

The DBS block design experiments were acquired under anesthesia with a low or high DBS on either the CT or the VL thalamic nucleus lead to assess FCs (Fig. 3). The block design pattern was designed first with a rest period, free of any electrical stimulation (“baseline,” 50 TR), followed by five alternations of a DBS period (“DBS ON,” 5 TR) on one of the contact leads at either low or high DBS, followed each time by a rest period where the DBS was switched off (“DBS OFF,” 50 TR). The total duration of the DBS block design had 325 repetitions (Fig. 3).

#### Resting-state fMRI

The resting-state fMRI experiments were acquired either in the awake state or under anesthesia without or with a low or high DBS during the entire run on either the CT or VL thalamic nuclei (Figs. 4 and 5). The DBS started a few seconds before the beginning of the fMRI sequence and stopped just after the end of the MRI sequence.

#### Event-related auditory task fMRI: Local/global paradigm

The event-related auditory task experiments were acquired under anesthesia without any DBS or with a high CT-DBS during the entire run (Fig. 6). No data were acquired under VL-DBS. Data previously acquired in the awake state (*50*) were used for comparison.

The local-global paradigm is based on local deviants (within trials) and global deviants (across trials) of temporal regularities (*48*, *50*). Each trial is made of five consecutive sounds (either a high-pitch 1600 Hz or a low-pitch 800 Hz, 50-ms duration, 150-ms stimulus onset asynchrony between sounds, and a total duration of 650 ms). The series of sounds are separated by 850-ms interstimulus interval, for a total trial duration of 1500 ms (fig. S7). The trials were presented first with a rest period (6 TR) followed by five series of 24 trials (15 TR), each followed by a rest period (6 TR). Each 24-trial series comprises an initial series of 4 habituation trials, followed by 20 posthabituation trials with four global deviants and 16 global standards. The total duration was 111 TR. Auditory stimuli were presented with the E-prime software (E-Studio 1.0, Psychology Software Tools) and delivered with MR-compatible earphones at a level of 80 dB.

### fMRI preprocessing

Images were preprocessed using Pypreclin (Python preclinical pipeline) (*72*). Functional images were corrected for slice timing and B_0_ inhomogeneities, reoriented, realigned, resampled (1.0 mm isotropic), masked, coregistered to the MNI macaque brain template (*73*), and smoothed (3.0-mm Gaussian kernel). Anatomical images were corrected for B_1_ inhomogeneities, normalized to the anatomical MNI macaque brain template, and masked. The CIVM atlas [template, labels, 241 regions of interest (ROIs)] (*76*) was warped to the MNI macaque space (*73*) to recover brain region names and localize clusters. FSLeyes tool (https://zenodo.org/record/2630502; FSLeyes version 0.28.0) was used to show brain slices. Whole-brain data were displayed using Caret software (version 5.61, http://brainvis.wustl.edu/wiki/index.php/Caret:About).

### fMRI statistical analysis

#### Block design fMRI analysis

We assessed the consistency of the block design for all DBS experimental conditions (low CT-DBS, high CT-DBS, low VL-DBS, and high VL-DBS) using a finite impulse response (FIR) model, without a priori, performed with the Nistat Python module (*88*). We first performed a statistical analysis of the cerebral areas activated during the block design experiments using an 11th-order FIR model of 5 TR from the stimulation onset to cover the design as a whole. The first-level analysis was performed using a generalized linear model and consisted of the convolution of the stimulus categories (during and immediate DBS and after DBS) with the implemented blood oxygen level–dependent (BOLD) hemodynamic response function and its time derivative. We also added motion regressors as variables of noninterest. Activation time series of all the fMRI voxels were computed for each fMRI run and signal change expressed in *T* score maps for the different stimulation time relative to rest periods. For the second-level analysis, we included the β-weight images from the first-level analysis for each monkey and each fMRI block and computed a second-level whole-brain ANOVA. We defined the DBS ON versus DBS OFF effect by contrasting the fMRI activations to DBS ON and poststimulation 1 to 5 TR relative to rest. Each second-level contrast was computed at a statistical threshold of *P* < 0.001, uncorrected at the voxel level, and *P* < 0.05, family wise error (FWE)–corrected at the cluster level. We reported the activated regions of at least three contiguous voxels (table S3).

#### Resting-state fMRI analysis

##### Static resting state

For the whole-brain average static intervoxel correlations before, during high CT-DBS, and after high CT-DBS of the block design experiment, we extracted the fMRI signal from 222 cortical and subcortical brain regions. Regions were defined by the CIVM template (*76*), revised into a revised CIVM atlas (table S7) to match the fMRI resolution, and aligned to the MNI macaque brain template (*73*). The FCs before (10 TR before the electrical stimulation onset), during high CT-DBS (10 TR, 5 TR stimulation delivery and 5 TR of immediate poststimulation), and after high CT-DBS (10 TR, spaced 25 TR after the stimulation onset) were displayed as a matrix where the *x* and *y* axes contained all the 222 ROIs.

For the static resting state, we extracted the fMRI signal from 222 ROIs defined using the revised CIVM atlas (*76*). We selected 10 cortical and 2 thalamic ROIs, relevant for the macaque GNW [anterior cingulate cortex (ACC); area 9/46, prefrontal cortex; area 8A, part of frontal eye field; area 6V, premotor cortex; area M1, primary motor cortex; PFG, parietal cortex; area A1, primary auditory cortex; ventral intraparietal sulcus (VIP); posterior cingulate cortex (PCC); and CM-Pf and VL thalamic nuclei] displayed in Figs. 4 and 5 with a schematic representation of these correlations with an absolute correlation strength higher than 0.3. The results of the interregion ANOVA comparisons are displayed as *P* value matrices (fig. S6).

##### Dynamic resting state

Detailed methodology of the analysis of the dynamic resting-state fMRI was previously described (*45*). In summary, an anatomical connectivity matrix was derived from the CoCoMac 2.0 database (*51*), which is based on a vast summary of axonal tract tracing studies using a regional map parcellation comprising 82 cortical ROIs (*89*). This anatomical connectivity matrix was compared to the “functional connectivity” matrices obtained from our fMRI data. Static and sliding-window zero-lag covariance matrices were estimated for each arousal/anesthetic condition c and session s (*90*). For the dynamic correlation matrices, we estimated sliding-window Fisher-transformed covariance matrices *Z*_c,s,w_ for each arousal condition c, session s, and time window w (*91*). Covariance matrices from windowed segments of the time series were computed, with a Hamming window (width = 35 scans), sliding with steps of one scan, resulting in 464 windows (w) per session. For each condition c and session s, we got a 3D matrix *C*_c,s,w_ sized 82 by 82 by 464, which was Fisher-transformed (*Z*_c,s,w_) before further analysis.

We then determined the dominant recurrent patterns of brain correlations (brain states) by means of an unsupervised clustering method along the time dimension of the *Z*_c,s,w_ matrix. To evaluate the structure of reoccurring correlation patterns, we applied the *k*-means clustering algorithm to *Z*_c,s,w_ matrices using the L1 distance function (Manhattan distance), as implemented in MATLAB (MathWorks) (*92*). This analysis was done by pooling together the data from different experimental conditions [awake state + anesthesia + high CT-DBS (Fig. 5, A to C); awake state + anesthesia + high VL-DBS (Fig. 5, D to F); and awake state + anesthesia + low CT-DBS + high CT-DBS + low VL-DBS + high VL-DBS (figs. S7 and S8)] and mixed to avoid any bias.

The number of brain states was determined with a predefined number of brain states *n* to 7. The method gives, for each point in time, the most likely state of FCs. This permits to compare how these states and their dynamics change with the level of arousal or the anesthetic agent that was administrated to the animal.

To explore the dependence of brain dynamics and arousal condition, a measure of similarity between anatomical connectivity (CoCoMac 2.0) and FC was defined, to class all brain states along this dimension. Normalized probability distribution of *z* values for brain states 1 and 7, binning correlation values in 45 bins, and 2D normalized histograms of the same brain state *z* values, as a function of distance between pairs of ROIs, are represented in fig. S6. The distance between ROIs was calculated as the L2 norm in the 3D space, using MNI coordinates of CoCoMac as input. We also calculated the duration of each brain state (the average length of sequences of a given brain state in the *B*_c,s,w_ matrix) (fig. S8).

We performed Bayesian statistics on the slope and the probability of brain state 7 (the one more similar to the anatomical connectivity) corresponding to each recording session and compared the distributions under the awake, anesthesia, and DBS conditions. We computed the Bayes factors BF10 (giving the evidence for H1 over H0) and BF01 (evidence for H0 over H1). BF > 3 was interpreted as a strong evidence for the tested hypothesis (table S4).

#### Event-related task fMRI analysis

We used the SPM toolbox (SPM5 software, Wellcome Department of Cognitive Neurology, London, UK; MATLAB, MathWorks, USA) to determine the responses to the local-global paradigm. For the second-level analysis, the β-weight images from the first-level analysis were included to compute a whole-brain ANOVA. We defined three different contrasts: activation to all sounds relative to rest, the local effect (local deviants contrasted to local standards), and the global effect (rare trials minus frequent trials). We also compared the whole-brain results of the different experimental conditions (anesthesia versus high CT-DBS and anesthesia versus high CT-DBS versus the awake state). We used a statistical threshold of *P* < 0.05, FDR-corrected at the cluster level for the group analysis, and *P* < 0.001 for the individual analysis.

The PPI analyses were performed with the dedicated tool on SPM5 (SPM5 software, Wellcome Department of Cognitive Neurology, London, UK; MATLAB, MathWorks, USA) (*93*). The global novelty effect on the FC between the right auditory cortex and the rest of the brain was investigated by extracting the residual first-level model that was added as a new regressor of interest in the first- and second-level analysis.

## Notes

View/request a protocol for this paper from *Bio-protocol*.

## References

[1] C. M. Signorelli, J. Szczotka, R. Prentner, “Explanatory profiles of models of consciousness- towards a systematic classification” (PsyArXiv, 2021);https://osf.io/f5vdu. 10.1093/nc/niab021 PMC8396118 34457353

[2] R. Llinás, U. Ribary, D. Contreras, C. Pedroarena, The neuronal basis for consciousness. Philos. Trans. R. Soc. Lond. B Biol. Sci. 353, 1841–1849 (1998). 9854256 10.1098/rstb.1998.0336 PMC1692417

[3] S. Dehaene, J.-P. Changeux, Experimental and theoretical approaches to conscious processing. Neuron 70, 200–227 (2011). 21521609 10.1016/j.neuron.2011.03.018

[4] S. Dehaene, M. Kerszberg, J. P. Changeux, A neuronal model of a global workspace in effortful cognitive tasks. Proc. Natl. Acad. Sci. U.S.A. 95, 14529–14534 (1998). 9826734 10.1073/pnas.95.24.14529 PMC24407

[5] B. J. Baars, *A Cognitive Theory of Consciousness* (Cambridge Univ. Press, 1988).

[6] S. Dehaene, J.-P. Changeux, Ongoing spontaneous activity controls access to consciousness: A neuronal model for inattentional blindness. PLoS Biol. 3, e141 (2005). 15819609 10.1371/journal.pbio.0030141 PMC1074751

[7] S. Dehaene, C. Sergent, J.-P. Changeux, A neuronal network model linking subjective reports and objective physiological data during conscious perception. Proc. Natl. Acad. Sci. U.S.A. 100, 8520–8525 (2003). 12829797 10.1073/pnas.1332574100 PMC166261

[8] Z. Gao, C. Davis, A. M. Thomas, M. N. Economo, A. M. Abrego, K. Svoboda, C. I. De Zeeuw, N. Li, A cortico-cerebellar loop for motor planning. Nature 563, 113–116 (2018). 30333626 10.1038/s41586-018-0633-x PMC6212318

[9] Z. V. Guo, H. K. Inagaki, K. Daie, S. Druckmann, C. R. Gerfen, K. Svoboda, Maintenance of persistent activity in a frontal thalamocortical loop. Nature 545, 181–186 (2017). 28467817 10.1038/nature22324 PMC6431254

[10] G. A. Mashour, P. Roelfsema, J.-P. Changeux, S. Dehaene, Conscious processing and the global neuronal workspace hypothesis. Neuron 105, 776–798 (2020). 32135090 10.1016/j.neuron.2020.01.026 PMC8770991

[11] M. Suzuki, M. E. Larkum, General anesthesia decouples cortical pyramidal neurons. Cell 180, 666–676.e13 (2020). 32084339 10.1016/j.cell.2020.01.024

[12] J. Aru, M. Suzuki, M. E. Larkum, Cellular mechanisms of conscious processing. Trends Cogn. Sci. 24, 814–825 (2020). 32855048 10.1016/j.tics.2020.07.006

[13] S. Manita, T. Suzuki, C. Homma, T. Matsumoto, M. Odagawa, K. Yamada, K. Ota, C. Matsubara, A. Inutsuka, M. Sato, M. Ohkura, A. Yamanaka, Y. Yanagawa, J. Nakai, Y. Hayashi, M. E. Larkum, M. Murayama, A top-down cortical circuit for accurate sensory perception. Neuron 86, 1304–1316 (2015). 26004915 10.1016/j.neuron.2015.05.006

[14] G. Tononi, G. M. Edelman, Consciousness and complexity. Science 282, 1846–1851 (1998). 9836628 10.1126/science.282.5395.1846

[15] S. Vijayan, S. Ching, P. L. Purdon, E. N. Brown, N. J. Kopell, Thalamocortical mechanisms for the anteriorization of alpha rhythms during propofol-induced unconsciousness. J. Neurosci. 33, 11070–11075 (2013). 23825412 10.1523/JNEUROSCI.5670-12.2013 PMC3718379

[16] F. J. Flores, K. E. Hartnack, A. B. Fath, S.-E. Kim, M. A. Wilson, E. N. Brown, P. L. Purdon, Thalamocortical synchronization during induction and emergence from propofol-induced unconsciousness. Proc. Natl. Acad. Sci. U.S.A. 114, E6660–E6668 (2017). 28743752 10.1073/pnas.1700148114 PMC5558998

[17] L. J. Velly, M. F. Rey, N. J. Bruder, F. A. Gouvitsos, T. Witjas, J. M. Regis, J. C. Peragut, F. M. Gouin, Differential dynamic of action on cortical and subcortical structures of anesthetic agents during induction of anesthesia. Anesthesiology 107, 202–212 (2007). 17667563 10.1097/01.anes.0000270734.99298.b4

[18] L. Uhrig, D. Janssen, S. Dehaene, B. Jarraya, Cerebral responses to local and global auditory novelty under general anesthesia. Neuroimage 141, 326–340 (2016). 27502046 10.1016/j.neuroimage.2016.08.004 PMC5635967

[19] G. Tononi, An information integration theory of consciousness. BMC Neurosci. 5, 42 (2004). 15522121 10.1186/1471-2202-5-42 PMC543470

[20] N. D. Schiff, Central thalamic contributions to arousal regulation and neurological disorders of consciousness. Ann. N. Y. Acad. Sci. 1129, 105–118 (2008). 18591473 10.1196/annals.1417.029

[21] G. Moruzzi, H. W. Magoun, Brain stem reticular formation and activation of the EEG. Electroencephalogr. Clin. Neurophysiol. 1, 455–473 (1949). 18421835

[22] M. Steriade, L. L. Glenn, Neocortical and caudate projections of intralaminar thalamic neurons and their synaptic excitation from midbrain reticular core. J. Neurophysiol. 48, 352–371 (1982). 6288887 10.1152/jn.1982.48.2.352

[23] B. A. Vogt, P. R. Hof, D. P. Friedman, R. W. Sikes, L. J. Vogt, Norepinephrinergic afferents and cytology of the macaque monkey midline, mediodorsal, and intralaminar thalamic nuclei. Brain Struct. Funct. 212, 465–479 (2008). 18317800 10.1007/s00429-008-0178-0 PMC2649766

[24] S. Heckers, C. Geula, M. M. Mesulam, Cholinergic innervation of the human thalamus: Dual origin and differential nuclear distribution. J. Comp. Neurol. 325, 68–82 (1992). 1282919 10.1002/cne.903250107

[25] O. Akeju, M. L. Loggia, C. Catana, K. J. Pavone, R. Vazquez, J. Rhee, V. Contreras Ramirez, D. B. Chonde, D. Izquierdo-Garcia, G. Arabasz, S. Hsu, K. Habeeb, J. M. Hooker, V. Napadow, E. N. Brown, P. L. Purdon, Disruption of thalamic functional connectivity is a neural correlate of dexmedetomidine-induced unconsciousness. eLife 3, e04499 (2014). 25432022 10.7554/eLife.04499 PMC4280551

[26] M. T. Alkire, J. R. McReynolds, E. L. Hahn, A. N. Trivedi, Thalamic microinjection of nicotine reverses sevoflurane-induced loss of righting reflex in the rat. Anesthesiology 107, 264–272 (2007). 17667571 10.1097/01.anes.0000270741.33766.24

[27] M. T. Alkire, C. D. Asher, A. M. Franciscus, E. L. Hahn, Thalamic microinfusion of antibody to a voltage-gated potassium channel restores consciousness during anesthesia. Anesthesiology 110, 766–773 (2009). 19322942 10.1097/aln.0b013e31819c461c

[28] M. I. Lioudyno, A. M. Birch, B. S. Tanaka, Y. Sokolov, A. L. Goldin, K. G. Chandy, J. E. Hall, M. T. Alkire, Shaker-related potassium channels in the central medial nucleus of the thalamus are important molecular targets for arousal suppression by volatile general anesthetics. J. Neurosci 33, 16310–16322 (2013). 24107962 10.1523/JNEUROSCI.0344-13.2013 PMC3792466

[29] J. L. Baker, J.-W. Ryou, X. F. Wei, C. R. Butson, N. D. Schiff, K. P. Purpura, Robust modulation of arousal regulation, performance, and frontostriatal activity through central thalamic deep brain stimulation in healthy nonhuman primates. J. Neurophysiol. 116, 2383–2404 (2016). 27582298 10.1152/jn.01129.2015 PMC5116485

[30] A. M. Bastos, J. A. Donoghue, S. L. Brincat, M. Mahnke, J. Yanar, J. Correa, A. S. Waite, M. Lundqvist, J. Roy, E. N. Brown, E. K. Miller, Neural effects of propofol-induced unconsciousness and its reversal using thalamic stimulation. eLife 10, e60824 (2021). 33904411 10.7554/eLife.60824 PMC8079153

[31] M. J. Redinbaugh, J. M. Phillips, N. A. Kambi, S. Mohanta, S. Andryk, G. L. Dooley, M. Afrasiabi, A. Raz, Y. B. Saalmann, Thalamus modulates consciousness via layer-specific control of cortex. Neuron 106, 66–75.e12 (2020). 32053769 10.1016/j.neuron.2020.01.005 PMC7243351

[32] U. Lee, S. Ku, G. Noh, S. Baek, B. Choi, G. A. Mashour, Disruption of frontal-parietal communication by ketamine, propofol, and sevoflurane. Anesthesiology 118, 1264–1275 (2013). 23695090 10.1097/ALN.0b013e31829103f5 PMC4346246

[33] G. A. Mashour, Top-down mechanisms of anesthetic-induced unconsciousness. Front. Syst. Neurosci. 8, 115 (2014). 25002838 10.3389/fnsys.2014.00115 PMC4066704

[34] M. Corazzol, G. Lio, A. Lefevre, G. Deiana, L. Tell, N. André-Obadia, P. Bourdillon, M. Guenot, M. Desmurget, J. Luauté, A. Sirigu, Restoring consciousness with vagus nerve stimulation. Curr. Biol. 27, R994–R996 (2017). 28950091 10.1016/j.cub.2017.07.060

[35] B. Hermann, F. Raimondo, L. Hirsch, Y. Huang, M. Denis-Valente, P. Pérez, D. Engemann, F. Faugeras, N. Weiss, S. Demeret, B. Rohaut, L. C. Parra, J. D. Sitt, L. Naccache, Combined behavioral and electrophysiological evidence for a direct cortical effect of prefrontal tDCS on disorders of consciousness. Sci. Rep. 10, 4323 (2020). 32152347 10.1038/s41598-020-61180-2 PMC7062738

[36] A. Thibaut, N. Schiff, J. Giacino, S. Laureys, O. Gosseries, Therapeutic interventions in patients with prolonged disorders of consciousness. Lancet Neurol. 18, 600–614 (2019). 31003899 10.1016/S1474-4422(19)30031-6

[37] P. Bourdillon, B. Hermann, J. D. Sitt, L. Naccache, Electromagnetic brain stimulation in patients with disorders of consciousness. Front. Neurosci. 13, 223 (2019). 30936822 10.3389/fnins.2019.00223 PMC6432925

[38] S. Laureys, M. E. Faymonville, A. Luxen, M. Lamy, G. Franck, P. Maquet, Restoration of thalamocortical connectivity after recovery from persistent vegetative state. Lancet 355, 1790–1791 (2000). 10832834 10.1016/s0140-6736(00)02271-6

[39] M. L. Kringelbach, N. Jenkinson, S. L. F. Owen, T. Z. Aziz, Translational principles of deep brain stimulation. Nat. Rev. Neurosci. 8, 623–635 (2007). 17637800 10.1038/nrn2196

[40] J. Liu, H. J. Lee, A. J. Weitz, Z. Fang, P. Lin, M. Choy, R. Fisher, V. Pinskiy, A. Tolpygo, P. Mitra, N. Schiff, J. H. Lee, Frequency-selective control of cortical and subcortical networks by central thalamus. eLife 4, e09215 (2015). 26652162 10.7554/eLife.09215 PMC4721962

[41] Y. Abe, T. Tsurugizawa, D. L. Bihan, Water diffusion closely reveals neural activity status in rat brain loci affected by anesthesia. PLOS Biol. 15, e2001494 (2017). 28406906 10.1371/journal.pbio.2001494 PMC5390968

[42] F. Cohadon, E. Richer, Deep cerebral stimulation in patients with post-traumatic vegetative state. 25 cases. Neurochirurgie 39, 281–292 (1993). 8065486

[43] N. D. Schiff, J. T. Giacino, K. Kalmar, J. D. Victor, K. Baker, M. Gerber, B. Fritz, B. Eisenberg, T. Biondi, J. O’Connor, E. J. Kobylarz, S. Farris, A. Machado, C. McCagg, F. Plum, J. J. Fins, A. R. Rezai, Behavioural improvements with thalamic stimulation after severe traumatic brain injury. Nature 448, 600–603 (2007). 17671503 10.1038/nature06041

[44] J. Lemaire, A. Sontheimer, B. Pereira, J. Coste, S. Rosenberg, C. Sarret, G. Coll, J. Gabrillargues, B. Jean, T. Gillart, A. Coste, B. Roche, A. Kelly, B. Pontier, F. Feschet, Deep brain stimulation in five patients with severe disorders of consciousness. Ann. Clin. Transl. Neurol. 5, 1372–1384 (2018). 30480031 10.1002/acn3.648 PMC6243378

[45] P. Barttfeld, L. Uhrig, J. D. Sitt, M. Sigman, B. Jarraya, S. Dehaene, Signature of consciousness in the dynamics of resting-state brain activity. Proc. Natl. Acad. Sci. U.S.A. 112, 887–892 (2015). 25561541 10.1073/pnas.1418031112 PMC4311826

[46] L. Uhrig, J. D. Sitt, A. Jacob, J. Tasserie, P. Barttfeld, M. Dupont, S. Dehaene, B. Jarraya, Resting-state dynamics as a cortical signature of anesthesia in monkeys. Anesthesiology 129, 942–958 (2018). 30028727 10.1097/ALN.0000000000002336

[47] A. Demertzi, E. Tagliazucchi, S. Dehaene, G. Deco, P. Barttfeld, F. Raimondo, C. Martial, D. Fernández-Espejo, B. Rohaut, H. U. Voss, N. D. Schiff, A. M. Owen, S. Laureys, L. Naccache, J. D. Sitt, Human consciousness is supported by dynamic complex patterns of brain signal coordination. Sci. Adv. 5, eaat7603 (2019). 30775433 10.1126/sciadv.aat7603 PMC6365115

[48] T. A. Bekinschtein, S. Dehaene, B. Rohaut, F. Tadel, L. Cohen, L. Naccache, Neural signature of the conscious processing of auditory regularities. Proc. Natl. Acad. Sci. U.S.A. 106, 1672–1677 (2009). 19164526 10.1073/pnas.0809667106 PMC2635770

[49] F. Faugeras, B. Rohaut, N. Weiss, T. A. Bekinschtein, D. Galanaud, L. Puybasset, F. Bolgert, C. Sergent, L. Cohen, S. Dehaene, L. Naccache, Probing consciousness with event-related potentials in the vegetative state. Neurology 77, 264–268 (2011). 21593438 10.1212/WNL.0b013e3182217ee8 PMC3136052

[50] L. Uhrig, S. Dehaene, B. Jarraya, A hierarchy of responses to auditory regularities in the macaque brain. J. Neurosci. 34, 1127–1132 (2014). 24453305 10.1523/JNEUROSCI.3165-13.2014 PMC5635960

[51] R. Bakker, T. Wachtler, M. Diesmann, CoCoMac 2.0 and the future of tract-tracing databases. Front. Neuroinform. 6, 30 (2012). 23293600 10.3389/fninf.2012.00030 PMC3530798

[52] Z. Huang, J. Zhang, J. Wu, G. A. Mashour, A. G. Hudetz, Temporal circuit of macroscale dynamic brain activity supports human consciousness. Sci. Adv. 6, eaaz0087 (2020). 32195349 10.1126/sciadv.aaz0087 PMC7065875

[53] O. Raccah, N. Block, K. C. R. Fox, Does the prefrontal cortex play an essential role in consciousness? Insights from intracranial electrical stimulation of the human brain. J. Neurosci. 41, 2076–2087 (2021). 33692142 10.1523/JNEUROSCI.1141-20.2020 PMC8018764

[54] J. Annen, L. Heine, E. Ziegler, G. Frasso, M. Bahri, C. Di Perri, J. Stender, C. Martial, S. Wannez, K. D’ostilio, E. Amico, G. Antonopoulos, C. Bernard, F. Tshibanda, R. Hustinx, S. Laureys, Function-structure connectivity in patients with severe brain injury as measured by MRI-DWI and FDG-PET. Hum. Brain Mapp. 37, 3707–3720 (2016). 27273334 10.1002/hbm.23269 PMC6867513

[55] M. Afrasiabi, M. J. Redinbaugh, J. M. Phillips, N. A. Kambi, S. Mohanta, A. Raz, A. M. Haun, Y. B. Saalmann, Consciousness depends on integration between parietal cortex, striatum, and thalamus. Cell Syst. 12, 363–373.e11 (2021). 33730543 10.1016/j.cels.2021.02.003 PMC8084606

[56] V. Gradinaru, M. Mogri, K. R. Thompson, J. M. Henderson, K. Deisseroth, Optical deconstruction of parkinsonian neural circuitry. Science 324, 354–359 (2009). 19299587 10.1126/science.1167093 PMC6744370

[57] C. de Hemptinne, N. C. Swann, J. L. Ostrem, E. S. Ryapolova-Webb, M. San Luciano, N. B. Galifianakis, P. A. Starr, Therapeutic deep brain stimulation reduces cortical phase-amplitude coupling in Parkinson’s disease. Nat. Neurosci. 18, 779–786 (2015). 25867121 10.1038/nn.3997 PMC4414895

[58] T. C. Gent, M. Bandarabadi, C. G. Herrera, A. R. Adamantidis, Thalamic dual control of sleep and wakefulness. Nat. Neurosci. 21, 974–984 (2018). 29892048 10.1038/s41593-018-0164-7 PMC6438460

[59] C. G. Herrera, M. C. Cadavieco, S. Jego, A. Ponomarenko, T. Korotkova, A. Adamantidis, Hypothalamic feedforward inhibition of thalamocortical network controls arousal and consciousness. Nat. Neurosci. 19, 290–298 (2016). 26691833 10.1038/nn.4209 PMC5818272

[60] J. Parvizi, A. Damasio, Consciousness and the brainstem. Cognition 79, 135–160 (2001). 11164026 10.1016/s0010-0277(00)00127-x

[61] M. M. Monti, E. S. Lutkenhoff, M. Rubinov, P. Boveroux, A. Vanhaudenhuyse, O. Gosseries, M.-A. Bruno, Q. Noirhomme, M. Boly, S. Laureys, Dynamic change of global and local information processing in propofol-induced loss and recovery of consciousness. PLoS Comput. Biol. 9, e1003271 (2013). 24146606 10.1371/journal.pcbi.1003271 PMC3798283

[62] D. Pal, J. G. Dean, T. Liu, D. Li, C. J. Watson, A. G. Hudetz, G. A. Mashour, Differential role of prefrontal and parietal cortices in controlling level of consciousness. Curr. Biol. 28, 2145–2152.e5 (2018). 29937348 10.1016/j.cub.2018.05.025 PMC6039257

[63] N. E. Taylor, C. J. Van Dort, J. D. Kenny, J. Pei, J. A. Guidera, K. Y. Vlasov, J. T. Lee, E. S. Boyden, E. N. Brown, K. Solt, Optogenetic activation of dopamine neurons in the ventral tegmental area induces reanimation from general anesthesia. Proc. Natl. Acad. Sci. U.S.A. 113, 12826–12831 (2016). 27791160 10.1073/pnas.1614340113 PMC5111696

[64] S. Gao, A. Proekt, N. Renier, D. P. Calderon, D. W. Pfaff, Activating an anterior nucleus gigantocellularis subpopulation triggers emergence from pharmacologically-induced coma in rodents. Nat. Commun. 10, 2897 (2019). 31263107 10.1038/s41467-019-10797-7 PMC6603023

[65] L. Wang, W. Zhang, Y. Wu, Y. Gao, N. Sun, H. Ding, J. Ren, L. Yu, L. Wang, F. Yang, W. Xi, M. Yan, Cholinergic-induced specific oscillations in the medial prefrontal cortex to reverse propofol anesthesia. Front. Neurosci. 15, 664410 (2021). 34121993 10.3389/fnins.2021.664410 PMC8187623

[66] S. Laureys, J. T. Giacino, N. D. Schiff, M. Schabus, A. M. Owen, How should functional imaging of patients with disorders of consciousness contribute to their clinical rehabilitation needs? Curr. Opin. Neurol. 19, 520–527 (2006). 17102688 10.1097/WCO.0b013e3280106ba9 PMC2858870

[67] J. Vanhoecke, M. Hariz, Deep brain stimulation for disorders of consciousness: Systematic review of cases and ethics. Brain Stimul. 10, 1013–1023 (2017). 28966051 10.1016/j.brs.2017.08.006

[68] M. M. Monti, C. Schnakers, A. S. Korb, A. Bystritsky, P. M. Vespa, Non-invasive ultrasonic thalamic stimulation in disorders of consciousness after severe brain injury: A first-in-man report. Brain Stimul. 9, 940–941 (2016). 27567470 10.1016/j.brs.2016.07.008

[69] G. Paxinos, X.-F. Huang, M. Petrides, A. W. Toga, *The Rhesus Monkey Brain in Stereotaxic Coordinates* (Academic Press, 2008).

[70] K. S. Saleem, N. K. Logothetis, *A Combined MRI and Histology Atlas of the Rhesus Monkey Brain in Stereotaxic Coordinates* (Academic Press Inc, ed. 2, 2012).

[71] A. Horn, N. Li, T. A. Dembek, A. Kappel, C. Boulay, S. Ewert, A. Tietze, A. Husch, T. Perera, W.-J. Neumann, M. Reisert, H. Si, R. Oostenveld, C. Rorden, F.-C. Yeh, Q. Fang, T. M. Herrington, J. Vorwerk, A. A. Kühn, Lead-DBS v2: Towards a comprehensive pipeline for deep brain stimulation imaging. Neuroimage 184, 293–316 (2019). 30179717 10.1016/j.neuroimage.2018.08.068 PMC6286150

[72] J. Tasserie, A. Grigis, L. Uhrig, M. Dupont, A. Amadon, B. Jarraya, Pypreclin: An automatic pipeline for macaque functional MRI preprocessing. Neuroimage 207, 116353 (2020). 31743789 10.1016/j.neuroimage.2019.116353

[73] S. Frey, D. N. Pandya, M. M. Chakravarty, L. Bailey, M. Petrides, D. L. Collins, An MRI based average macaque monkey stereotaxic atlas and space (MNI monkey space). Neuroimage 55, 1435–1442 (2011). 21256229 10.1016/j.neuroimage.2011.01.040

[74] W. Vanduffel, D. Fize, J. B. Mandeville, K. Nelissen, P. Van Hecke, B. R. Rosen, R. B. Tootell, G. A. Orban, Visual motion processing investigated using contrast agent-enhanced fMRI in awake behaving monkeys. Neuron 32, 565–577 (2001). 11719199 10.1016/s0896-6273(01)00502-5

[75] J. P. Marques, T. Kober, G. Krueger, W. van der Zwaag, P.-F. Van de Moortele, R. Gruetter, MP2RAGE, a self bias-field corrected sequence for improved segmentation and T1-mapping at high field. Neuroimage 49, 1271–1281 (2010). 19819338 10.1016/j.neuroimage.2009.10.002

[76] E. Calabrese, A. Badea, C. L. Coe, G. R. Lubach, Y. Shi, M. A. Styner, G. A. Johnson, A diffusion tensor MRI atlas of the postmortem rhesus macaque brain. Neuroimage 117, 408–416 (2015). 26037056 10.1016/j.neuroimage.2015.05.072 PMC4512905

[77] C. Pollo, J.-G. Villemure, F. Vingerhoets, J. Ghika, P. Maeder, R. Meuli, Magnetic resonance artifact induced by the electrode Activa 3389: An in vitro and in vivo study. Acta Neurochir. 146, 161–164 (2004). 14963749 10.1007/s00701-003-0181-4

[78] X. Drouot, S. Oshino, B. Jarraya, L. Besret, H. Kishima, P. Remy, J. Dauguet, J. P. Lefaucheur, F. Dollé, F. Condé, M. Bottlaender, M. Peschanski, Y. Kéravel, P. Hantraye, S. Palfi, Functional recovery in a primate model of Parkinson’s disease following motor cortex stimulation. Neuron 44, 769–778 (2004). 15572109 10.1016/j.neuron.2004.11.023

[79] B. Jarraya, S. Boulet, G. S. Ralph, C. Jan, G. Bonvento, M. Azzouz, J. E. Miskin, M. Shin, T. Delzescaux, X. Drouot, A.-S. Hérard, D. M. Day, E. Brouillet, S. M. Kingsman, P. Hantraye, K. A. Mitrophanous, N. D. Mazarakis, S. Palfi, Dopamine gene therapy for Parkinson’s disease in a nonhuman primate without associated dyskinesia. Sci. Transl. Med. 1, 2ra4 (2009). 10.1126/scitranslmed.3000130 20368163

[80] A. Absalom, G. Kenny, ‘Paedfusor’ pharmacokinetic data set. Br. J. Anaesth. 95, 110–110 (2005). 10.1093/bja/aei567 15941735

[81] M. Jas, D. Engemann, F. Raimondo, Y. Bekhti, A. Gramfort, Automated rejection and repair of bad trials in MEG/EEG, in *Proceedings of the 2016 International Workshop Pattern Recognition in Neuroimaging PRNI*, Trento, Italy, 22 to 24 June 2016.

[82] M. Jas, D. A. Engemann, Y. Bekhti, F. Raimondo, A. Gramfort, Autoreject: Automated artifact rejection for MEG and EEG data. Neuroimage 159, 417–429 (2017). 28645840 10.1016/j.neuroimage.2017.06.030 PMC7243972

[83] A. Gramfort, MEG and EEG data analysis with MNE-Python. Front. Neurosci. 7, 267 (2013). 24431986 10.3389/fnins.2013.00267 PMC3872725

[84] J. D. Sitt, J.-R. King, I. El Karoui, B. Rohaut, F. Faugeras, A. Gramfort, L. Cohen, M. Sigman, S. Dehaene, L. Naccache, Large scale screening of neural signatures of consciousness in patients in a vegetative or minimally conscious state. Brain 137, 2258–2270 (2014). 24919971 10.1093/brain/awu141 PMC4610185

[85] D. A. Engemann, F. Raimondo, J.-R. King, B. Rohaut, G. Louppe, F. Faugeras, J. Annen, H. Cassol, O. Gosseries, D. Fernandez-Slezak, S. Laureys, L. Naccache, S. Dehaene, J. D. Sitt, Robust EEG-based cross-site and cross-protocol classification of states of consciousness. Brain 141, 3179–3192 (2018). 30285102 10.1093/brain/awy251

[86] P. Virtanen, R. Gommers, T. E. Oliphant, M. Haberland, T. Reddy, D. Cournapeau, E. Burovski, P. Peterson, W. Weckesser, J. Bright, S. J. van der Walt, M. Brett, J. Wilson, K. J. Millman, N. Mayorov, A. R. J. Nelson, E. Jones, R. Kern, E. Larson, C. J. Carey, İ. Polat, Y. Feng, E. W. Moore, J. VanderPlas, D. Laxalde, J. Perktold, R. Cimrman, I. Henriksen, E. A. Quintero, C. R. Harris, A. M. Archibald, A. H. Ribeiro, F. Pedregosa, P. van Mulbregt; SciPy 1.0 Contributors, SciPy 1.0: Fundamental algorithms for scientific computing in Python. Nat. Methods 17, 261–272 (2020). 32015543 10.1038/s41592-019-0686-2 PMC7056644

[87] M. Allen, D. Poggiali, K. Whitaker, T. R. Marshall, J. van Langen, R. A. Kievit, Raincloud plots: A multi-platform tool for robust data visualization. Wellcome Open Res. 4, 63 (2019). 31069261 10.12688/wellcomeopenres.15191.1 PMC6480976

[88] A. Abraham, F. Pedregosa, M. Eickenberg, P. Gervais, A. Mueller, J. Kossaifi, A. Gramfort, B. Thirion, G. Varoquaux, Machine learning for neuroimaging with scikit-learn. Front. Neuroinform. 8, 14 (2014). 24600388 10.3389/fninf.2014.00014 PMC3930868

[89] R. Kötter, E. Wanke, Mapping brains without coordinates. Philos. Trans. R. Soc. Lond. B Biol. Sci. 360, 751–766 (2005). 15971361 10.1098/rstb.2005.1625 PMC1569487

[90] E. A. Allen, E. Damaraju, S. M. Plis, E. B. Erhardt, T. Eichele, V. D. Calhoun, Tracking whole-brain connectivity dynamics in the resting state. Cereb. Cortex 24, 663–676 (2014). 23146964 10.1093/cercor/bhs352 PMC3920766

[91] R. M. Hutchison, T. Womelsdorf, J. S. Gati, S. Everling, R. S. Menon, Resting-state networks show dynamic functional connectivity in awake humans and anesthetized macaques. Hum. Brain Mapp. 34, 2154–2177 (2013). 22438275 10.1002/hbm.22058 PMC6870538

[92] S. Lloyd, Least squares quantization in PCM. IEEE Trans. Inf. Theory 28, 129–137 (1982).

[93] K. J. Friston, C. Buechel, G. R. Fink, J. Morris, E. Rolls, R. J. Dolan, Psychophysiological and modulatory interactions in neuroimaging. Neuroimage 6, 218–229 (1997). 9344826 10.1006/nimg.1997.0291

