# Supplementary material for: Deep brain stimulation of the thalamus restores signatures of consciousness in a nonhuman primate model
Source: Sci Adv. 2022 Mar 18;8(11):eabl5547. doi: 10.1126/sciadv.abl5547 (PMC8932660; doi:10.1126/sciadv.abl5547)
Supplement: Supplementary file 1 — Supplementary Text Figs. S1 to S12 Tables S1 to S7 [file sciadv.abl5547_sm.pdf]

Science Advances
AAAS

Supplementary Materials for

Deep brain stimulation of the thalamus restores signatures of consciousness in a nonhuman primate model

Jordy Tasserie, Lynn Uhrig, Jacobo D. Sitt, Dragana Manasova, Morgan Dupont, Stanislas Dehaene, Béchir Jarraya*

*Corresponding author. Email: bechir.jarraya@cea.fr

Published 18 March 2022, *Sci. Adv.* **8**, eabl5547 (2022)
DOI: 10.1126/sciadv.abl5547

This PDF file includes:

Supplementary Text
Figs. S1 to S12
Tables S1 to S7

Supplementary Text

-Extended Materials and Methods

Deep brain stimulation (DBS) methodology

Surgery using neuro-navigation

We implanted a four-lead (0, 1, 2, 3) MRI-compatible clinical DBS electrode (Medtronic 3389, USA) in two macaques (monkey N and T) with the ultimate goal of performing simultaneous DBS-fMRI acquisitions. Electrode leads were 1.5 mm long and spaced by 0.5 mm. The external diameter was 1.27 mm. We targeted the right centro-median thalamus (CM) and performed aseptic stereotaxic surgery under general anesthesia using a neuro-navigation system (BrainSight, Rogue, Canada) guided by anatomical 3T MRI (Prisma Fit, Siemens, Germany) (MPRAGE, T1 weighted, repetition time TR = 2200ms; inversion time TI=900ms, 0.80mm isotropic voxel size, sagittal orientation, mono-channel 1Tx-1Rx circular surface coil of 12.5 cm diameter). The head of the monkey was placed in a stereotaxic frame and maintained with ears and ocular bars. All devices were built in plastic and MR compatible materials. Gadolinium fiducials were placed on the frame, as well as on the macaque skull and the temporary headpost that held additional fiducials. These landmarks, recognized by the neuro-navigation system, were put all around the skull in a non-coplanar manner. Pre-operative MRI images aimed at defining the target and trajectory. We assessed the target location and trajectory according to the MNI macaque brain coordinates (x, y, z) and Paxinos Atlas reference space (L, B, S). We confirmed the contact spot following three other approaches: i) anterior-posterior commissure (AC-PC) system; ii) distance to different anatomical area landmarks such as the right caudate nucleus and iii) Saleem Atlas. Lead placement trajectory was simulated with the neuro-navigation module prior to surgery.

We drilled craniotomy, positioned a plastic cannula to guide the DBS electrode and fixed an anchoring device system (Stim-lock, Medtronic, USA) covering the craniotomy. This element aimed at stabilizing and blocking the lead extremity to avoid lead migration. Per-operative MRI were acquired to control convergence between the theoretical and the practical implantation spot (effective target reached during surgery versus desired planned location). The extracranial part of the lead was

protected with a plastic MR compatible chamber that was home-made by 3D-printing and was fixed to the skull with screws and dental acrylic.

Verification of the DBS settings and underlying behavioral responses

To ensure efficiency across experiments, impedances between leads and through electrode to an external reference were first measured outside the MRI environment with the DBS programmer device provided by the manufacturer (8840 N'Vision, Activa Clinician DBS Programmer, Medtronic, USA). Ultimately, we used an oscilloscope (Wave Runner 44XI, LeCroy, USA) to check the electrical current delivered to each lead at the beginning and at the end of each experimental session. The stimulation also generated an artifact on the EEG signal, which provided a final benchmark during the fMRI acquisitions.

Behavioral responses (see behavioral assessment section) to electrical thalamic stimulation were assessed in each animal outside the scanner at least 20 days after the DBS implantation. We empirically explored different voltage amplitudes and pulse widths while keeping a monopolar stimulation at a frequency of 130Hz, applied successively to each of the four DBS contacts. The DBS lead targeted the centro-median thalamus. On the target contact (centered in CM), we determined the voltage level for high central thalamic (CT) DBS as the voltage just above the threshold at which a significant behavioral response was observed. Low CT-DBS corresponded to a lower current delivery below the voltage level that led to an arousal pattern. For comparison and reproducibility purposes, we kept the exact same DBS settings for the control stimulation site (ventro-lateral thalamus (VL) DBS).

fMRI statistical analysis

Block-design fMRI analysis

We generated plots by extracting the activations responses to high CT-DBS with the hemodynamic function in frontal (area 6V; 9/46; 8A), parietal (ventral intraparietal VIP, parietal area PFG), cingulate (anterior ACC: posterior PCC) and temporal cortex (temporo-parieto-occipital area TPO). Activity profiles were plotted as percentage of signal changes across time.

Resting-state fMRI analysis

For the static resting state, we calculated for each experimental condition and sessions the average of positive and negative Z-values and performed a Student t-test with the null hypothesis of zero correlation to test for statistical significance of connectivity between the different experimental conditions.

We computed the static functional correlations by estimating for each experimental condition (noted *e*) for the awake state, anesthesia, low CT-DBS, high CT-DBS, low VL-DBS and high VL-DBS and acquired run (noted *r*) the covariance matrix $C_{e,r}$. This value was obtained by extracting and averaging across all runs *r* the time series of all voxels included in each selected anatomical ROI. We referred as static functional correlation or stationary functional connectivity the entry matrix $C_{e,r}$ (i,j) where each cell represented the mean strength of the functional correlation between the i-j pair. For each covariance matrix, a Fisher transformation was applied to calculate the Z-score. The Z-score matrices were averaged across runs to obtain one matrix per experimental condition. To assess statistical significance, Student t-test at the threshold p value<0.0001 and a false discovery rate correction were applied on the correlations of all pairs of brain regions.

Dynamic resting state fMRI analysis

Covariance values between all ROIs were included ([82×(82−1)]/2=3,321 features per matrix). $Z_{c,s,w}$ matrices were subsampled along the time dimension (*w*) before clustering. The resulting centroids or median clusters (BS_n with n=1–7; each BS_n is sized 82×82) were then used to initialize a clustering of all data, obtaining a matrix of brain states $B_{c,s,w}$, which, for a given arousal condition c and session s, is a vector of length 464, valued 1–7, because each matrix in $Z_{s,p,w}$ is assigned a BS_n.

The similarity score was computed from the correlation coefficient between the vectorized structural matrix and each vectorized brain state from the clustering analysis. All brain states were ordered in ascending order of similarity to the structure using the similarity score. To quantify the relation between the probability of occurrence of a BS and the similarity score, for each arousal condition, a regression analysis was done, to quantify the beta value (β), the R^2 and a P value. The differences in BS composition across arousal states was evaluated through a fixed-effects ANOVA, with mean rank similarity, that is, the result of averaging each BS time series, valued from 1 to 7, as a dependent

variable and the vigilance condition as the in-dependent variable. A fixed-effects ANOVA was run to quantify the effect of sedation on the probability of brain state 7. For this, we followed the same procedure, but the mean rank similarity was calculated considering only BS 7 (window *w* valued at 1; any other state window *w* valued at 0).

To explore specifically the fluctuations of intervoxel correlation within nodes of the "macaque global neuronal worskspace" and sensori-motor areas (anterior cingulate cortex, ACC; dorsolateral prefrontal cortex, PFCdl; frontal eye fields, FEF; dorsolateral premotor cortex, PMCdl; primary somatosensory cortex, S1; primary motor cortex, M1; intraparietal cortex, Pcip; primary auditory cortex, A1; inferior temporal cortex, TCi; visual area 1, V1 and posterior cingulate cortex, PCC), we extracted the values from the whole brain matrices and applied a one-way analysis of variance (ANOVA). FC across the brain states were highlighted by displaying Z-score in inter-region matrices.

Event-related task fMRI analysis

We generated plots by obtaining the β-weight of SPM regressions of individual macaque data with the hemodynamic functions of the appropriate stimulus categories and then plotted the mean and SE of these β-weights. These values estimate, in percentages of the whole-brain fMRI signal, the size of the fMRI activation relative to the implicit rest baseline that divides trials.

The first level analyses consisted in the convolution of the stimulus categories with the MION canonical hemodynamic response function (HRF) and its time derivative. We also added motion regressors and heart rate as variables of non-interest to the event-related regressors. Activation time series of all the fMRI voxels were computed for each fMRI run and signal change expressed in T-score maps for the different stimulus categories relative to rest periods. Global standard trials that immediately followed a global deviant trial were excluded.

-Extended Results

Thalamic DBS effects on resting-state networks in anesthetized macaques

Static Functional Correlations (Figure 4, S6)

To test for statistical significance of connectivity between brain regions in different experimental

conditions, Student t-tests were performed with the null hypothesis of zero correlation. We calculated for each experimental condition *c* and sessions the average of positive and negative Z values of $Z_{c,s}$. The average positive Z-value was 0.43+/-2.9e-4 in the awake state, 0.22+/-2.6e-4 under anesthesia, 0.27+/-2.5e-4 during low CT-DBS, 0.39+/-2.8e-4 during high CT-DBS, 0.28+/-3.1e-4 during low VL-DBS and 0.19+/-2.4e-4 during high VL-DBS *(Figure 4A)*. Positive Z-values were significantly different under anesthesia and high CT-DBS (p < 0.001, FDR corrected *(Figure 4A)*. The average negative Z-value was 0.24+/-4.2e-4 in the awake state, 0.09+/-1.7e-4 under anesthesia, 0.12+/-2.7-4 during low CT-DBS, 0.11+/-3.2e-4 during high CT-DBS, 0.08+/-2.7e-4 during low VL-DBS and 0.08+/-2.2e-4 during high VL-DBS *(Figure 4A)*. Negative Z-values were significantly different under anesthesia and high CT-DBS (p < 0.001, FDR corrected) *(Figure 4A)*. In the awake state, the frontal cortex (areas 9/46, 8A, 6V and M1), parietal cortex (parietal area PFG and ventral intraparietal area), anterior and posterior cingulate cortices, temporal cortex (area A1) and occipital cortex (area V1) were strongly correlated to each other *(left column, Figure 4B)*.

Dynamic Functional Correlations (Figure 5, S7-S8, Table S4)

We applied k-means to the whole acquired dataset (including all experimental conditions) to cluster brain states *(Figure S7)*. We also applied k-means to two data subsets, subset CT and subset VL, to specifically characterize the effects of CT-DBS and VL-DBS respectively. Subset CT included data from awake, anesthesia and anesthesia + high CT-DBS conditions *(Figure 5A-C)*. Subset VL included data from awake, anesthesia and anesthesia + high VL-DBS conditions *(Figure 5D-E)*.

-Clustering subset CT (data from awake, anesthesia and anesthesia + high CT-DBS conditions) (Figure 5A-C)

In the awake state, all 7 brain states were represented with a similar probability of occurrence (β=0.45; R^2=0.22; p=0.28). During anesthesia, state 7 (with the highest function-structure similarity) was dominant and state 1 (with the lowest function-structure similarity) never occurred (β=1.91; R^2=0.67; p=0.02). Under high CT-DBS, consistent with partial recovery of consciousness, the probability of occurrence of state 7 decreased in favor of all the other brain states, especially state 2 and 3 (β=0.64; R^2=0.28; p=0.22). We computed the slope of the linear relation between structural and functional

correlations for each recording session and compared the slope distributions in the awake, anesthesia and DBS conditions. Awake and high CT-DBS slopes were significantly lower than the anesthesia slopes, indicating that a greater diversity of states were explored in the wake state (awake versus anesthesia: t-test, p=6e-5 and BF10=338, high CT-DBS versus anesthesia: t-test, p=0.001 and BF10=23). Importantly no differences were observed between awake and high CT-DBS slopes (t-test, p=0.42, BF01=3.28) *(Figure 5A-B, Table S6)*. In the awake state, the mean rank of brain states was 4 (4.38±1.28), for anesthesia, the mean rank was 6 (5.70±1.40) and during high CT-DBS, the mean rank was 5 (4.55±1.17). This brain state distribution was significantly different (ANOVA; F(2;120)=12.52; p=1.15e-7). Also, the frequency of brain state 7 was moderate in the awake experiments (probability=0.24), high during anesthesia (probability=0.58) and low again during high CT-DBS (probability=0.26; p<0.0001) *(Figure 5B)*. The probability of brain state 7 was higher in anesthesia compared to the awake state (t-test, p=1e-6, BF10=9063) and to high CT-DBS (t-test, p=1e-5, BF10=1017) which did not differ significantly (t-test, p=0.74, BF01=4.18). The mean similarity with the anatomical connectivity was also significantly different with 0.24 (±0.06) for the awake state, 0.31 (±0.07) for anesthesia and 0.25 (±0.06) for high CT-DBS (ANOVA; F(2,120)=14.75; p=1.87e-6). Anatomically, the functional brain states 1, 2 and 3, that were most characteristic of the awake, presented strong correlations within the "macaque GNW" prefrontal (dorsolateral prefrontal cortex, PFCdl; dorsolateral premotor cortex, PMCdl), parietal (intraparietal cortex, PCip) and cingulate nodes (anterior cingulate cortex, ACC; posterior cingulate cortex, PCCr), whereas state 7 displayed low or null Z-score values across the same entire cortical network *(Figure 5C)*. During high CT-DBS, the average duration of brain state 7 decreased compared to anesthesia (high CT-DBS versus anesthesia, p=2.48e-3; bootstrap analysis) and was similar to the awake state *(Figure S8)*.

- Clustering subset VL (data from awake, anesthesia and anesthesia + high VL-DBS conditions) (Figure 5D-E)

In the awake state, all seven brain states were present (β=0.16; R^2=0.02; p=0.74). Under anesthesia, brain state 7 was dominant (β=1.21; R^2=0.33; p=0.18), as under high VL-DBS (β=1.69; R^2=0.43; p=0.11) *(Figure 5F-G)*. We also computed the slope corresponding to each recording session and

compared the slope distributions in the awake, anesthesia and high VL-DBS conditions. Awake slopes were significantly lower compared to anesthesia and high VL-DBS slopes (awake versus anesthesia: t-test, p=0.0007 and BF10=39, awake versus high CT-DBS: t-test, p=1e-8 and BF10=636824). The slopes under anesthesia were smaller than the high VL-DBS slopes (t-test, p=0.01, BF10=3.81).

For the awake state, the mean rank of brain states was 4 (4.41±0.93), under anesthesia, the mean rank was 6 (5.81±1.39) and under high VL-DBS, the mean rank was 6 (6.41±0.38). The brain state distribution was significantly different (ANOVA; F(2;102)=32.03; p=1.61e-11). Brain state 7 was balanced in the awake state (awake brain state 7 probability=0.19), dominant under anesthesia (anesthesia brain state 7 probability=0.60) and high VL-DBS (high VL-DBS brain state 7 probability=0.72; p<0.0001) (*Figure 5F*). The probability of state 7 was smaller in the awake state compared to anesthesia (t-test, p=10e-9, BF10=2e7) and compared to high VL-DBS (t-test, p=10e-17, BF10=1e15). However, we found no evidence for a difference nor a similarity between anesthesia and high VL-DBS (t-test, p=0.15, BF10=0.64, BF01=1.54).

Brain state 1 highlighted strong correlations to all the tested cortical areas. Brain state 7 presented weak Z-score values with prefrontal (PFCdl; PMCdl), parietal (PCip) and cingulate cortex (ACC; PCC) *(Figure 5C)*.

With high VL-DBS, the average duration of brain state 7 increased compared to the awake state (high VL-DBS v/s awake, p<0.0001, bootstrap analysis) and was similar to the anesthesia state *(Figure S8)*.

-Clustering the whole dataset (data from awake, anesthesia, low CT-DBS, high CT-DBS, low VL-DBS and high VL-DBS) (Figure S7)

The occurrence of brain states in the awake condition was equiprobable (β=0.05; R^2=0.003; p=0.91). Under anesthesia, this probability was shaped by the brain state 7 (β=1.87; R^2=0.59; p=0.04). For the DBS sessions, brain state probability of occurrence was partly dominated by brain state 7 in the low CT-DBS condition (β=1.09; R^2=0.49; p=0.08), balanced under high CT-DBS (β=0.28; R^2=0.059; p=0.6308), partly dominated by the brain state 7 during the low VL-DBS experiments (β=1.52; R^2=0.59; p=0.04) and dominated by brain state 7 in the high VL-DBS condition (β=2.57; R^2=0.73; p=0.01) *(Figure S7)*.

The mean rank was 4 in the awake state (3.81±1.05), 5 under anesthesia (5.37±1.34), 5 in the low CT-DBS condition (4.81±0.93), 4 in the high CT-DBS condition (4.14±1.03), 5 in the low VL-DBS condition (5.35±0.86) and 6 in the high VL-DBS condition (6.01±0.53). The brain state distribution was significantly different between structural and functional correlations (ANOVA; $F(5;193)=19.65$; $p=8.31e-16$) *(Figure S7)*. The probability of occurrence of brain state 7 was low in the awake state (0.20), and high under anesthesia (0.54). Crucially, even though anesthesia continued, low CT-DBS and high CT-DBS reduced this probability down to an aware level (respectively 0.37 and 0.23). The probability of state 7 also decreased with low VL-DBS (0.38) but returned to high (0.63, $p<0.001$) under high VL-DBS. The mean similarity with the anatomical connectivity was also significantly different with 0.26 (±0.04) for the awake state, 0.32 (±0.06) for anesthesia, 0.29 (±0.04) for low CT-DBS, 0.27 (±0.04) for high CT-DBS, 0.31 (±0.0.04) for low VL-DBS and 0.35 (±0.03) and for high VL-DBS (ANOVA; $F(5,193)=17.72$; $p=1.94e-14$). The average duration of brain state 7 significantly decreased with high CT-DBS compared to the anesthesia state ($p=1.61e-9$, bootstrap analysis) and was similar to the awake state. Low CT-DBS and low VL-DBS decreased the duration the brain state 7 compared to anesthesia (low CT-DBS v/s anesthesia, $p=6.72e-8$; low VL-DBS v/s anesthesia, $p=1.88e-7$, bootstrap analysis). Under high VL-DBS, duration of the brain state 7 was similar to the anesthesia state (high VL-DBS v/s anesthesia, not significant) *(Figure S7)*.

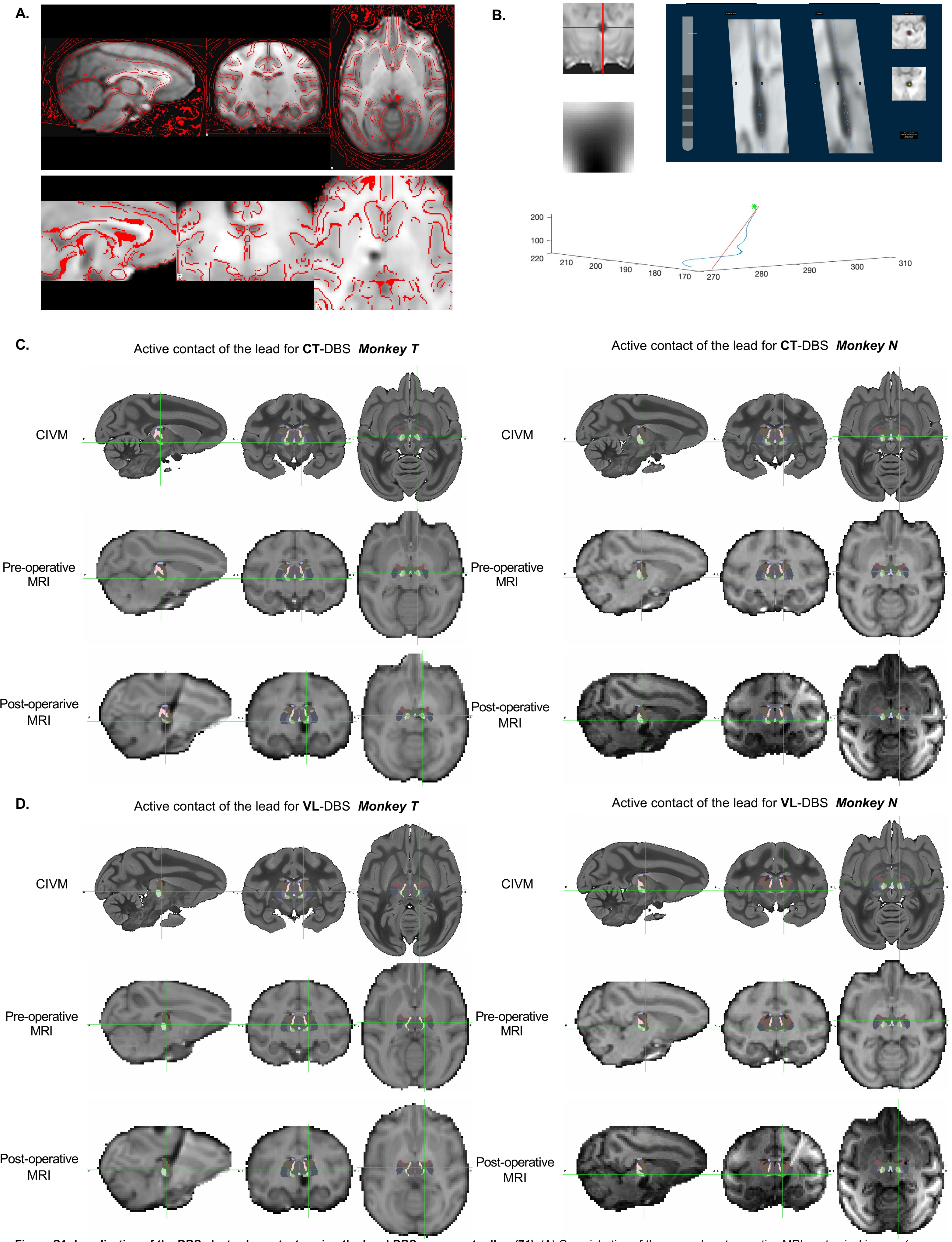

Figure S1: Localization of the DBS electrode contacts using the Lead-DBS macaque toolbox*(71)*. (A) Coregistration of the pre and post-operative MRI anatomical images (upper panel) and between MRI post-operative and MNI macaque brain atlas*(73)* (lower panel) for monkey T. (B) Pre-reconstruction of the electrode lead trajectory using the entry point on the anatomical MRI image and manual correction of electrode localization adjusting the most inferior (contact 0) and most superior (contact 3) DBS contacts according to the electrode artifact in two dimensional planes presented orthogonally. (C) Location of the centro-median (CM) DBS contact and (D) ventral-lateral thalamus (VL) DBS contact in monkey T (left column) and monkey N (right column) on the sagittal, coronal and axial plan. The target is displayed in the CIVM MRI atlas*(76)* (upper panel), pre-operative structural MRI (middle panel) and post-operative structural MRI (lower panel) warped in the MNI macaque space*(73)*.

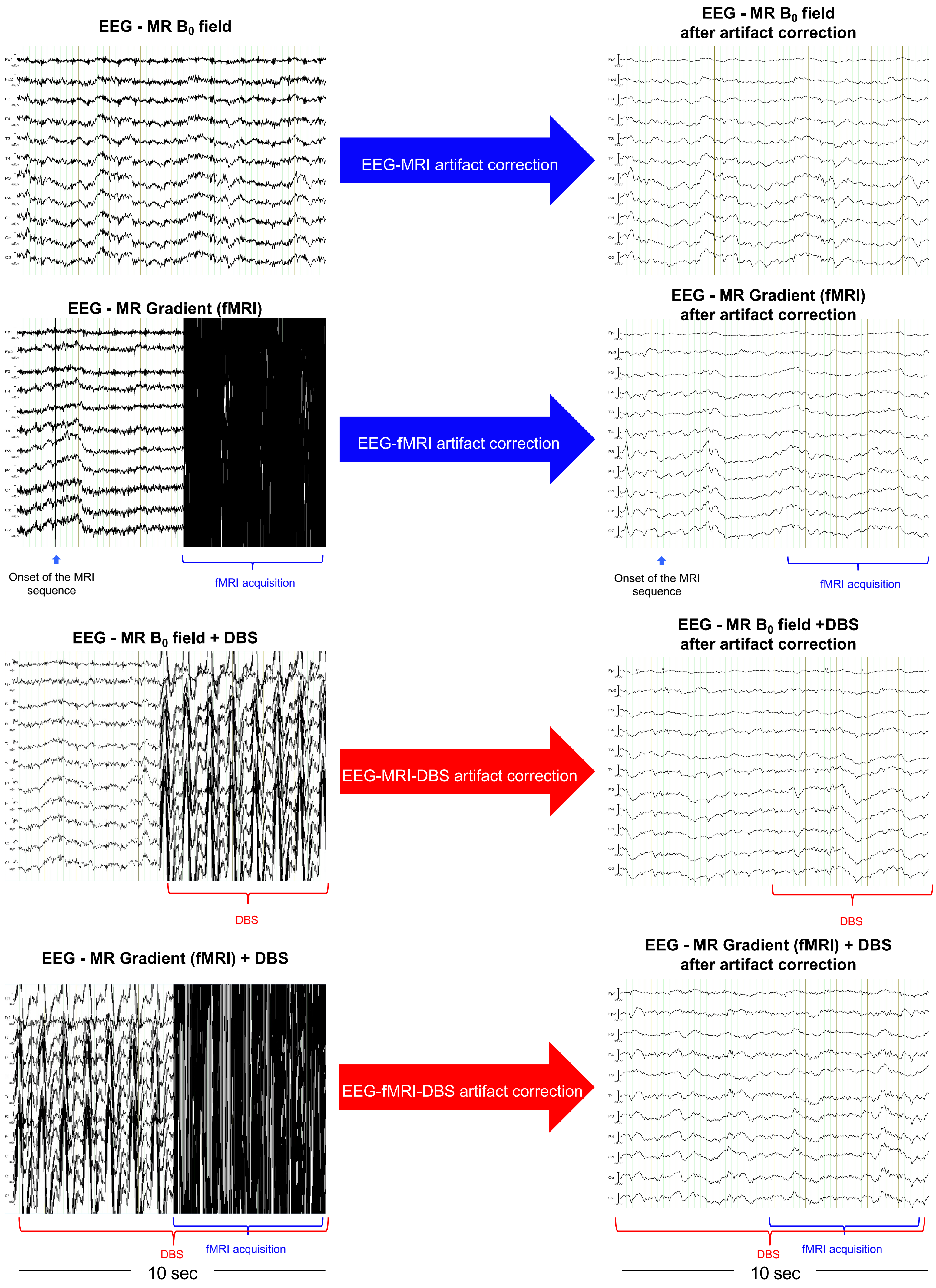

Figure S2: Suppression of EEG artifacts related to MR B_0 field, MR Gradients during fMRI acquisition and DBS. Examples of EEG recordings in anesthetized macaques inside a 3T MRI scanner without and with DBS of central thalamus (CT) thalamus at 3V.

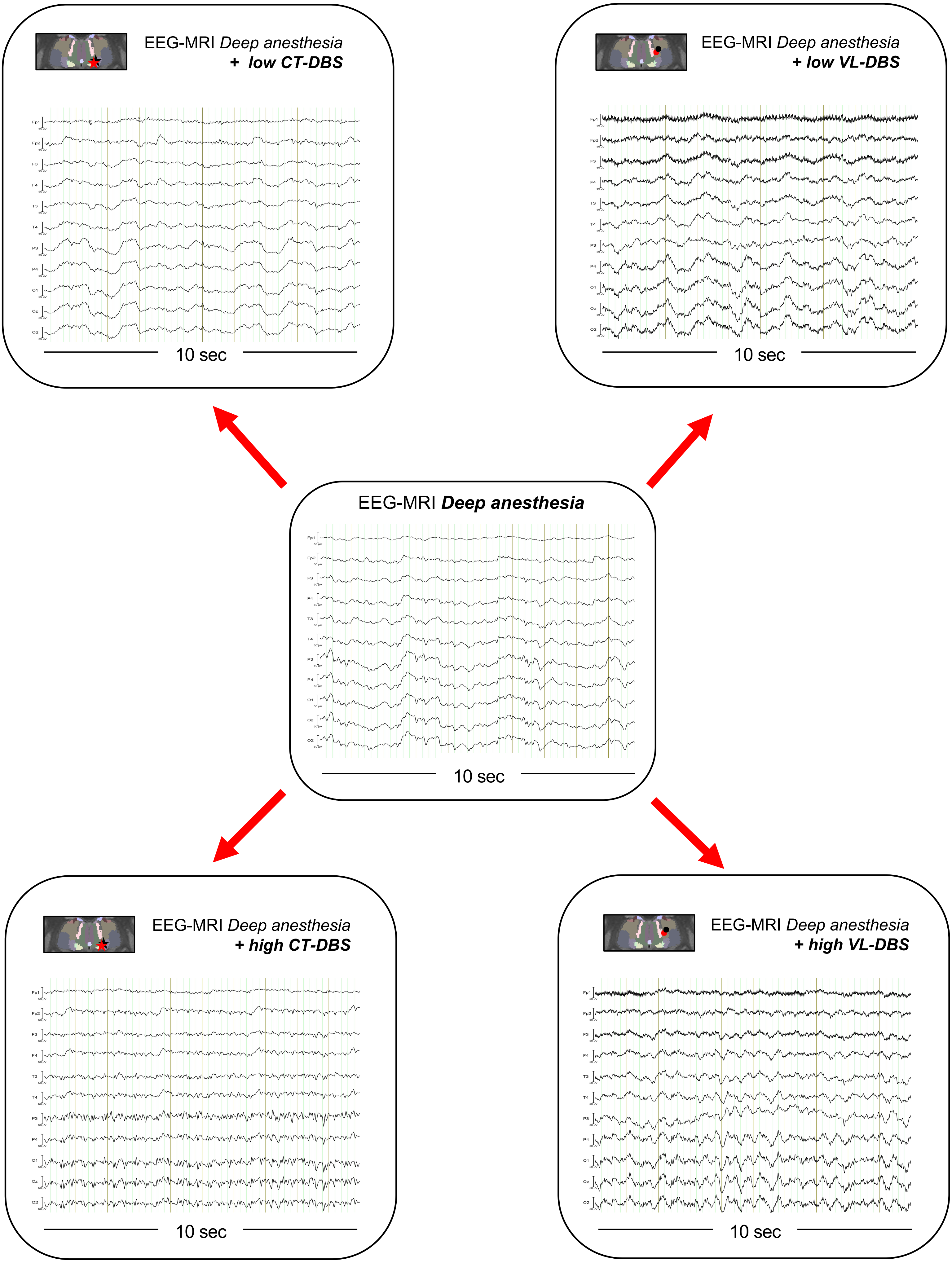

<u>Figure S3</u>: Effects of thalamic DBS on EEG. Examples of EEG recordings in anesthetized macaques inside a 3T MRI scanner with DBS of central thalamus (CT) or ventral-lateral thalamus (VL) at low or high voltages. The DBS-induced changes in cortical activity depends on the anatomical site of the active DBS lead contact and the intensity of the electrical stimulation.

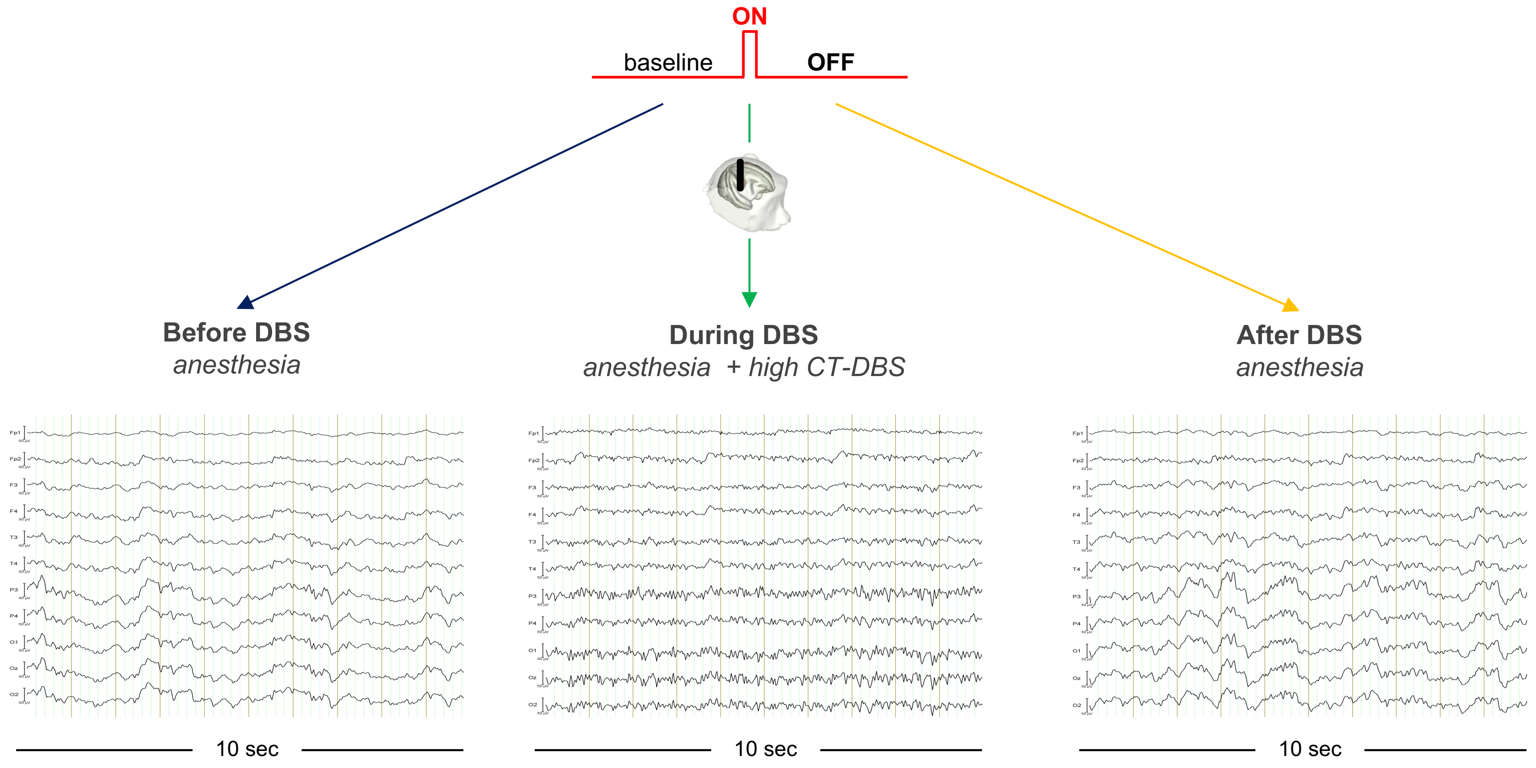

Figure S4: Cortical activity dynamic during and immediately after DBS

Examples of EEG recordings in anesthetized macaques inside a 3T MRI scanner before, during and after DBS of central thalamus (CT) at high voltage.

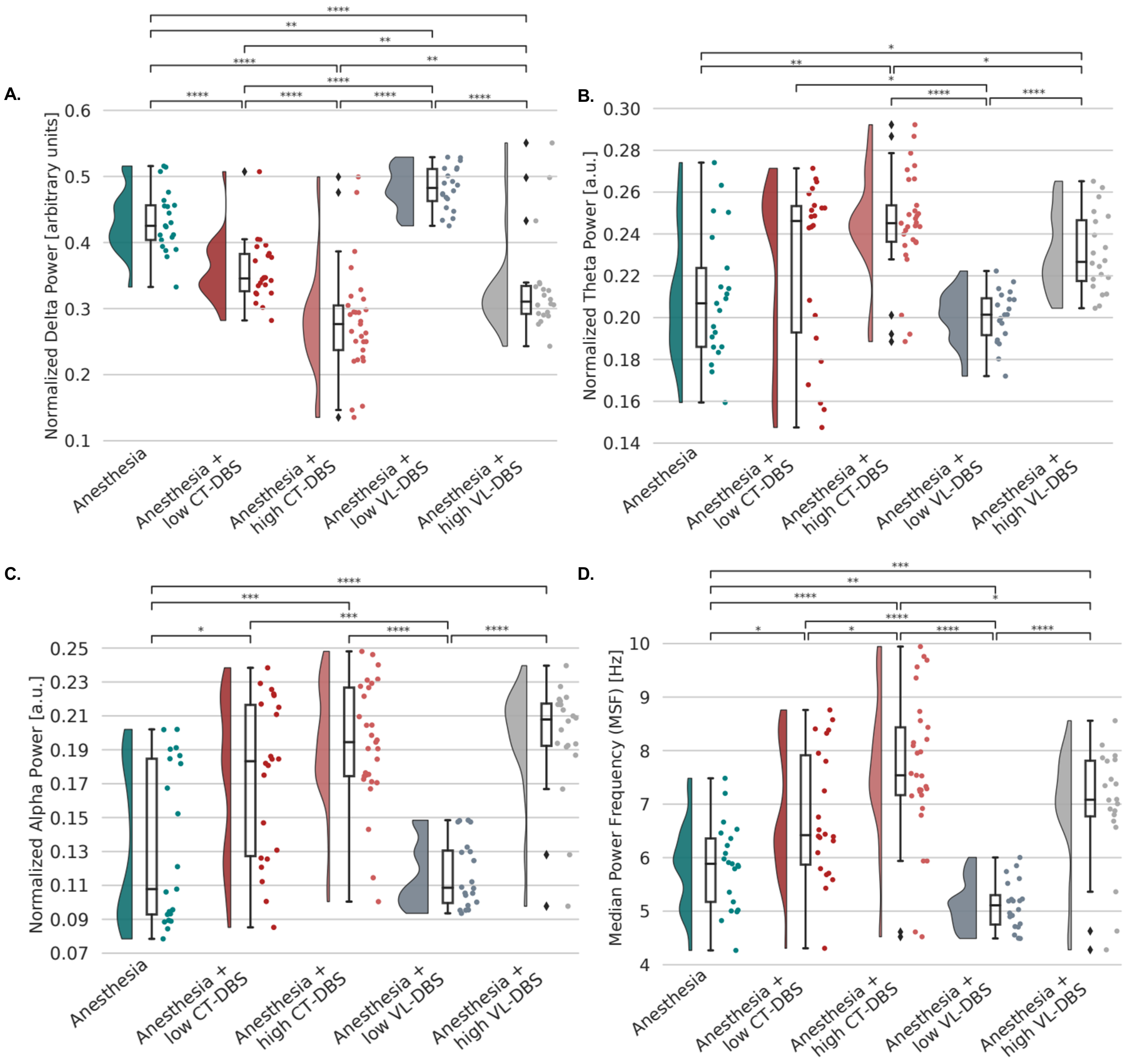

<u>Figure S5</u>: Modulation of normalized spectral power and median power frequency in the DBS conditions compared to anesthesia.

Distributions of the average values of normalized spectral power of (A) delta (1-4 Hz), (B) theta (4-8 Hz) and (C) alpha (8-13 Hz) oscillatory bands and (D) median power frequency (MSF) calculated on the epochs of the four stimulation conditions and under anesthesia. MSF is the frequency that divides the power spectrum in two equal areas. The figures consist of a distribution - smoothened version of a histogram, a box plot and a representation of the data points. Each dot in the figure represents the average value of a given marker across epochs during one recording session. a.u., arbitrary units. The significance lines represent FDR corrected Mann-Whitney U two-sided tests (see Methods). p-value annotation legend: ns: 5.00e-02 < p <= 1.00e+00, *: 1.00e-02 < p <= 5.00e-02, **: 1.00e-03 < p <= 1.00e-02, ***: 1.00e-04 < p <= 1.00e-03, ****: p <= 1.00e-04. P-values are FDR corrected.

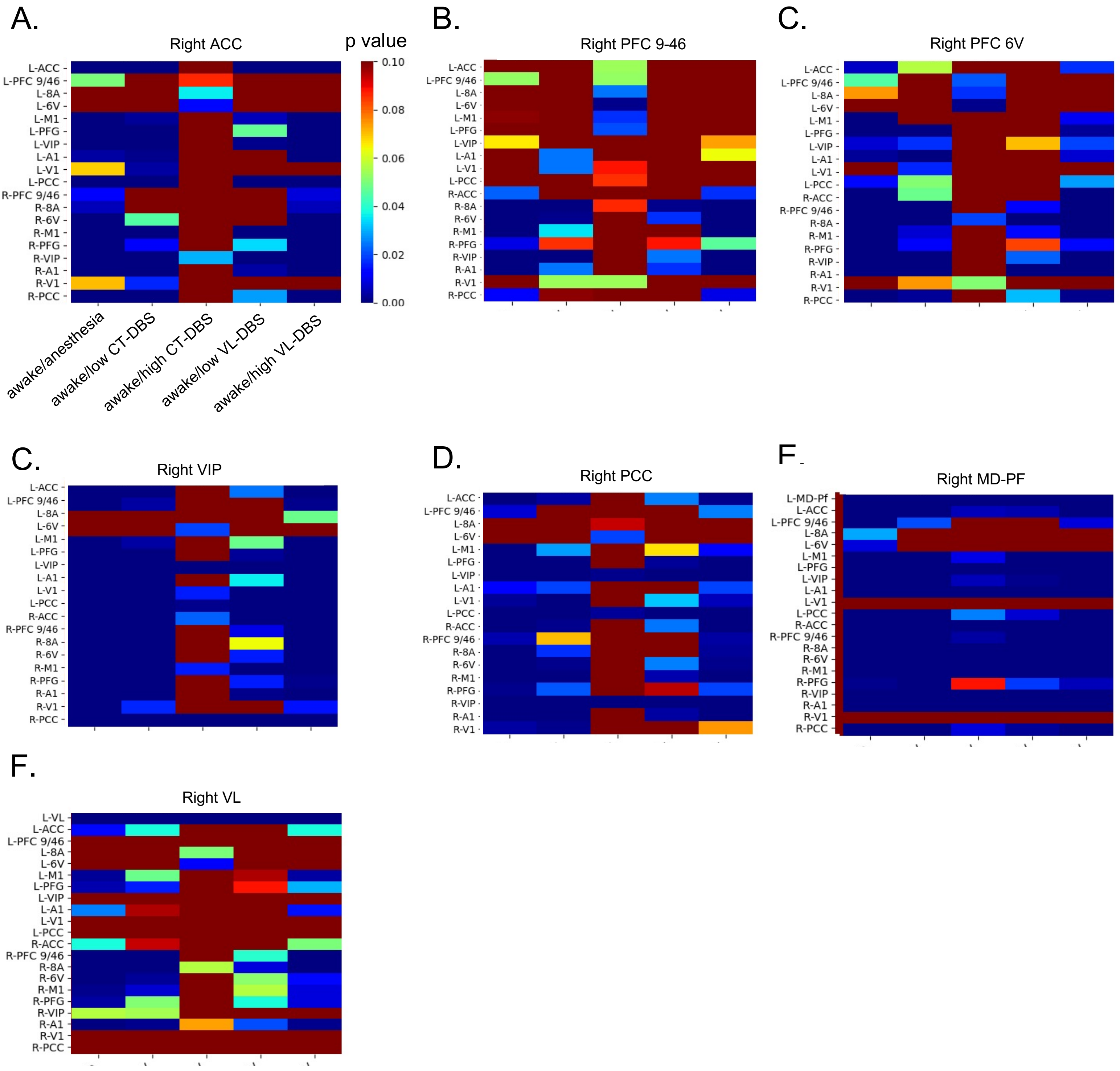

<u>Figure S6</u>: p values matrices for the ANOVA comparison of the awake state versus different experimental conditions of the static functional correlations within the macaque Global Neuronal Workspace (GNW) nodes and sensori-motor areas.

Statistical p values obtained by ANOVA to compare static functional correlations within the macaque GNW nodes and sensori-motor areas in the different experimental conditions. X-axis displays the experimental conditions (awake, anesthesia, low central thalamic (CT) DBS, high CT-DBS, low ventral-lateral thalamic (VL) DBS and high VL-DBS states), y-axis represents the macaque GNW areas correlated to the seed ($p < 0.001$, FDR corrected). For each region, the matrix stands for the p value for the comparison of the awake state versus anesthesia, low CT-DBS, high CT-DBS, low VL-DBS and high VL-DBS between the seed and the rest of the macaque GNW nodes and sensori-motor areas.

Anterior cingulate cortex (ACC) ; Prefrontal cortex (area 9/46, 8A, 6 Ventral) ; Primary motor cortex (M1) ; Parietal cortex (area PFG) ; Ventral Intraparietal sulcus (VIP) ; Primary auditory cortex (A1) ; Primary visual area (V1) ; Posterior cingulate cortex (PCC).

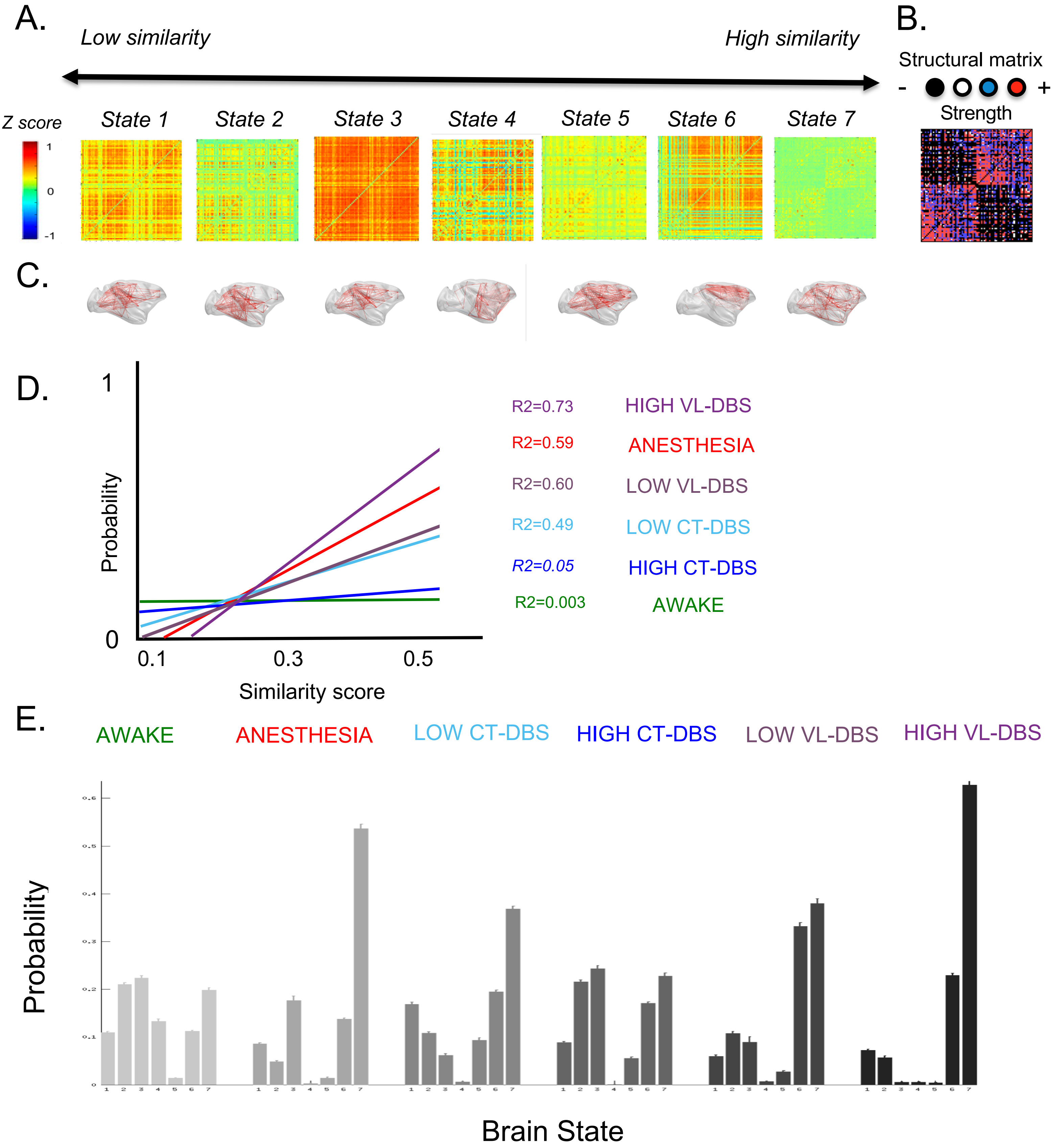

<u>Figure S7</u>: DBS effect on cortical dynamical correlations

(A) Seven functional brain states obtained by unsupervised clustering of the Z score matrix (all conditions pooled together, awake state, anesthesia, low central thalamic (CT) DBS, high CT-DBS, low ventral-lateral thalamic (VL) DBS and high VL-DBS). (B) Structural connectivity matrix derived from the CoCoMac atlas of anatomical macaque cortical connectivity. Colors represent the four grades of connection intensity (black=0; white=1; blue=2 and red=3). (C) Brain renders displaying the 400 strongest links for each functional brain state. Red line represent positive connections between regions of interests; blue represent negative connections. (D) Probability of occurrence of each functional brain state as a function of the similarity with the structural connectivity, for the awake state (green), anesthesia (red), low CT-DBS (light blue), high CT-DBS (dark blue), low VL-DBS (light purple) and high VL-DBS (dark purple). (E) Probability distributions of functional brain states for the for the awake state (green), anesthesia (red), low CT-DBS (light blue), high CT-DBS (dark blue), low VL-DBS (light purple) and high VL-DBS (dark purple). Error bars stand for 1 SEM.

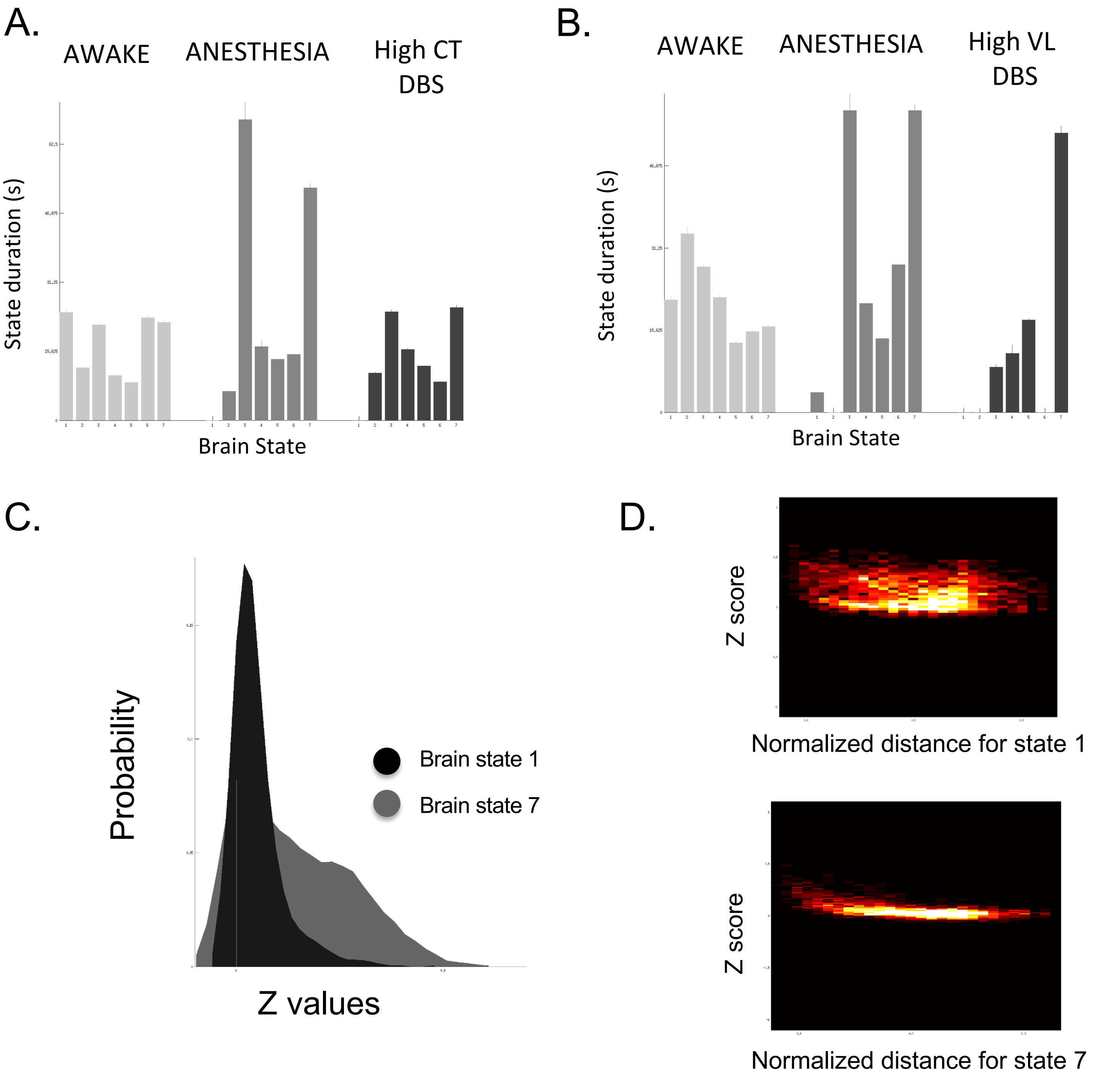

<u>Figure S8</u>: Average life time of brain states in the awake state, under anesthesia and during high cental thalamic (CT) DBS (left) or high ventral-lateral thalamic (VL) DBS (right); normalized probability distribution and two-dimensional normalized histograms

Average life time of brain states for the awake, anesthesia and high central thalamic (CT) DBS condition (A) or high ventral-lateral thalamic (VL) DBS (B) for 7 brain states obtained by k-means clustering. Error bars stand for 1 SEM. Normalized probability distribution of all Z values for the functional brain state 1 (the least similar to the structural brain connectivity) and functional brain state 7 (the most similar to the structural brain connectivity) for awake, anesthesia and high CT-DBS resting state pooled together. Similar results were obtained regardless the inputs conditions for the clustering (C). Two-dimensional normalized histograms for functional brain state 1 and functional brain state 7 for the clustering of awake, anesthesia and high CT-DBS condition. (D) Z values as a function of distance between pairs of regions of interest for brain state 1 (upper right) and brain state 7 (lower right) for the clustering of awake, anesthesia and high CT-DBS condition.

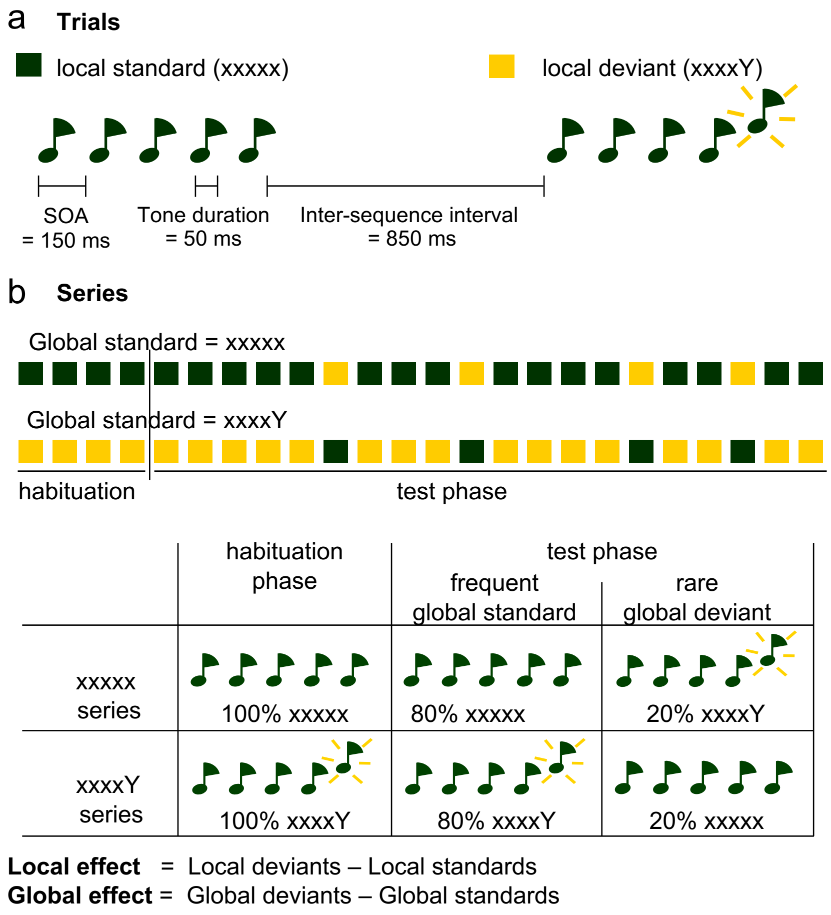

<u>Figure S9</u>: Local-global auditory paradigm

Description of the event-related auditory paradigm called local-global used in the auditory event-related fMRI experiments. Local deviants occur at the trial level (1st order) whereas Global deviants occur at the series level (2nd order).

fMRI activations during the auditory "Local-global" experiment

Local effect: High CT- DBS > Anesthesia

Individual results: Monkey N

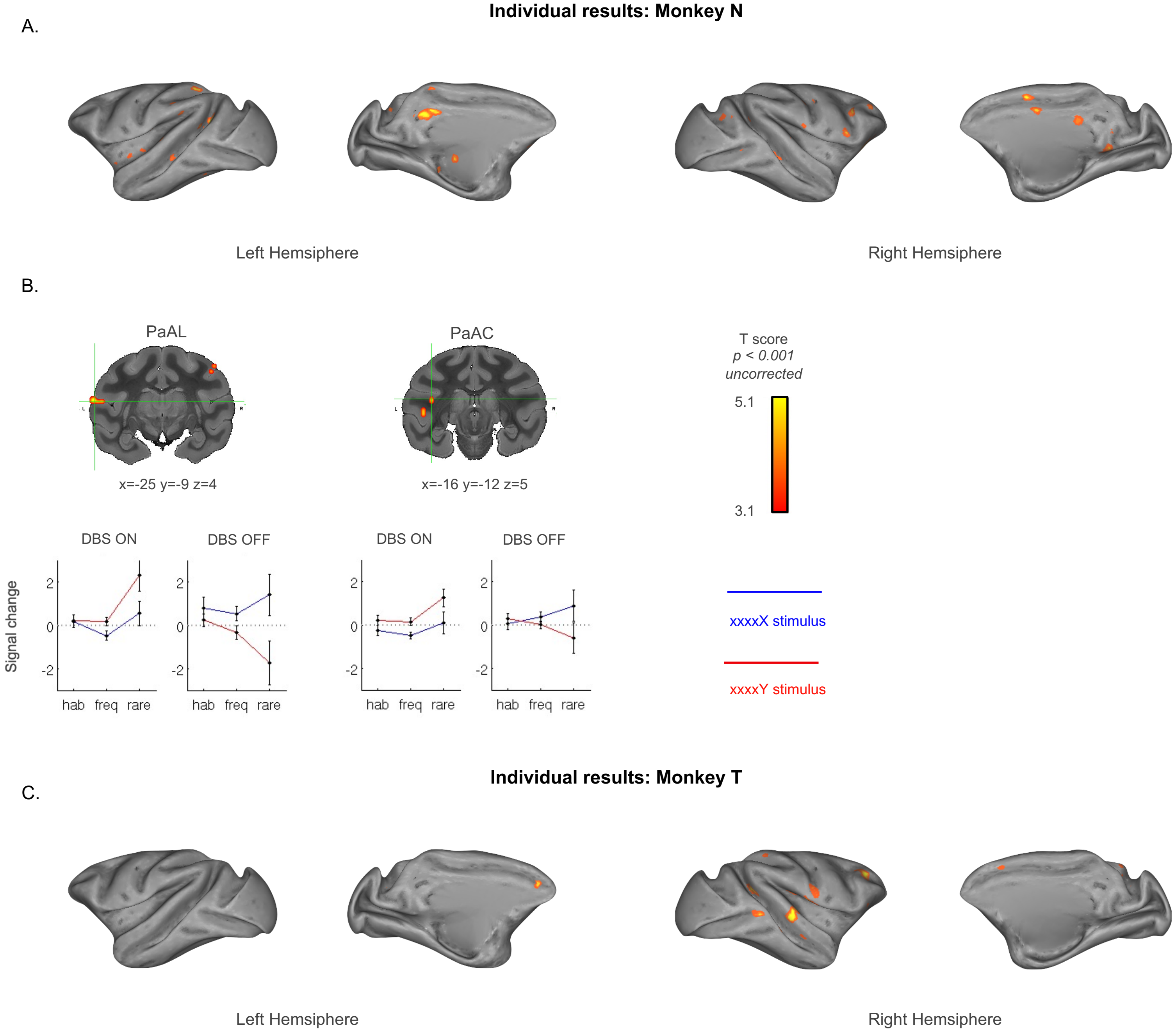

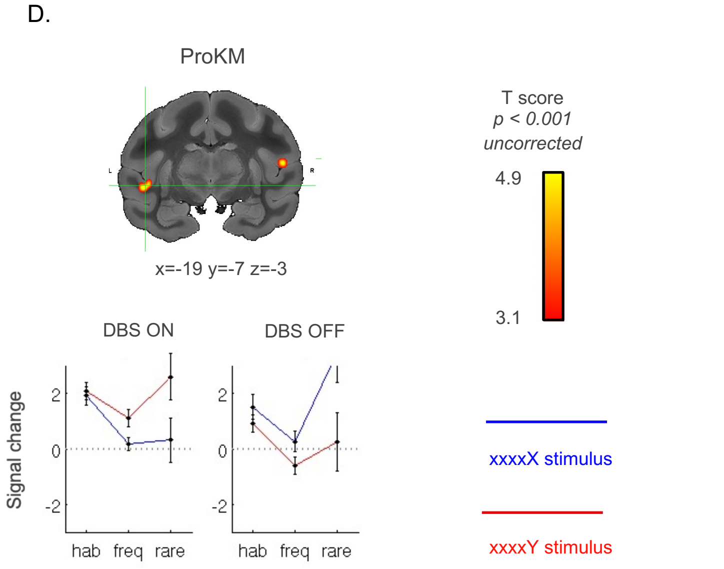

<u>Figure S10</u>: Activations maps for the local effect showing stronger activations under high central thalamic (CT) DBS compared to anesthesia state
Activation maps for the local effect showing stronger activations under high CT-DBS compared to anesthesia state (A, C). fMRI signal changes in areas responsive to the local effect (green cursor) (B,D). Individual results for monkey N (top panel - A,B) and monkey T (lower panel - C,D), p < 0.001, uncorrected. *PaAL, paraauditory cortex, lateral part; PaAc, paraauditroy cortex, caudal part; ProKM, auditory cortex, prokonio cortex , medial part.*

fMRI activations during the auditory “Local-global” experiment

Global effect: High CT-DBS > Anesthesia

Individual results: Monkey N

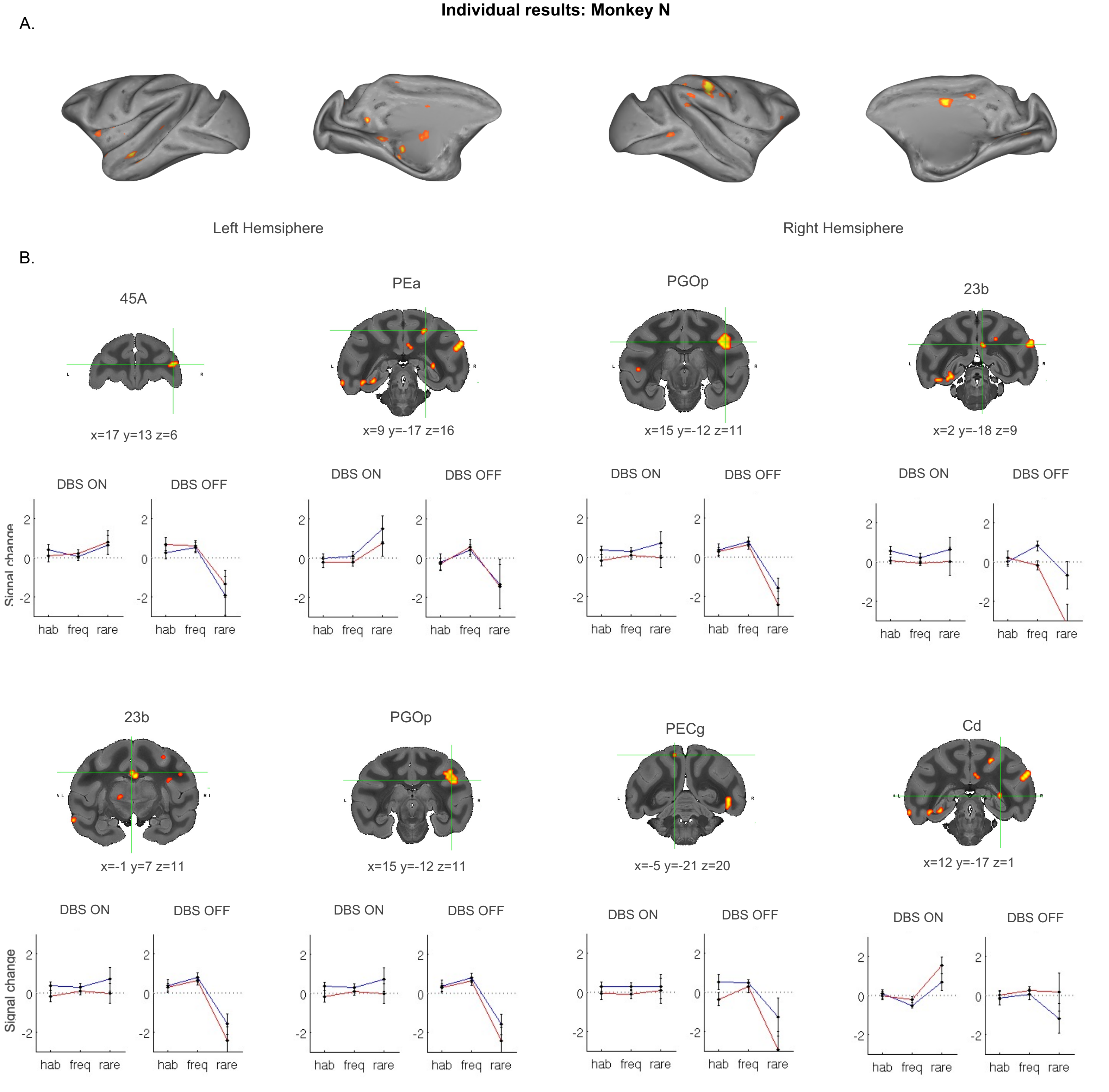

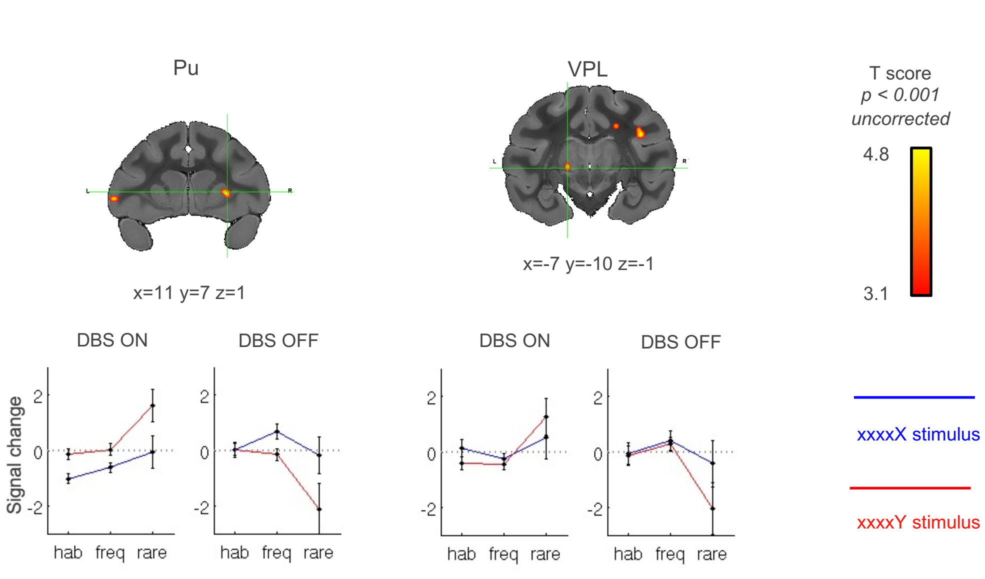

<u>Figure S11</u>: Activations maps for the global effect showing stronger activations under high central thalamic (CT) DBS compared to anesthesia state for monkey N. Activation maps for the local effect showing stronger activations under high CT-DBS compared to anesthesia state (A). fMRI signal changes in areas responsive to the local effect (green cursor) (B). Individual results for monkey N, p < 0.001, uncorrected.

Cd, Caudate nucleus; Pu, Putamen; VPL, ventro-postero-lateral thalamus.

fMRI activations during the auditory “Local-global” experiment

Global effect: High CT-DBS > Anesthesia

Individual results: Monkey T

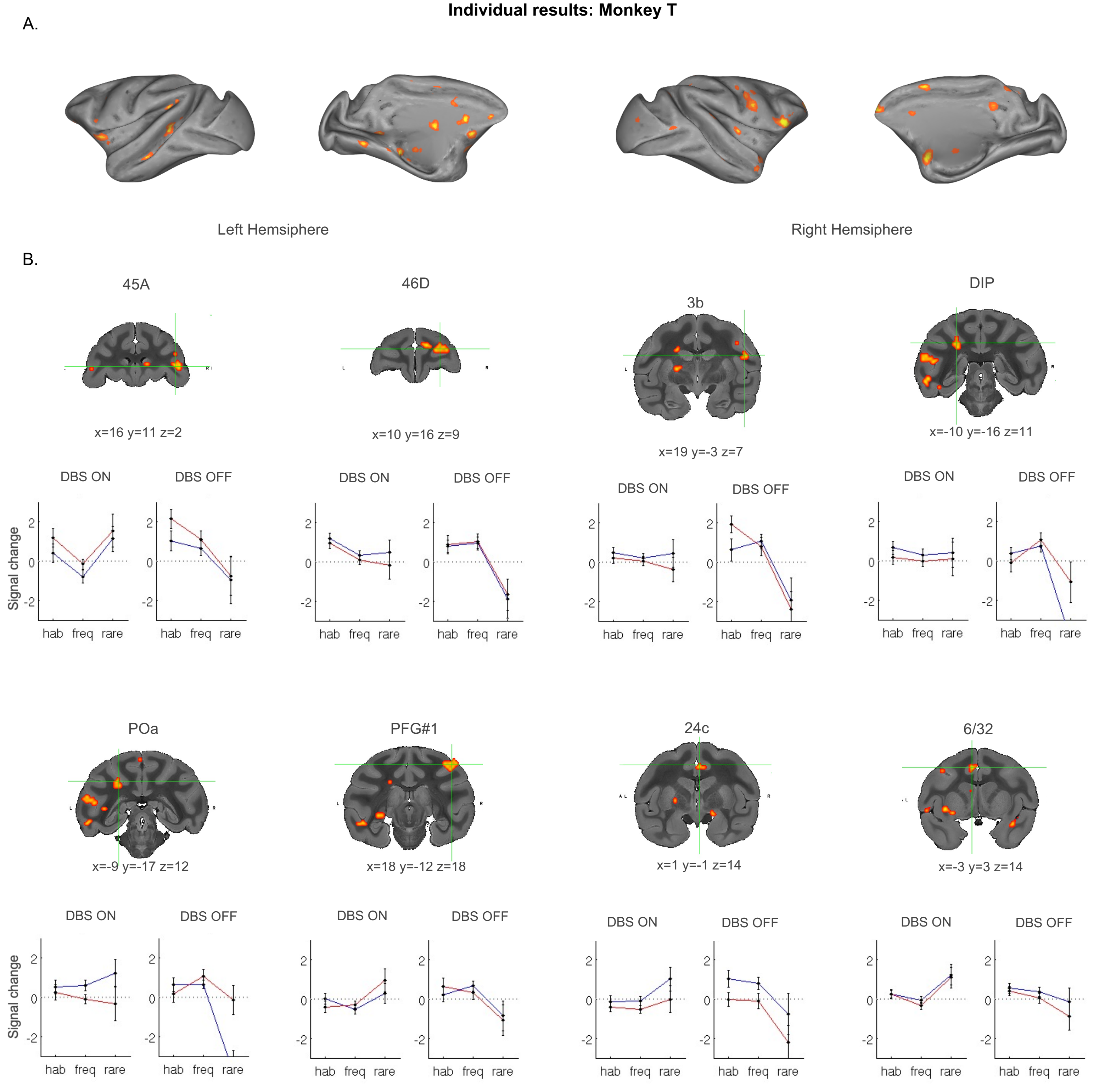

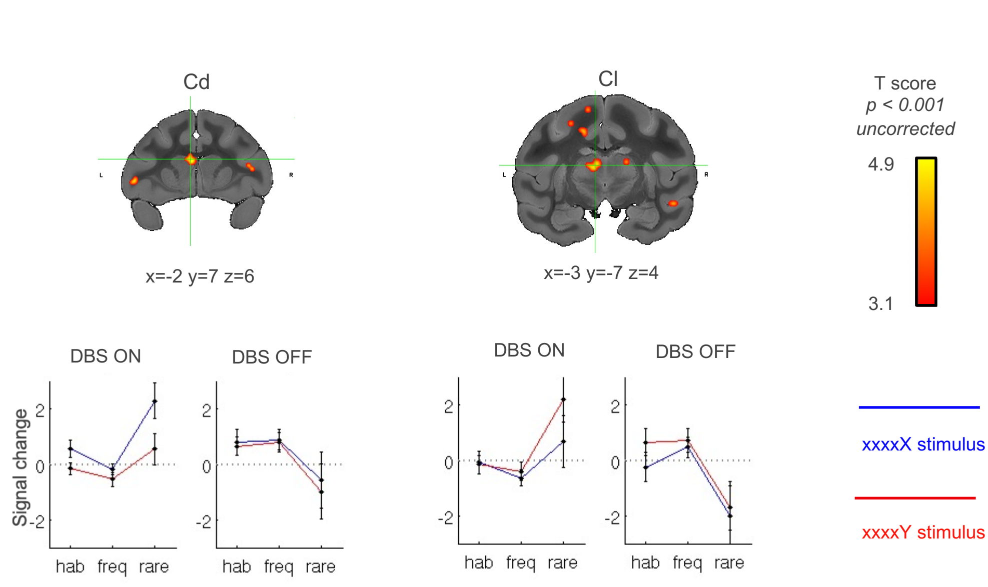

<u>Figure S12</u>: Activations maps for the global effect showing stronger activations under high central thalamic (CT) DBS compared to anesthesia state for monkey T. Activation maps for the local effect showing stronger activations under high CT-DBS compared to anesthesia state (A). fMRI signal changes in areas responsive to the local effect (green cursor) (B). Individual results for monkey N, $p < 0.001$, uncorrected.

Depth of Intraparietal sulcus (DIP), Caudate nucleus (Cd); centro-lateral thalamus (Cl).

Simulation of the thalamic nuclei activated during the DBS experiments

Thalamic nuclei	Abbreviation	CT-DBS				VL-DBS			
		low		high		low		high	
		monkey N	monkey T	monkey N	monkey T	monkey N	monkey T	monkey N	monkey T
Ventral-Postero-Medial	VPM	X	X	X	X		X	X	X
Centro-Median, Medial part	CMM	X	X	X	X				X
Centro-Median, Lateral part	CML	X	X	X	X				X
Medio-Dorsal, Central part	MDC	X	X	X	X		X	X	X
Ventral-Postero-Lateral	VPL			X	X	X	X	X	X
Medio-Dorsal, Lateral part	MDL			X	X	X	X	X	X
Ventral-Lateral, Medial part	VLM			X	X	X	X	X	X
Medio-Dorsal-Medial part	MDM			X	X				X
Inter-Medio-Dorsal	IMD			X	X				
Centro-Lateral	CL					X	X	X	X
Medio-Dorsal, Dorsal part	MDD					X		X	
Lateral-Dorsal, SuperFicial part	LDSF							X	
Ventral-Anterior, Lateral part	VAL							X	X
Ventral-Lateral, Lateral part	VLL							X	

<u>Table S1</u>: Simulation of the stimulated thalamic nuclei around the DBS lead using the LEAD DBS macaque toolbox

Estimation of the thalamic nuclei that were included in the volume of activated tissue around the DBS lead active contact*(71)* for monkey N and monkey T across the four experimental conditions (low central thalamic (CT) DBS, high CT-DBS, low ventral thalamic (VL) DBS and high VL-DBS).

Physiological data during the resting state fMRI experiments

Condition	Animal	Heart Rate (*HR in bpm*)	Oxygen Saturation (*SpO_2 in %*)	Blood pressure: Systolic (*SBP in mmHg*)	Blood pressure: Diastolic (*DBP in mmHg*)	Blood pressure: Mean (*MBP in mmHg*)	Respiration Rate (*RR in breath/min*)	End-tidal CO_2 (*$EtCO_2$ in mmHg*)	Temperature (*T in °C*)
Anesthesia	monkey N	111	98	116	59	87	20	41	37.0
		± 9	*± 2*	*± 10*	*± 7*	*± 9*	*± 1*	*± 2*	*± 0.7*
	monkey T	116	97	95	46	68	18	37	38.3
		± 17	*± 3*	*± 8*	*± 7*	*± 9*	*± 2*	*± 2*	*± 0.7*
Low CT-DBS	monkey N	134	99	128	70	101	21	43	37.1
		± 13	*± 1*	*± 13*	*± 12*	*± 13*	*± 2*	*± 2*	*± 0.3*
	monkey T	123	99	98	47	71	18	39	38.6
		± 18	*± 1*	*+/- 12*	*+/- 6*	*+/- 8*	*+/- 2*	*+/- 2*	*+/- 0.5*
High CT-DBS	monkey N	164	98	129	71	99	21	45	37.3
		± 16	*± 2*	*± 15*	*± 15*	*± 14*	*± 1*	*± 3*	*± 0.4*
	monkey T	181	98	133	74	104	19	43	39.6
		+/- 19	*+/- 2*	*+/- 12*	*+/- 12*	*+/- 13*	*+/- 2*	*+/- 2*	*+/- 0.4*
Low VL-DBS	monkey T	145	98	113	54	81	19	38	39.0
		± 3	*± 2*	*± 14*	*± 12*	*± 12*	*± 2*	*± 1*	*± 0.5*
High VL-DBS	monkey T	163	98	127	71	99	18	42	39.1
		± 14	*± 1*	*± 15*	*± 19*	*± 19*	*± 2*	*± 2*	*± 0.6*

<u>Table S2</u>: Physiological data during the fMRI resting-state experiments.

Oxygen saturation (SpO_2); systolic, diastolic and mean blood pressure (respectively SBP, DBP, MBP); respiration rate (RR); end-tidal CO_2 ($EtCO_2$) and temperature (T) for each animal (monkey N and T) under general anesthesia, general anesthesia plus low central thalamic (CT) DBS, general anesthesia plus high CT-DBS, general anesthesia plus low ventral-lateral thalamic (VL) DBS and general anesthesia plus high VL-DBS.

fMRI activations during the DBS block-design experiment

Area	Abbreviation	Hemisphere	CT-DBS low T score	CT-DBS low p value	CT-DBS high T score	CT-DBS high p value	VL-DBS low T score	VL-DBS low p value	VL-DBS high T score	VL-DBS high p value
Orbitofrontal cortex										
Area 13 of cortex	13	Right		*n.s*	19.15	p_{FWE} < *1x10-12*		*n.s*		*n.s*
Area 13a of cortex	13a	Left	5.11	p_{FW} = *7.4x10-3*	5.11	p_{FWE} = *7.4x10-3*		*n.s*		*n.s*
Area 14o	14o	Right		*n.s*	5.91	p_{FWE} = *1.21x10-4*		*n.s*		*n.s*
Area 25 of cortex	25	Left	5.48	p_{FWE} = *9.66x10-4*	5.48	p_{FWE} = *9.66x10-4*		*n.s*		*n.s*
Orbitofrontal cortex	OPro	Right		*n.s*	19.15	p_{FWE} < *1x10-12*		*n.s*		*n.s*
Frontal cortex										
Are 4 of cortex (primary motor)	4#1	Left		*n.s*	18.47	p_{FWE} < *1x10-12*		*n.s*		*n.s*
		Right		*n.s*	18.47	p_{FWE} < *1x10-12*		*n.s*	9.94	p_{FWE} < *1x10-12*
Area 6 of cortex, Medial part (supplementary motor)	6M	Left		*n.s*	19.15	p_{FWE} < *1x10-12*		*n.s*		*n.s*
		Right		*n.s*	19.15	p_{FWE} < *1x10-12*		*n.s*	5.77	p_{FWE} = *2.67x10-4*
Area 6 of cortex, DorsoCaudal part (Matellis F2)	6DC	Right		*n.s*	18.47	p_{FWE} < *1x10-12*		*n.s*	12.81	p_{FWE} < *1.0x10-12*
Area 6 of cortex, DorsoRostral part Matellis F7)	6DR	Right		*n.s*	19.15	p_{FWE} < *1x10-12*		*n.s*	12.81	p_{FWE} < *1.0x10-12*
Area 6 of cortex, Ventral part, Caudal subdivision (Matellis F4)	6VC	Left		*n.s*	6.54	p_{FWE} = *2.37x10-6*		n.s		*n.s*
		Right		*n.s*	18.47	p_{FWE} < *1x10-12*		*n.s*		*n.s*
Area 6 of cortex, Ventral part, Rostral subdivision (Matellis F5)	6VR	Left		*n.s*	6.16	p_{FWE} = *2.54x10-5*		*n.s*		*n.s*
		Right		*n.s*	19.15	p_{FWE} < *1x10-12*		*n.s*	5.61	p_{FWE} = *6.87x10-4*
Area 6/32 of cortex	6 32	Left		*n.s*	19.15	p_{FWE} < *1x10-12*		*n.s*		*n.s*
		Right	4.96	p_{FWE} = *1.56x10-2*	19.15	p_{FWE} < *1x10-12*		*n.s*	5.77	p_{FWE} = *2.67x10-4*
Area 8A of cortex	8A	Left		*n.s*	5.79	p_{FWE} = *2.41x10-4*		*n.s*		*n.s*
		Right		*n.s*	18.47	p_{FWE} < *1x10-12*		*n.s*	12.81	p_{FWE} < *1.0x10-12*
Area 8of cortex, AnteroDorsal part	8AD	Left		*n.s*	7.10	p_{FWE} = *5.04x10-8*		n.s		*n.s*
		Right	5.92	p_{FWE} = *7.47x10-5*	18.47	p_{FWE} < *1x10-12*		*n.s*	12.81	p_{FWE} < *1.0x10-12*
Area 8 of cortex, AnteroVentral part	8AV	Right		*n.s*	19.15	p_{FWE} < *1x10-12*		*n.s*	12.81	p_{FWE} < *1.0x10-12*
Area 8B of cortex	8B	Left		*n.s*	7.10	p_{FWE} = *5.04x10-8*		*n.s*		*n.s*
		Right		*n.s*	18.47	p_{FWE} < *1x10-12*		*n.s*		*n.s*
Area 8/32 of cortex	8 32	Right		*n.s*	19.15	p_{FWE} < *1x10-12*		*n.s*		*n.s*
Area 9 of cortex, Medial part	9M	Left		*n.s*	18.47	p_{FWE} < *1x10-12*		*n.s*		*n.s*
		Right		*n.s*	14.54	p_{FWE} < *1x10-12*		*n.s*		*n.s*
Area 9/32 of cortex	9 32	Right	7.72	p_{FDR} = *2.84x10-10*		p_{FDR} = *2.84x10-10*		*n.s*		*n.s*
Area 9/46 of cortex	9 46	Right	6.98	p_{FWE} = *7.08x10-8*	19.15	p_{FWE} < *1x10-12*		*n.s*	9.94	p_{FWE} < *1x10-12*
Area 44 of cortex	44	Left		*n.s*	6.66	p_{FWE} = *1.03x10-6*		*n.s*		*n.s*
		Right		*n.s*	19.15	p_{FWE} < *1x10-12*		*n.s*	9.94	p_{FWE} < *1x10-12*
Area 45A of crotex	45A	Right	7.30	p_{FWE} = *7.23x10-9*	19.15	p_{FWE} < *1x10-12*		*n.s*	9.94	p_{FWE} < *1.0x10-12*
Area 45B of cortex	45B	Left		*n.s*	6.66	p_{FWE} = *1.03x10-6*		*n.s*		*n.s*
		Right	7.30	p_{FWE} = *7.23x10-9*	19.15	p_{FWE} < *1x10-12*		*n.s*	9.94	p_{FWE} < *1.0x10-12*
Area 46D of cortex	46D	Right	6.98	p_{FWE} = *7.08x10-8*		p_{FWE} = *7.08x10-8*		*n.s*		*n.s*
Area 46V of cortex	46V	Left		*n.s*	5.27	p_{FWE} = *4.68x10-3*		*n.s*		*n.s*
		Right	6.98	p_{FWE} = *7.08x10-8*		p_{FWE} = *7.08x10-8*		*n.s*		*n.s*
Area 47 (old 12) of cortex, Lateral part	47L	Left	6.27	p_{FWE} = *8.28x10-6*		p_{FWE} = *8.28x10-6*		*n.s*		*n.s*
Area 47 (old 12) of cortex, Orbital part	47O	Left		*n.s*	6.66	p_{FWE} = *1.03x10-6*		*n.s*		*n.s*
Area ProM (promotor)	ProM#1	Left		*n.s*	11.36	p_{FWE} < *1x10-12*		*n.s*		*n.s*

fMRI activations during the DBS block-design experiment

Area	Abbreviation	Hemisphere	CT-DBS low T score	CT-DBS low p value	CT-DBS high T score	CT-DBS high p value	VL-DBS low T score	VL-DBS low p value	VL-DBS high T score	VL-DBS high p value
Parietal cortex										
Area 1 of cortex (somatosensory)	1	Left		*n.s*	18.47	*p_{FWE} < 1x10-12*		*n.s*		*n.s*
		Right		*n.s*	18.47	*p_{FWE} < 1x10-12*		*n.s*	9.94	*p_{FWE} < 1x10-12*
Area 2 of cortex (somatosensory)	2#1	Left		*n.s*	18.47	*p_{FWE} < 1x10-12*		*n.s*		*n.s*
		Right		*n.s*	18.47	*p_{FWE} < 1x10-12*		*n.s*	12.81	*p_{FWE} < 1x10-12*
Area 2 of cortex, Vesticular part	2Ve			*n.s*	18.47	*p_{FWE} < 1x10-12*		*n.s*		*n.s*
Area 3a of cortex (somatosensory)	3a	Left		*n.s*	18.47	*p_{FWE} < 1x10-12*		*n.s*		*n.s*
		Right		*n.s*	18.47	*p_{FWE} < 1x10-12*		*n.s*	9.94	*p_{FWE} < 1x10-12*
Area 3b of cortex (somatosensory)	3b	Right		*n.s*	18.47	*p_{FWE} < 1x10-12*		*n.s*	16.05	*p_{FWE} < 1.0x10-12*
Depth IntraParietal area	DIP	Right		*n.s*	18.47	*p_{FWE} < 1x10-12*		*n.s*		*n.s*
Dorsal parietal area	Dpt	Left		*n.s*	7.27	*p_{FWE} = 1.53x10-8*		*n.s*	7.23	*p_{FWE} = 2.09x10-8*
		Right	5.73	*p_{FWE} = 2.32x10-4*	18.47	*p_{FWE} < 1x10-12*		*n.s*		*n.s*
OccipitoParietal area	OPt	Left		*n.s*	12.59	*p_{FWE} < 1x10-12*		*n.s*		*n.s*
		Right		*n.s*	18.47	*p_{FWE} < 1x10-12*		*n.s*		*n.s*
Parietal area PE	PE	Right	6.51	*p_{FWE} = 1.72x10-6*	18.47	*p_{FWE} < 1x10-12*		*n.s*	7.26	*p_{FWE} = 1.72x10-8*
Parieral area PE, Caudal part	PEC	Right		*n.s*	5.39	*p_{FWE} = 2.3x10-3*		*n.s*	7.85	*p_{FWE} = 2.08x10-10*
Parietal area PEa	PEa	Right	6.30	*p_{FWE} = 7.07x10-6*	18.47	*p_{FWE} < 1x10-12*		*n.s*	7.85	*p_{FWE} = 2.08x10-10*
Parietal area PF (cortex)	PFCx	Right		*n.s*		*n.s*		*n.s*	9.94	*p_{FWE} < 1x10-12*
Parieal area PF, Opercular part	PFOp	Left		*n.s*	7.27	*p_{FWE} = 1.53x10-8*		*n.s*		*n.s*
		Right		*n.s*	18.47	*p_{FWE} < 1x10-12*		*n.s*		*n.s*
Parietal area PG	PG	Right		*n.s*	12.62	*p_{FWE} < 1x10-12*		*n.s*		*n.s*
Parieal area PG, Opercular part	PGOp	Left		*n.s*	7.27	*p_{FWE} = 1.53x10-8*		*n.s*		*n.s*
		Right	7.38	*p_{FWE} = 3.90x10-9*	18.47	*p_{FWE} < 1x10-12*		*n.s*	5.95	*p_{FWE} = 9.51x10-5*
Parietal area POa, External part	POaE	Right		*n.s*	18.47	*p_{FWE} < 1x10-12*		*n.s*		*n.s*
Parietal area POa, Internal part	POaI	Right		*n.s*	18.47	*p_{FWE} < 1x10-12*		*n.s*		*n.s*
ParietoOccipital associated area in the intraparietal sulcus	POa	Left		*n.s*	7.27	*p_{FWE} = 1.53x10-8*		*n.s*		*n.s*
		Right		*n.s*	18.47	*p_{FWE} < 1x10-12*		*n.s*	9.94	*p_{FWE} < 1x10-12*
Posterior Parietal area	PPt	Right		*n.s*	18.47	*p_{FWE} < 1x10-12*		*n.s*		*n.s*
RetroInsular area, Parietal part	ReIP	Left		*n.s*	7.27	*p_{FWE} = 1.53x10-8*		*n.s*		*n.s*
		Right		*n.s*	12.62	*p_{FWE} < 1x10-12*		*n.s*		*n.s*
Secondary somatosensory cortex	S2	Left		*n.s*	7.27	*p_{FWE} = 1.53x10-8*		*n.s*		*n.s*
		Right		*n.s*	18.47	*p_{FWE} < 1x10-12*		*n.s*	5.66	*p_{FWE} = 5.12x10-4*
Secondary somatosensory cortex, External part	S2E	Right		*n.s*		*n.s*		*n.s*	12.81	*p_{FWE} < 1x10-12*
Secondary somatosensory cortex, Internal part	S2I	Right		*n.s*	18.47	*p_{FWE} < 1x10-12*		*n.s*		*n.s*
Visual area 4, Dorsal part	V4D	Right		*n.s*	18.47	*p_{FWE} < 1x10-12*		*n.s*		*n.s*
Visual area 4, Transitional part	V4T	Right		*n.s*	18.47	*p_{FWE} < 1x10-12*		*n.s*		*n.s*
Visual area 4A	V4A	Left		*n.s*	7.27	*p_{FWE} = 1.53x10-8*		*n.s*	7.23	*p_{FWE} = 2.09x10-8*
		Right		*n.s*	18.47	*p_{FWE} < 1x10-12*		n.s		*n.s*
Cingulate cortex										
Area 23 of cortex	23	midline	6.84	*p_{FWE} = 1.82x10-7*		*n.s*		*n.s*		*n.s*
Area 23a of cortex	23a	midline		*n.s*	5.60	*p_{FWE} = 7.36x-4*		*n.s*		*n.s*
Area 23b of cortex	23b			*n.s*	18.47	*p_{FWE} < 1x10-12*		*n.s*		*n.s*
Area 23c of cortex	23c	Left		*n.s*	18.47	*p_{FWE} < 1x10-12*		*n.s*		*n.s*
		Right		*n.s*	18.47	*p_{FWE} < 1x10-12*		*n.s*		*n.s*
Area 24/23b of cortex	24 23b	Right		*n.s*	18.47	*p_{FWE} < 1x10-12*		*n.s*		*n.s*
Area 24/23c of cortex	24 23c	Left		*n.s*	18.47	*p_{FWE} < 1x10-12*		*n.s*		*n.s*
		Right		*n.s*		*n.s*		*n.s*	5.77	*p_{FWE} = 2.67x10-4*
Area 24b of cortex	24b	Left		*n.s*	18.47	*p_{FWE} < 1x10-12*		*n.s*		*n.s*
		Right		*n.s*	18.47	*p_{FWE} < 1x10-12*		*n.s*		*n.s*
Area 24c of cortex	24c	Left		*n.s*	18.47	*p_{FWE} < 1x10-12*		*n.s*		*n.s*
		Right	6.98	*p_{FWE} = 7.08x10-8*	18.47	*p_{FWE} < 1x10-12*		*n.s*		*n.s*
Area 24d of cortex	24d	Left		*n.s*	18.47	*p_{FWE} < 1x10-12*		*n.s*		*n.s*
Area 31 of cortex	31	Left		*n.s*	18.47	*p_{FWE} < 1x10-12*		*n.s*		*n.s*
		Right		*n.s*	18.47	*p_{FWE} < 1x10-12*		*n.s*		*n.s*
Area PGM/31 of cortex	PGM 31	midline	5.41	*p_{FWE} = 1.4x10-3*		*n.s*		*n.s*		*n.s*
Parietal area PE, Cingulate part	PECg	Right		*n.s*	18.47	*p_{FWE} < 1x10-12*		*n.s*		*n.s*

fMRI activations during the DBS block-design experiment

Area	Abbreviation	Hemisphere	CT-DBS low T score	CT-DBS low p value	CT-DBS high T score	CT-DBS high p value	VL-DBS low T score	VL-DBS low p value	VL-DBS high T score	VL-DBS high p value
Temporal cortex										
Area PG associated, region of superior temporal sulcus	PGa	Right		*n.s*	18.47	p_{FWE} < 1x10-12		*n.s*		*n.s*
Auditory Koniocortex, Lateral part	AKL	Left		*n.s*	7.27	p_{FWE} = 1.53x10-8		*n.s*		*n.s*
		Right		*n.s*	18.47	p_{FWE} < 1x10-12		*n.s*		*n.s*
Auditory Koniocortex, Medial part	AKM	Left		*n.s*	7.27	p_{FWE} = 1.53x10-8		*n.s*		*n.s*
		Right		*n.s*	18.47	p_{FWE} < 1x10-12		*n.s*		*n.s*
Fundus of Superior Temporal sulcus	FST	Left		*n.s*	6.48	p_{FWE} = 3.37x10-6		*n.s*		*n.s*
		Right		*n.s*	18.47	p_{FWE} < 1x10-12		*n.s*		*n.s*
Medial Superior Temporal area	MST	Left		*n.s*	7.27	p_{FWE} = 1.53x10-8		*n.s*		*n.s*
		Right		*n.s*	18.47	p_{FWE} < 1x10-12		*n.s*		*n.s*
Middle Temporal area (visual area 5)	MT	Left		*n.s*	7.27	p_{FWE} = 1.53x10-8		*n.s*		*n.s*
		Right		*n.s*	18.47	p_{FWE} < 1x10-12		*n.s*	6.08	p_{FWE} = 4.26x10-5
ParaAuditory area, Caudal part	PaAC	Left		*n.s*	7.27	p_{FWE} = 1.53x10-8		*n.s*		*n.s*
		Right		*n.s*	20.85	p_{FWE} < 1x10-12		*n.s*		*n.s*
ParaAuditory area, lateral part	PaAL	Right		*n.s*	18.47	p_{FWE} < 1x10-12		*n.s*		*n.s*
ParaAuditory area, Rostral part	PaAR	Right		*n.s*	12.62	p_{FWE} < 1x10-12		*n.s*		*n.s*
ProKoniocortex	ProK	Right		*n.s*	18.47	p_{FWE} < 1x10-12		*n.s*		*n.s*
RetroInsular area	ReI	Right		*n.s*	18.47	p_{FWE} < 1x10-12		*n.s*		*n.s*
RetroInsular area, Temporal part	ReIT	Left		*n.s*	7.27	p_{FWE} = 1.53x10-8		*n.s*		*n.s*
		Right		*n.s*	18.47	p_{FWE} < 1x10-12		*n.s*		*n.s*
Superior Temporal sulcus area 1	ST1	Right		*n.s*	8.83	p_{FWE} < 1x10-12		*n.s*		*n.s*
Superior Temporal area, gyral part	ST2g	Left		*n.s*	11.36	p_{FWE} < 1x10-12		*n.s*		*n.s*
Superior Temporal area, sulcal part	ST2s	Left		*n.s*	11.36	p_{FWE} < 1x10-12		*n.s*		*n.s*
Temporal area TAa	TAa	Right		*n.s*	18.47	p_{FWE} < 1x10-12		*n.s*		*n.s*
Temporal area TE, Medial part	TEM	Right		*n.s*	18.47	p_{FWE} < 1x10-12		*n.s*		*n.s*
Temporal area TE, OccipitoMedial part	TEOM	Right		*n.s*	18.47	p_{FWE} < 1x10-12		*n.s*		*n.s*
Temporal area TEa	TEa	Right		*n.s*	18.47	p_{FWE} < 1x10-12		*n.s*		*n.s*
Temporal ParietoOccipital associated area in sts	TPO	Right	7.38	p_{FWE} = 3.90x10-9	18.47	p_{FWE} < 1x10-12		*n.s*		*n.s*
Temporal ParietoOccipital associated area in sts, Caudal part	TPOC	Left		*n.s*	7.27	p_{FWE} = 1.53x10-8		*n.s*		*n.s*
		Right		*n.s*	18.47	p_{FWE} < 1x10-12		*n.s*		*n.s*
Temporoparietal cortex	Tpt	Left	5.02	p_{FWE} = 1.15x10-2		*n.s*		*n.s*		*n.s*
		Right	7.38	p_{FWE} = 3.90x10-9	18.47	p_{FWE} < 1x10-12		*n.s*		*n.s*
Occipital cortex										
Visual area 1 (primary visual cortex	V1	Left		*n.s*		*n.s*		*n.s*	9.22	p_{FWE} < 1x10-12
		Right	7.22	p_{FWE} = 1.26x10-8	18.47	p_{FWE} < 1x10-12		*n.s*	7.44	p_{FWE} = 4.52x10-9
Visual area 2	V2	Left		*n.s*	5.70	p_{FWE} = 4.08x10-4		*n.s*		*n.s*
		Right	8.65	p_{FWE} < 1x10-12	18.47	p_{FWE} < 1x10-12	7.97	p_{FWE} = 3.94x10-11	9.27	p_{FWE} < 1x10-12
Visual area 3, Dorsal part	V3D	Right		*n.s*	18.47	p_{FWE} < 1x10-12		*n.s*		*n.s*
Visual area 3A	V3A	Left		*n.s*	7.27	p_{FWE} = 1.53x10-8		*n.s*		*n.s*
		Right		*n.s*		*n.s*		*n.s*	5.63	p_{FWE} = 6.10x10-4
Insular cortex										
Dysgranular Insular cortex	DI	Left		*n.s*	6.18	p_{FWE} = 2.29x10-5		*n.s*		*n.s*
		Right		*n.s*	12.62	p_{FWE} < 1x10-12		*n.s*		*n.s*
Granular Insular cortex	GI	Left		*n.s*	7.52	p_{FWE} = 2.54x10-9		*n.s*		*n.s*
		Right		*n.s*	18.47	p_{FWE} < 1x10-12		*n.s*	5.32	p_{FWE} = 1.54x10-2
Insular Proisocortex	IPro	Right		*n.s*	12.32	p_{FWE} < 1x10-12		*n.s*		*n.s*
Striatum										
Caudate nucleus	Cd	Left		*n.s*	18.47	p_{FWE} < 1x10-12		*n.s*		*n.s*
		Right	6.34	p_{FWE} = 5.50x10-6	18.47	p_{FWE} < 1x10-12		*n.s*		*n.s*
Putamen	Pu	Left	5.30	p_{FWE} = 2.6x10-3	18.47	p_{FWE} < 1x10-12		*n.s*		*n.s*
		Right	13.68	p_{FWE} < 1x10-12	18.47	p_{FWE} < 1x10-12		*n.s*	5.30	p_{FWE} = 3.7x10-3

continue

fMRI activations during the DBS block-design experiment

Area	Abbreviation	Hemisphere	CT-DBS low T score	CT-DBS low p value	CT-DBS high T score	CT-DBS high p value	VL-DBS low T score	VL-DBS low p value	VL-DBS high T score	VL-DBS high p value
Thalamus										
Lateral Geniculate Nucleus	LGN	Right		*n.s*	18.47	p_{FWE} < 1x10-12		*n.s*		*n.s*
Lateral pulvinar	Lpul	Left		*n.s*	5.20	p_{FWE} = 1.44x10-2		*n.s*		*n.s*
Medial Geniculate nucleus, Ventral part	MGV	Right		*n.s*	5.87	p_{FWE} = 1.55x10-4		*n.s*		*n.s*
Medial pulvinar	Mpul	Right	5.59	p_{FWE} = 5.17x10-4	11.07	p_{FWE} < 1x10-12		*n.s*		*n.s*
MedioDorsal thalamic nucleus, Central part	MDC	Left	5.65	p_{FWE} = 3.56x10-4	11.07	p_{FWE} < 1x10-12		*n.s*		*n.s*
MedioDorsal thalamic nucleus, Dorsal part	MDD	Left		*n.s*	11.07	p_{FWE} < 1x10-12		*n.s*		*n.s*
MediDorsal thalamic nucleus, Medial part	MDM	Left		*n.s*	11.07	p_{FWE} < 1x10-12		*n.s*		*n.s*
Paraventricular thalamic nucleus	PV	Left		*n.s*	11.07	p_{FWE} < 1x10-12		*n.s*		*n.s*
		Right		*n.s*	11.07	p_{FWE} < 1x10-12		*n.s*		*n.s*
Paraventricular Thalamus	PVT	Left		*n.s*	11.07	p_{FWE} < 1x10-12		*n.s*		*n.s*
		Right		*n.s*	11.07	p_{FWE} < 1x10-12		*n.s*		*n.s*
Reticular thalamic nucleus	R#4	Right		*n.s*	5.91	p_{FWE} = 1.22x10-4		*n.s*		*n.s*
Hypothalamus										
Hypothalamus	Hy	Left		*n.s*	14.54	p_{FWE} < 1x10-12		*n.s*		*n.s*
Pallidum										
External Globus Pallidus	EGP	Left		*n.s*	5.25	p_{FWE} = 4.9x10-3		*n.s*		*n.s*
Paraseptal subpallium										
Accumbens nucleus, Core	AcbC	Left		*n.s*	18.47	p_{FWE} < 1x10-12		*n.s*		*n.s*
		Right		*n.s*	18.47	p_{FWE} < 1x10-12		*n.s*		*n.s*
Accumbens nucleus, Shell	AcbSh	Left		*n.s*	18.47	p_{FWE} < 1x10-12		*n.s*		*n.s*
		Right		*n.s*	18.47	p_{FWE} < 1x10-12		*n.s*		*n.s*
Basal nucleus, Meynert	BM	Left		*n.s*	14.54	p_{FWE} < 1x10-12		*n.s*		*n.s*
Substancia Inniominata	SI	Left		*n.s*	15.09	pWE < 1x10-12		*n.s*		*n.s*
Subpallial amygdala										
Anterior Amygdaloid area	AA	Left		*n.s*	14.54	p_{FWE} < 1x10-12		*n.s*		*n.s*
Bed nucleus of the Stria Terminallis IntraAmygdaloid	BSTIA	Left		*n.s*	15.09	p_{FWE} < 1x10-12		*n.s*		*n.s*
Central amygdaloid nucleus, Lateral division	CeL	Left		*n.s*	14.54	p_{FWE} < 1x10-12		*n.s*		*n.s*
Central amygdaloid nucleus, Medial division	CeM	Left		*n.s*	14.54	p_{FWE} < 1x10-12		*n.s*		*n.s*
Ventral pallium										
BasoMedial amygdaloid nucleus	BM	Left		*n.s*	14.54	p_{FWE} < 1x10-12		*n.s*		*n.s*
Medial amygdaloid nucleus	Me	Left		*n.s*	14.54	p_{FWE} < 1x10-12		*n.s*		*n.s*
Midbrain										
Midbrain	MD	Left	5.83	p_{FWE} = 1.25x10-4	8.32	p_{FWE} < 1x10-12		*n.s*		*n.s*
		Right		*n.s*	11.07	p_{FWE} < 1x10-12		*n.s*		*n.s*
Cerebellum										
Cerebellum	Cb	Left	8.38	p_{FWE} < 1x10-12	13.74	p_{FWE} < 1x10-12		*n.s*	7.53	p_{FWE} = 2.29x10-9
		Right	8.41	p_{FWE} < 1x10-12	14.03	p_{FWE} < 1x10-12		*n.s*	5.31	p_{FWE} = 1.8x10-3

<u>Table S3</u>: fMRI activations during low central thalamic (CT), high CT-DBS, low ventral-lateral thalamic (VL) and high VL-DBS

Thalamic DBS-induced fMRI activity during the electrical stimulation block-design experiment, $p < 0.05$, FWE corrected, ns: non significant.

Interpretation of the Bayes Factors

Value	BF10	BF01
>100	Obvious evidence for H1	Obvious evidence for H0
30 to 100	Very strong evidence for H1	Very strong evidence for H0
10 to 30	Strong evidence for H1	Strong evidence for H0
3 to 10	Substantial evidence for H1	Substantial evidence for H0
1 to 3	Anecdotal evidence for H1	Anecdotal evidence for H0
1	No evidence for H1 or H0	
1 to 0.33	Anecdotal evidence for H0	Anecdotal evidence for H1
0.33 to 0.10	Substantial evidence for H0	Substantial evidence for H1
0.10 to 0.03	Strong evidence for H0	Strong evidence for H1
0.03 to 0.01	Very strong evidence for H0	Very strong evidence for H1
<0.01	Obvious evidence for H0	Obvious evidence for H1

Table S4: Interpretation of the Bayes Factors.

Value of the Bayes Factor BF10 and BF01 to interpret statistical evidence in favor of the H1 or H0 hypothesis. A BF greater than 3 significatively support the evidence of the tested hypothesis.

fMRI activations during the auditory “Local-Global” experiment

Local effect

Group

Area	Abbreviation	Hemisphere	Anesthesia		High CT-DBS		High CT-BS > Anesthesia	
			T score	p value	T score	p value	T score	p value
Orbitofrontal cortex								
Orbital Proisocortex	OPro	Left	4.44	*$p_{FDR} = 0.047$*		*n.s*		*n.s*
Parietal cortex								
Area 3b of cortex (somatosensory)	3b	Left	4.59	*$p_{FDR} = 0.039$*		*n.s*		*n.s*
Parietal area PG#1	PG#1	Right		*n.s*		*n.s*	4.24	*$p_{FDR} = 0.044$*
Visual area 4, Ventral part	V4V	Left		*n.s*	4.80	*$p_{FDR} = 0.049$*		*n.s*
Cingulate cortex								
Parietal area PE, Cingulate part	PECg	Left	4.80	$p_{FDR} = 0.037$		*n.s*		*n.s*
Temporal cortex								
Fundus of Superior Temporal sulcus	FST	Left	4.27	$p_{FDR} = 0.048$		*n.s*		*n.s*
ProKoniocortex, Medial part	ProKM	Left		*n.s*		*n.s*	4.59	*$p_{FDR} = 0.040$*
Temporal ParietoOccipital associated area in STS	TPO	Right		*n.s*		*n.s*	4.42	*$p_{FDR} = 0.040$*
Temporoparietal cortex	Tpt	Right		*n.s*		*n.s*	4.38	*$p_{FDR} = 0.040$*
Occipital cortex								
Visual area 1 (primary visual cortex)	V1	Left	4.41	*$p_{FDR} = 0.047$*		*n.s*		*n.s*
Striatum								
Caudate nucleus	Cd	Right	4.28	$p_{FDR} = 0.048$		*n.s*		*n.s*
Midbrain								
Midbrain	MB	Right		*n.s*		*n.s*	4.13	*$p_{FDR} = 0.044$*

<u>Table S5</u>: Cerebral activations for the local effect

fMRI activations for the local effect under anesthesia, high central thalamic (CT) DBS and comparison between high CT-DBS > anesthesia. Group results, $p < 0.05$, FDR corrected, ns: non significant.

fMRI activations during the auditory “Local-Global” experiment

Global effect

Group

Area	Abbreviation	Hemisphere	Awake T score	Awake p value	Anesthesia T score	Anesthesia p value	High CT-DBS T score	High CT-DBS p value	High CT-DBS > Anesthesia T score	High CT-DBS > Anesthesia p value
Frontal cortex										
Area 6 of the cortex, DorsoRostral part (Matellis F7)	6DR	Right		n.s		n.s	3.66	p_{FDR} =0.023		n.s
Area 6 of the cortex, Ventral part, Rostral subdivision (Matellis F5)	6VR	Left	3.88	p_{FDR} =0.024		n.s		n.s		n.s
Area 8B of cortex	8B	Right	4.61	p_{FDR} =0.014		n.s		n.s		n.s
Area 8B of cortex	9/46V	Right	3.82	p_{FDR} =0.026		n.s		n.s		n.s
Area 44 ofcortex	44	Right	3.58	p_{FDR} =0.033		n.s	4.75	p_{FDR} =0.002	4.57	p_{FDR} =0.018
				n.s		n.s	3.48	p_{FDR} =0.031		n.s
Area 45B of cortex	45B	Left	3.92	p_{FDR} =0.024		n.s	3.94	p_{FDR} =0.014		n.s
		Right		n.s		n.s		n.s	4.57	p_{FDR} =0.018
Area 47 (old 12) of cortex, Orbital part	47O	Left	3.91	p_{FDR} =0.024		n.s	3.83	p_{FDR} =0.017		n.s
		Right	4.09	p_{FDR} =0.018		n.s		n.s		n.s
Area ProM (promotor)	ProM#1	Left		n.s		n.s	3.80	p_{FDR} =0.018		n.s
Parietal cortex										
Area 3a of cortex (somatosensory)	3a	Right		n.s		n.s	5.06	p_{FDR} =0.002		n.s
				n.s		n.s	3.50	p_{FDR} =0.030		n.s
				n.s		n.s	3.47	p_{FDR} =0.031		n.s
Area 3b of cortex (somatosensory)	3b	Left			4.73	p_{FDR} =0.042		n.s		n.s
Parietal area PFG	PFG#1	Right	4.20	p_{FDR} =0.017		n.s		n.s	4.29	p_{FDR} =0.018
Depth IntraParietal area	DIP	Left		n.s		n.s		n.s	4.06	p_{FDR} =0.028
		Right		n.s		n.s	4.11	p_{FDR} =0.010		n.s
Parietal area PG	PG#1	Right	3.76	p_{FDR} =0.028		n.s	3.98	p_{FDR} =0.013		n.s
Dorsal parietal area	Dpt	Right		n.s		n.s	4.86	p_{FDR} =0.002		n.s
Parietal area Poa, external part	POaE	Left	3.39	p_{FDR} =0.042		n.s	4.51	p_{FDR} =0.005		n.s
Parietal area Poa, internal part	POaI	Left	3.56	p_{FDR} =0.034		n.s		n.s		n.s
Secondary somatosensory cortex	S2	Right		n.s		n.s	3.25	p_{FDR} =0.043		n.s
Secondary soamatosensory cortex, External part	S2E	Right		n.s		n.s	3.70	p_{FDR} =0.021		n.s
Visual area 4, Ventral part	V4V	Left		n.s		n.s	3.39	p_{FDR} =0.035	4.32	p_{FDR} =0.018
Visual area 4, Trnasitional part	V4T	left	4.37	p_{FDR} =0.017		n.s				n.s
Visual area 4, Dorsal part	V4D	Left		n.s		n.s	3.58	p_{FDR} =0.026		n.s
		Right		n.s		n.s	3.76	p_{FDR} =0.019		n.s
Cingulate cortex										
Area 23c of cortex	23c	Left		n.s		n.s	3.60	p_{FDR} =0.024		n.s
Area 24b of cortex	23b	Left	3.45	p_{FDR} =0.039		n.s		n.s		n.s
Temporal cortex										
Medial Superior Temporal area	MST	Left		n.s		n.s	3.24	p_{FDR} =0.044		n.s
		Right	4.05	p_{FDR} =0.019	4.46	p_{FDR} =0.042		n.s		n.s
Fundus of Superior Temporal sulcus	FST	Left		n.s		n.s	3.33	p_{FDR} =0.039		n.s
Superior Temporal sulcus area, gyral part	ST2g	Left		n.s		n.s	3.76	p_{FDR} =0.019		n.s
retroinsular area, Temporal part	ReIT	Right	3.76	p_{FDR} =0.028		n.s		n.s		n.s
Temporal area TEa	TEa#1	Left	4.12	p_{FDR} =0.018		n.s	4.87	p_{FDR} =0.002	4.78	p_{FDR} =0.018
Temporal area TE, Occipital part	TEO	Left		n.s		n.s	3.27	p_{FDR} =0.042	4.32	p_{FDR} =0.018
Temporal ParietoOccipital associated area in STS	TPO	Left		n.s		n.s	5.37	p_{FDR} =0.002	4.62	p_{FDR} =0.018
				n.s		n.s		n.s	4.27	p_{FDR} =0.018
Occipital cortex										
Visual area 1 (primary visual cortex)	V1	Left			4.19	p_{FDR} =0.042		n.s		n.s
		Right			4.33	p_{FDR} =0.042	4.39	p_{FDR} =0.006		n.s
					4.30	p_{FDR} =0.042	3.90	p_{FDR} =0.015		n.s
Visual area 2	V2	Left				n.s	3.57	p_{FDR} =0.026		n.s
		Right	3.91	p_{FDR} =0.024	4.53	p_{FDR} =0.042	4.62	p_{FDR} =0.004		n.s
			3.58	p_{FDR} =0.033	4.46	p_{FDR} =0.042		n.s		n.s
Visual area 3, Ventral part	V3V	Right		n.s		n.s	4.27	p_{FDR} =0.007		n.s
				n.s		n.s	3.76	p_{FDR} =0.019		n.s
Visual area 3, Dorsal part	V3D	Right		n.s		n.s	3.88	p_{FDR} =0.015		n.s
Visual area 3A	V3A	Right		n.s		n.s	3.70	p_{FDR} =0.021		n.s

fMRI activations during the auditory “Local-Global” experiment

Global effect

Group

Area	Abbreviation	Hemisphere	Awake T score	Awake p value	Anesthesia T score	Anesthesia p value	High CT-DBS T score	High CT-DBS p value	High CT-DBS > Anesthesia T score	High CT-DBS > Anesthesia p value
Striatum										
Caudate nucleus	Cd	Right	4.61	$p_{FDR} = 0.014$		*n.s*	4.83	$p_{FDR} = 0.002$		*n.s*
Putamen	Pu	Right	4.66	$p_{FDR} = 0.007$		*n.s*	4.27	$p_{FDR} = 0.007$		*n.s*
				n.s		*n.s*	3.77	$p_{FDR} = 0.019$		*n.s*
Thalamus										
Ventral PosteroLateral thalamic nucleus	VPL#1	Right			4.72	$p_{FDR} = 0.042$		*n.s*		*n.s*
Ventral Anterior thalamic nucleus, Medial part	VAM	Left		*n.s*		*n.s*	3.66	$p_{FDR} = 0.023$		*n.s*
MedioDorsalthalamic nucleus, Lateral part	MDL	Left		*n.s*		*n.s*		*n.s*	3.81	$p_{FDR} = 0.039$
Reticular thalamic nucleus	R#4	Left		*n.s*		*n.s*		*n.s*	3.83	$p_{FDR} = 0.037$
Lateral Geniculate Nucleus	LGN	Left		*n.s*		*n.s*	4.08	$p_{FDR} = 0.011$		*n.s*
		Right		*n.s*		*n.s*	3.49	$p_{FDR} = 0.030$		*n.s*
				n.s		*n.s*	3.45	$p_{FDR} = 0.033$		*n.s*
Paraseptal subpallium										
Accumbens nucleus	Acb	Left		*n.s*		*n.s*		*n.s*	4.38	$p_{FDR} = 0.018$
Midbrain										
Midbrain	MB	Right		*n.s*		*n.s*	4.43	$p_{FDR} = 0.006$		*n.s*
Cerebellum										
Cerebellum	Cb	Left	4.83	$p_{FDR} = 0.014$		*n.s*	3.32	$p_{FDR} = 0.039$		*n.s*
		midline		*n.s*		*n.s*			4.21	$p_{FDR} = 0.020$
		Right	3.57	$p_{FDR} = 0.033$		*n.s*	3.65	$p_{FDR} = 0.023$		*n.s*
				n.s		*n.s*	3.64	$p_{FDR} = 0.023$		*n.s*

<u>Table S6</u>: Cerebral activations for the global effect

fMRI activations for the global effect in the awake, anesthesia and high central thalamic (CT) DBS condition and comparison between high CT-DBS versus anesthesia. For the global effect, no regions are significantly different for the awake > high CT-DBS comparison. Group results, $p < 0.05$, FDR corrected, ns: non significant.

Cerebral areas labelling using the CIVM macaque brain atlas Revised version

Level	Labels CIVM	Abbreviations CIVM	Value CIVM
ventricles	ventricles	LV	1
medulla	medulla	Md	2
pons	pons	Pn	3
cerebellum	cerebellum	Cb	4
midbrain	midbrain	MB	5
thalamus	anterior pulvinar	Apul	6
thalamus	anteromedial thalamic nucleus	AM#1	7
thalamus	anteroventral thalamic nucleus	AV	8
thalamus	central medial thalamic nucleus	CM#2	9
thalamus	centrolateral thalamic nucleus	Cl#2	10
thalamus	centromedian thalamic nucleus, lateral part	CMnL	11
thalamus	centromedian thalamic nucleus, medial part	CMnM	12
thalamus	inferior pulvinar	Ipul	13
thalamus	interanteromedial thalamic nucleus	IAM	14
thalamus	intermediodorsal thalamic nucleus	IMD	15
thalamus	lateral dorsal thalamic nucleus, superficial part	LDSF	16
thalamus	lateral geniculate nucleus	LGN	17
thalamus	lateral pulvinar	Lpul	18
thalamus	medial geniculate nucleus, dorsal part	MGD	19
thalamus	medial geniculate nucleus, medial part	MGM	20
thalamus	medial geniculate nucleus, ventral part	MGV	21
thalamus	medial pulvinar	MPul	22
thalamus	mediodorsal thalamic nucleus, central part	MDC	23
thalamus	mediodorsal thalamic nucleus, dorsal part	MDD	24
thalamus	mediodorsal thalamic nucleus, lateral part	MDL	25
thalamus	mediodorsal thalamic nucleus, medial part	MDM	26
thalamus	parafascicular thalamic nucleus	Pf#2	27
thalamus	paraventricular thalamic nucleus	PV	28
thalamus	paraventricular thalamus	PVT	29
thalamus	pulvinar nuclei	Pul#1	30
thalamus	reticular thalamic nucleus	R#4	31
thalamus	reuniens thalamic nucleus	Re	32
thalamus	suprageniculate thalamic nucleus	SG	33
thalamus	entral anterior thalamic nucleus, lateral part, pallidal territory of thalamu	VAL(pal)	34
thalamus	ventral anterior thalamic nucleus, lateral part, ventralis oralis nucleus	VAL(VO)	35
thalamus	ventral anterior thalamic nucleus, magnocellular part	VAMC	36
thalamus	ventral anterior thalamic nucleus, medial part	VAM	37
thalamus	ventral lateral thalamic nucleus, lateral part	VLL	38
thalamus	ventral lateral thalamic nucleus, medial part	VLM#1	39
thalamus	ventral posterolateral thalamic nucleus	VPL#1	40
thalamus	ventral posteromedial thalamic nucleus	VPM	41
hypothalamus	hypothalamus	Hy	42
central subpallium	bed nucleus of the stria terminalis central division	BST	43
pallidum	external globus pallidus	EGP	44
pallidum	internal globus pallidus	IGP	45
pallidum	ventral pallidum	VP#3	46
paraseptal subpallium	accumbens nucleus, core	AcbC	47
paraseptal subpallium	accumbens nucleus, shell	AcbSh	48
paraseptal subpallium	accumbens nucleus	Acb	49
paraseptal subpallium	basal nucleus (Meynert)	BM#4	50
paraseptal subpallium	nucleus of the horizontal limb of the diagonal band	HDB	51
paraseptal subpallium	substantia innominata	SI	52
striatum	caudate nucleus	Cd	53
striatum	olfactory tubercle	Tu	54
striatum	putamen	Pu	55
subpallial amygdala	amygdalohippocampal area	AHi	56
subpallial amygdala	anterior amygdaloid area	AA	57
subpallial amygdala	bed nucleus of the stria terminalis intraamygdaloid division	BSTIA	58
subpallial amygdala	central amygdaloid nucleus, lateral division	CeL	59
subpallial amygdala	central amygdaloid nucleus, medial division	CEm#1	60
subpallial septum	septum	Se	61
lateral pallium	dorsal endopiriform nucleus	DEn	62
ventral pallium	amygdalohippocampal area, magnocellular part	AHiMC	63
ventral pallium	amygdalohippocampal area, parvicellular part	AHiPC	64
ventral pallium	amygdalopiriform transition area	APir	65
ventral pallium	anterior olfactory nucleus	AO	66
ventral pallium	basolateral amygdaloid nucleus	BL#2	67
ventral pallium	basolateral amygdaloid nucleus, dorsal part	BLD	68
ventral pallium	basolateral amygdaloid nucleus, intermediate part	BLI	69
ventral pallium	basolateral amygdaloid nucleus, ventral part	BLV	70
ventral pallium	basolateral amygdaloid nucleus, ventrolateral part	BLVL	71
ventral pallium	basolateral amygdaloid nucleus, ventromedial part	BLVM	72
ventral pallium	basomedial amygdaloid nucleus	BM#3	73
ventral pallium	lateral amygdaloid nucleus	La#3	74
ventral pallium	medial amygdaloid nucleus	Me	75
ventral pallium	paralaminar amygdaloid nucleus	PaL	76
ventral pallium	piriform cortex	Pir	77
ventral pallium	ventral cortical amygdaloid nucleus	VCo	78
hippocampus	hippocampus	Hip	79
medial pallium	parasubiculum	PaS	80
medial pallium	presubiculum	PrS	81
medial pallium	prosubiculum	ProS#2	82
medial pallium	subiculum	S#1	83
insular cortex	agranular insular cortex	AI	84
insular cortex	dysgranular insular cortex	DI#2	85
insular cortex	granular insular cortex	GI	86
insular cortex	insular proisocortex	IPro	87
cingulate cortex	area 23 of cortex	23	88
cingulate cortex	area 23a of cortex	23a	89
cingulate cortex	area 23b of cortex	23b	90
cingulate cortex	area 23c of cortex	23c	91
cingulate cortex	area 24/23a of cortex	24/23a	92
cingulate cortex	area 24/23b of cortex	24/23b	93
cingulate cortex	area 24/23c of cortex	24/23c	94
cingulate cortex	area 24a of cortex	24a	95
cingulate cortex	area 24b of cortex	24b	96
cingulate cortex	area 24c of cortex	24c	97
cingulate cortex	area 24d of cortex	24d	98
cingulate cortex	area 29a of cortex	29a	99
cingulate cortex	area 29a-c of cortex	29a-c	100
cingulate cortex	area 29d of cortex	29d	101
cingulate cortex	area 30 of cortex	30	102
cingulate cortex	area 31 of cortex	31	103
cingulate cortex	area 32 of cortex	32	104
cingulate cortex	area PGM/31 of cortex	PGM/31	105
cingulate cortex	parietal area PE, cingulate part	PECg	106
cingulate cortex	unnamed region sections 102-114, called 23 more anteriorly	23x	107
occipital cortex	juxtastriate area	JxSt	108
occipital cortex	prostriate area	ProST	109
occipital cortex	visual area 1 (primary visual cortex)	V1	110
occipital cortex	visual area 2	V2	111
occipital cortex	visual area 3, dorsal part	V3D	112
occipital cortex	visual area 3, ventral part	V3V	113
occipital cortex	visual area 3A	V3A	114
parietal cortex	area 1 of cortex (somatosensory)	1#1	115
parietal cortex	areas 2/1 of cortex (somatosensory)	39844	116
parietal cortex	area 2 of cortex (somatosensory)	2#1	117
parietal cortex	area 2 of cortex, vestibular part	2Ve	118
parietal cortex	area 3a of cortex (somatosensory)	3a	119
parietal cortex	area 3b of cortex (somatosensory)	3b	120
parietal cortex	depth intraparietal area	DIP	121
parietal cortex	dorsal parietal area	Dpt	122
parietal cortex	occipitoparietal area	OPt	123
parietal cortex	parietal area PE	PE#1	124
parietal cortex	parietal area PE, caudal part	PEC	125
parietal cortex	parietal area PEa	PEa	126
parietal cortex	parietal area PF (cortex)	PFCx	127
parietal cortex	parietal area PF, opercular part	PFOp	128
parietal cortex	parietal area PFG	PFG#1	129
parietal cortex	parietal area PG	PG#1	130
parietal cortex	parietal area PG, opercular part	PGOp	131
parietal cortex	parietal area POa, external part	POaE	132
parietal cortex	parietal area POa, internal part	POaI	133
parietal cortex	parieto-occipital area	PO#1	134
parietal cortex	parietooccipital associated area in the intraparietal sulcus	POa	135
parietal cortex	posterior parietal area	PPt#1	136
parietal cortex	retroinsular area, parietal part	ReIP	137
parietal cortex	secondary somatosensory cortex	S2	138
parietal cortex	secondary somatosensory cortex, external part	S2E	139
parietal cortex	secondary somatosensory cortex, internal part	S2I	140
parietal cortex	visual area 4, dorsal part	V4D	141
parietal cortex	visual area 4, transitional part	V4T	142
parietal cortex	visual area 4, ventral part	V4V	143
parietal cortex	visual area 4A	V4A	144

Labels CIVM_R	Abbrevations CIVM_R	Value CIVM_R left	Value CIVM_R
		0	0
MEDULLA	Md	1	2
PONS	Pn	3	4
CEREBELLUM	Cb	5	6
MIDBRAIN	MB	7	8
PULVINAR	Pul	9	10
ANTERO THALAMIC NUCLEUS	A#1	11	12
CENTRAL MEDIAL THALAMIC NUCLEUS	CM#2	13	14
CENTROLATERAL THAKAMIC NUCLEUS	Cl#2	15	16
CENTROMEDIAN THALAMIC NUCLEUS	CMN	17	18
PULVINAR	Pul	9	10
INTERANTEROMEDIAL THALAMIC NUCLEUS	IAM	19	20
INTERMEDIODORSAL THALAMIC NUCLEUS	IMD	21	22
LATERAL DORSAL THALAMIC NUCLEUS	LDSF	23	24
LATERAL GENICULATE NUCLEUS	LGN	25	26
PULVINAR	Pul	9	10
MEDIAL GENICULATE NUCLEUS	MG	27	28
PULVINAR	Pul	9	10
MEDIODORSAL THALAMIC NUCLEUS	MD	29	30
PARAFASCICULAR THALAMIC NUCLEUS	Pf#2	31	32
PARAVENTRICULAR THALAMIC NUCLEUS	PV	33	34
PULVINAR	Pul	9	10
RETICULAR THALAMIC NUCLEUS	R#4	35	36
		0	0
SUPRAGENICULATE THALAMIC NUCLEUS	SG	37	38
VENTRAL ANTERIOR THALAMIC NUCLEUS	VAL	39	40
VENTRAL LATERAL THALAMIC NUCLEUS	VL	41	42
VENTRAL POSTEROLATERAL THALAMIC NUCLEUS	VPL	43	44
	VPL	43	44
HYPOTHALAMUS	Hy	45	46
		0	0
EXTERNAL GLOBUS PALLIDUS	EGP	47	48
INTERNAL GLOBUS PALLIDUS	IGP	49	50
VENTRAL PADDIDUM	VP#3	51	52
ACCUMBENS NUCLEUS	Acb	53	54
	Acb	53	54
	Acb	53	54
MEYNERT BASAL NUCLEUS	BM#4	55	56
UCLEUS OF HORIZONTAL LIMB OF DIAGONAL BAN	HDB	57	58
		0	0
CAUDATE NUCLEUS	Cd	59	60
OLFACTORY TUBERCLE	Tu	61	62
PUTAMEN	Pu	63	64
AMYGDALA	AMY	65	66
		0	0
		0	0
AMYGDALA	AMY	65	66
HIPPOCAMPUS	Hip	67	68
SUBICULAR COMPLEX	PS	69	70
INSULAR CORTEX	IC	71	72
AREA 23 OF CORTEX	23	73	74
AREA 24/23a OF CORTEX	24/23a	75	76
AREA 24a OF CORTEX	24	77	78
AREA 29a OF CORTEX	29	79	80
AREA 30 OF CORTEX	30	81	82
AREA 31 OF CORTEX	31	83	84
AREA 32 OF CORTEX	32	85	86
AREA PGM/31 OF CORTEX	PGM/31	87	88
PARIETAL AREA PE, cingulate part	PECg	89	90
		0	0
JUXTASTRIATE AREA	JxSt	91	92
PROSTRIATE AREA	ProST	93	94
VISUAL AREA 1	V1	95	96
VISUAL AREA 2	V2	97	98
VISUAL AREA 3	V3	99	100
AREA 1 OF CORTEX	1#1	101	102
AREA 2/1 OF CORTEX	2#1	103	104
AREA 2 OF CORTEX	2	105	106
AREA 3a OF CORTEX	3	107	108
DEPTH INTRAPARIETAL AREA	DIP	109	110
DORSAL PARIETAL AREA	Dpt	111	112
OCCIPITOPARIETAL AREA	OPt	113	114
PARIETAL AREA PE	PE	115	116
PARIETAL AREA PF	PFCx	117	118
PARIETAL AREA PFG	PFG#1	119	120
PARIETAL AREA PG	PG#1	121	122
PARIETAL AREA Poa	POa	123	124
PARIETO-OCCIPITAL AREA	PO#1	125	126
POSTERIOR PARIETAL AREA	PPt#1	127	128
RETROINSULAR AREA, parietal part	ReIP	129	130
SECONDARY SOMATOSENSORY CORTEX	S2	131	132
VISUAL AREA 4	V4	133	134

continue

2/2

Cerebral areas labelling using the CIVM macaque brain atlas Revised version

Level	Labels CIVM	Abbreviations CIVM	Value CIVM
temporal cortex	area 35 of cortex	35	145
temporal cortex	area PG associated region of the superior temporal sulcus	PGa	146
temporal cortex	area TF, medial part	TFM	147
temporal cortex	area TL, rostral part (area 36R)	TLR(R36)	148
temporal cortex	auditory koniocortex, lateral part	AKL	149
temporal cortex	auditory koniocortex, medial part	AKM	150
temporal cortex	entorhinal cortex, caudal limited part	ECL	151
temporal cortex	entorhinal cortex, caudal part	EC#2	152
temporal cortex	entorhinal cortex, lateral part, caudal division	ELC	153
temporal cortex	entorhinal cortex, lateral part, rostral division	ELR	154
temporal cortex	entorhinal cortex, rostral part	ER#1	155
temporal cortex	entorhinal Cx, intermediate part	EI	156
temporal cortex	entorhinal Cx, olfactory part	EOl	157
temporal cortex	fundus of superior temporal sulcus	FST	158
temporal cortex	intraparietal sulcus associated area in the superior temporal sulcus	IPa	159
temporal cortex	medial superior temporal area	MST	160
temporal cortex	middle temporal area (visual area 5)	MT(V5)	161
temporal cortex	paraauditory area, caudal part	PaAC	162
temporal cortex	paraauditory area, lateral part	PaAL	163
temporal cortex	paraauditory cortex, rostral part	PaAR	164
temporal cortex	parainsular cortex, lateral part	PaIL	165
temporal cortex	parainsular cortex, medial part	PaIM	166
temporal cortex	prokoniocortex	ProK	167
temporal cortex	prokoniocortex, lateral part	ProKL	168
temporal cortex	prokoniocortex, medial part	ProKM	169
temporal cortex	retroinsular area	ReI	170
temporal cortex	retroinsular area, temporal part	ReIT	171
temporal cortex	superior temporal sulcus area 1	ST1	172
temporal cortex	superior temporal sulcus area 3	ST3	173
temporal cortex	superior temporal sulcus area, gyral part	ST2g	174
temporal cortex	superior temporal sulcus area, sulcal part	ST2s	175
temporal cortex	temporal area TAa	TAa	176
temporal cortex	temporal area TE, medial part	TEM	177
temporal cortex	temporal area TE, occipital part	TEO	178
temporal cortex	temporal area TE, occipitomedial part	TEOM	179
temporal cortex	temporal area TE1	TE1#1	180
temporal cortex	temporal area TE2	TE2#1	181
temporal cortex	temporal area TE3	TE3#1	182
temporal cortex	temporal area TEa	TEa#1	183
temporal cortex	temporal area TF	TF	184
temporal cortex	temporal area TF, lateral part	TFL	185
temporal cortex	temporal area TF, occipital part	TFO	186
temporal cortex	temporal area TH	TH	187
temporal cortex	temporal area TH, occipital part	Tho	188
temporal cortex	temporal area TL	TL	189
temporal cortex	temporal area TL, occipital part (area 36O)	TLO(36O)	190
temporal cortex	temporal parietooccipital associated area in sts	TPO	191
temporal cortex	temporal parietooccipital associated area in sts, caudal part	TPOC	192
temporal cortex	temporoparietal cortex	Tpt	193
temporal cortex	temporopolar periallocortex	TTPAl	194
temporal cortex	temporopolar proisocortex	TPPro	195
frontal cortex	area 4 of cortex (primary motor)	4#1	196
frontal cortex	area 6 of cortex, dorsocaudal part (Matellis F2)	6DC(F2)	197
frontal cortex	area 6 of cortex, dorsorostral part (Matellis F7)	6DR(F7)	198
frontal cortex	area 6 of cortex, medial (supplementary motor) part	6M	199
frontal cortex	area 6 of cortex, ventral part, caudal subdivision (Matellis F4)	6VC(F4)	200
frontal cortex	area 6 of cortex, ventral part, rostral subdivision (Matellis F5)	6VR(F5)	201
frontal cortex	area 6/32 of cortex	10379	202
frontal cortex	area 8 of cortex, anterodorsal part	8AD	203
frontal cortex	area 8 of cortex, anteroventral part	8AV	204
frontal cortex	area 8/32 of cortex	10440	205
frontal cortex	area 8A of cortex	8A	206
frontal cortex	area 8B of cortex	8B	207
frontal cortex	area 9 of cortex, lateral part	9L	208
frontal cortex	area 9 of cortex, medial part	9M	209
frontal cortex	area 9/32 of cortex	10471	210
frontal cortex	area 9/46 of cortex	15584	211
frontal cortex	area 9/46 of cortex, dorsal part	9/46D	212
frontal cortex	area 9/46 of cortex, ventral part	9/46V	213
frontal cortex	area 10 of cortex	10	214
frontal cortex	area 10 of cortex, dorsal part	10D	215
frontal cortex	area 10 of cortex, medial part	10M	216
frontal cortex	area 10 of cortex, ventral part	10V	217
frontal cortex	area 44 of cortex	44	218
frontal cortex	area 45A of cortex	45A	219
frontal cortex	area 45B of cortex	45B	220
frontal cortex	area 46D of cortex	46D	221
frontal cortex	area 46V of cortex	46V	222
frontal cortex	area 47 (old 12) of cortex	47(12)	223
frontal cortex	area 47 (old 12) of cortex, lateral part	47(12)L	224
frontal cortex	area 47 (old 12) of cortex, orbital part	47(12)O	225
frontal cortex	area ProM (promotor)	proM#1	226
frontal cortex	gustatory cortex	Gu	227
orbitofrontal cortex	area 11 of cortex	11	228
orbitofrontal cortex	area 11 of cortex, lateral part	11L	229
orbitofrontal cortex	area 11 of cortex, medial part	11m	230
orbitofrontal cortex	area 13 of cortex	13	231
orbitofrontal cortex	area 13 of cortex, lateral part	13L	232
orbitofrontal cortex	area 13 of cortex, medial part	13M	233
orbitofrontal cortex	area 13a of cortex	13a	234
orbitofrontal cortex	area 14 of cortex, medial part	14M	235
orbitofrontal cortex	area 14o	14o	236
orbitofrontal cortex	area 25 of cortex	25	237
orbitofrontal cortex	orbital periallocortex	OPAl	238
orbitofrontal cortex	orbital proisocortex	OPro	239
white matter	cerebral white matter	cwm	240
white matter	external medullary lamina	eml	241

Labels CIVM_R	Abbrevations CIVM_R	Value CIVM_R left	Value CIVM_R
AREA 35 OF CORTEX	35	135	136
REA PG, associated region of superior temporal sulc	PGa	137	138
AREA TF, medial part	TFM	139	140
AREA TL, rostral part (area 36R)	TLR(R36)	141	142
AUDITORY KONIOCORTEX	AK	143	144
ENTORHINAL CORTEX	ECL	145	146
FUNDUS OF SUPERIOR TEMPORAL SULCUS	FST	147	148
INTRAPARIETAL SULCUS ASSOCIATED AREA	IPa	149	150
MEDIAL SUPERIOR TEMPORAL AREA	MST	151	152
MIDDLE TEMPORAL AREA (visual area 5)	MT(V5)	153	154
PARAAUDITORY CORTEX	PaA	155	156
PARAINSULAR CORTEX	PaI	157	158
PROKONIOCORTEX	ProK	159	160
RETROINSULAR AREA	ReI	161	162
SUPERIOR TEMPORAL SULCUS	ST1	163	164
TEMPORAL AREA TAa	TAa	165	166
TEMPORAL AREA TE	TE	167	168
TEMPORAL AREA TF	TF	169	170
TEMPORAL AREA TH	TH	171	172
TEMPORAL AREA TL	TL	173	174
TEMPORAL PARIETOOCCIPITAL ASSOCIATED AREA	TPO	175	176
TEMPOROPARIETAL CORTEX	Tpt	177	178
TEMPOROPOLAR PERIALLOCORTEX	TTPAl	179	180
TEMPOROPOLAR PROISOCORTEX	TPPro	181	182
AREA 4 OF CORTEX (primary motor)	4#1	183	184
AREA 6 OF CORTEX	6	185	186
AREA 6/32 OF CORTEX	6/32	187	188
AREA 8 OF CORTEX	8	189	190
AREA 9 OF CORTEX	9	191	192
AREA 9/32 OF CORTEX	9/32	193	194
AREA 9/46 OF CORTEX	9/46	195	196
AREA 10 OF CORTEX	10	197	198
AREA 44 OF CORTEX	44	199	200
AREA 45 OF CORTEX	45	201	202
AREA 46 OF CORTEX	46	203	204
AREA 47 OF CORTEX	47	205	206
AREA PROM (promotor)	proM#1	207	208
GUSTATORY CORTEX	Gu	209	210
AREA 11 OF CORTEX	11	211	212
AREA 13 OF CORTEX	13	213	214
AREA 14 OF CORTEX	14	215	216
AREA 25 OF CORTEX	25	217	218
ORBITAL PERIALLOCORTEX	OPAl	219	220
ORBITAL PROISOCORTEX	OPro	221	222
		0	0
		0	0

Table S7: Whole brain areas labelling of the Center for In Vivo Microscopy atlas (CIVM) atlas Revised (CIVM_R) for functional correlations analysis.

Whole brain regions considered for the functional correlations analysis using the macaque CIVM atlas*(76)* that was revised into CIVM_R to match fMRI spatial resolution. Regions merged together (for instance dorsal, medial and ventral part of the medial geniculate nucleus in the original CIVM atlas into medial geniculate nucleus) share the same abbreviation and value in the CIVM_R. Brackets represent the biggest regions unified. Empty cells stands for deleted regions.
